# Discovery of Partner Protein-Dependent Graspetide Biosynthesis

**DOI:** 10.1021/acschembio.5c00957

**Published:** 2026-03-14

**Authors:** Riley S. Carter, Sangeetha Ramesh, Hamada Saad, Abdullah Mubarik, Douglas A. Mitchell

**Affiliations:** † Department of Chemistry, 14589 University of Illinois at Urbana–Champaign, Urbana, Illinois 61801, United States; ‡ Department of Microbiology, University of Illinois at Urbana–Champaign, 600 South Mathews Avenue, Urbana, Illinois 61801, United States; § Department of Biochemistry, 5718 Vanderbilt University School of Medicine, Nashville, Tennessee 37232, United States; ∥ School of Molecular and Cellular Biology, University of Illinois at Urbana–Champaign, Urbana, Illinois 61801, United States; ⊥ Department of Chemistry, Vanderbilt University, Nashville, Tennessee 37232, United States

## Abstract

Graspetides represent a class of ribosomally synthesized and post-translationally modified peptide (RiPP) that can contain macrolactone/macrolactam linkages from amino acid side chains. Many predicted graspetide biosynthetic gene clusters (BGCs) contain untapped tailoring enzymes, including some with the potential to modify macrocyclic peptide scaffolds. In this work, we investigated several of these BGCs and discovered the first examples of partner protein-dependent graspetide biosynthesis and the installation of an unprecedented cyclized 5-hydroxyisopeptide moiety. We first updated the bioinformatic tool RODEO to robustly identify diverse graspetides with additional tailoring enzymes. Using this algorithm to survey available genomic data, a data set of >20,000 predicted graspetides was generated and a large-scale bioinformatic analysis was performed on proteins in or near graspetide BGCs. From this analysis, two groups of graspetides with strictly conserved co-occurring proteins were prioritized for characterization. These graspetides contained novel ring connectivities and their biosynthesis was dependent on co-occurring partner proteins, a feature unprecedented among characterized graspetides. The first graspetide, rosaritide, features three interlocking macrolactone linkages. The activity and stability of the rosaritide graspetide synthetase was dependent on a partner protein which copurifies to form a catalytically active complex. The second graspetide, corallotide, is unusually large and contains five repeated motifs in which a Lys is first macrocyclized into an isopeptide bond and then hydroxylated at the δ-carbon by a divergent 2OG-Fe­(II)-dependent oxygenase. The biosynthesis and biosynthetic enzymes from these BGCs were then characterized in vitro. Overall, this study expands our understanding of graspetide biosynthesis and the ability to predict graspetide BGCs.

## Graphical abstract

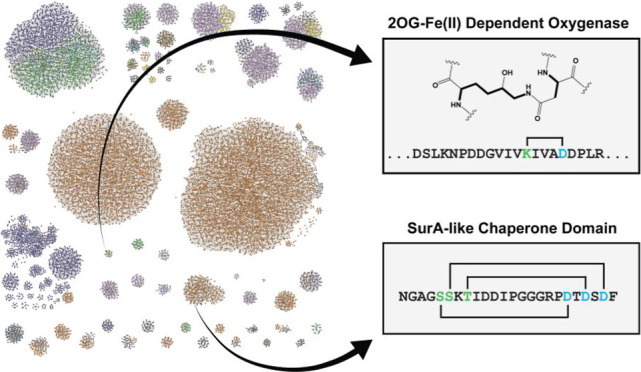

## Introduction

Macrocyclic peptides are attracting greater attention in the scientific community as a new source of potential therapeutics.^1^ Much of this interest stems from the ability of macrocyclic peptides to occupy the chemical space between small molecules and biologics. These macrocyclic peptides have the potential for tighter and more specific binding than small molecules due to their increased surface area and to overcome many of the challenges in production, purification, and storage that face biologic therapeutics.^2^ While chemical methods and workflows exist to generate peptides with a single macrocycle,^3^ installing two or more specific macrocycles in a peptide typically requires synthesizing a peptide with multiple, orthogonal cross-linking handles, or using an enzyme as a catalyst.^4^ Additionally, further chemical diversity of macrocyclic peptides can be accessed if more enzymes are discovered that can perform late-stage modifications on the cyclized peptides. As such, discovering and characterizing new enzymes capable of either installing multiple macrocycles in a peptide or functionalizing macrocyclic peptides have merit in furthering our ability to develop therapeutic candidates. A group of natural products called graspetides shows promise in fulfilling these criteria.

Graspetides are a class of ribosomally synthesized and post-translationally modified peptides (RiPPs) that contain macrocyclic linkages installed between amino acid side chains in a peptide (Figure 1A).^5^ RiPP biosynthesis involves a genetically encoded precursor peptide first being translated by the ribosome, followed by the installation of various modifications using tailoring enzymes. In graspetide biosynthesis, macrolactone and/or macrolactam linkages are installed between a nucleophilic “donor” residue (Ser, Thr, Lys) and a carboxylate-containing “acceptor” residue (Asp, Glu) by a group of ATP-grasp ligases termed graspetide synthetases.^5,6^ Graspetide synthetases form linkages by first phosphorylating a carboxylate using ATP and then guiding a nucleophile to attack the acyl phosphate intermediate.^5,7^ While some graspetide synthetases have shown a preference for the type of linkage they install, others can form both esters (macrolactones), amides (macrolactams), and non-native thioester linkages using various nucleophilic donor residues.^8–12^

**Figure 1:**
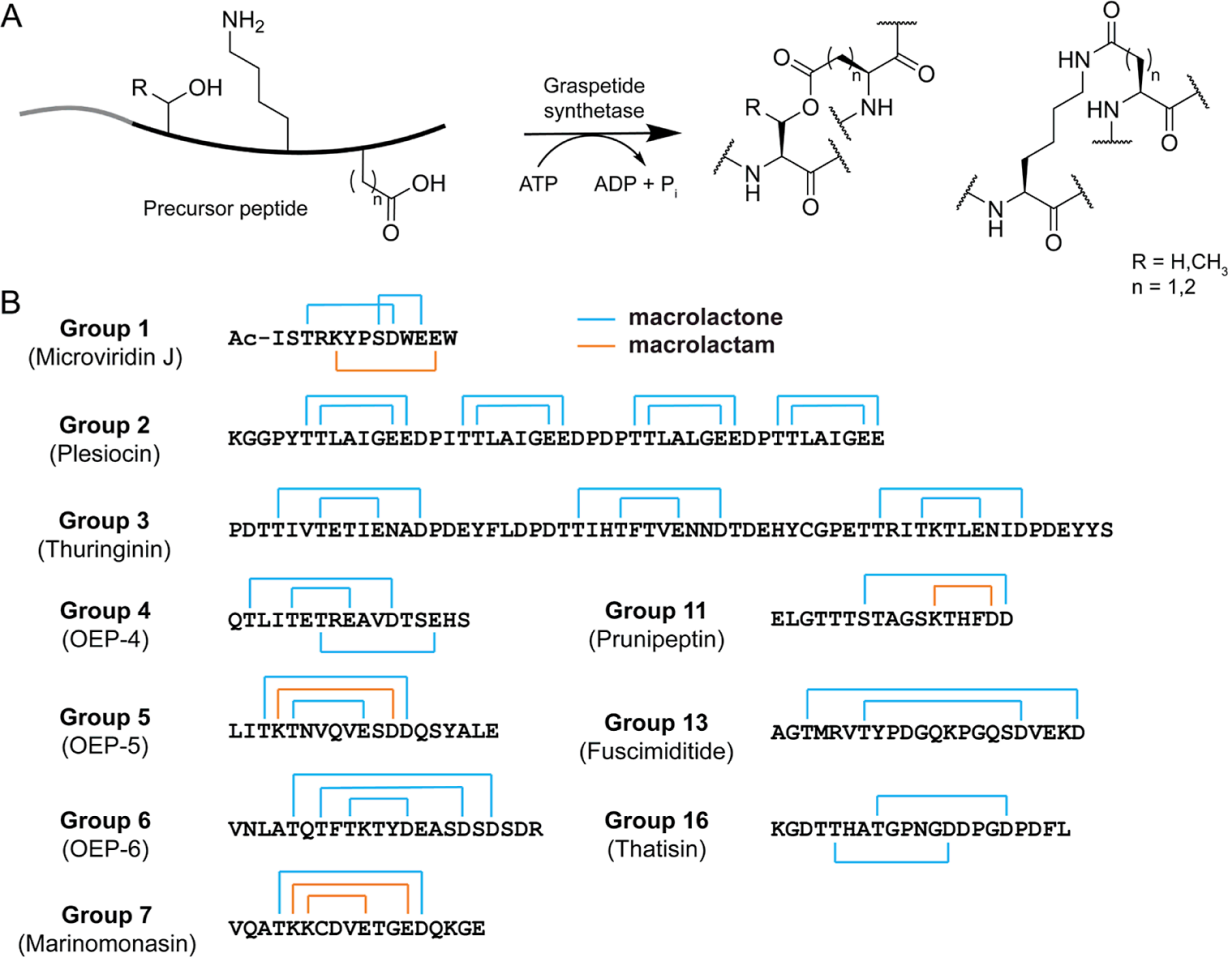
(A) Diagram of graspetide biosynthesis. (B) Sequence and ring connectivity of characterized graspetide groups. OEP, omega ester-containing peptide.

Characterized graspetide synthetases typically install multiple linkages within a single precursor peptide, producing multimacrocyclic structures with either an interlocked (i.e., cage-like) or a ring within ring (i.e., hairpin) connectivity (Figure 1B). The former is illustrated by the microviridins, which contain three interlocking rings that constrain the peptide into a “cage-like” structure. A hairpin pattern is exemplified by plesiocin, which contains multiple repeats of cyclized motifs. Due to the complex structures in these two molecules, both microviridins and plesiocin are potent serine protease inhibitors.^13–15^ Interestingly, the potency of plesiocin increases with the number of cyclized motifs,^14^ while the potency of some microviridins is modulated by proteolysis and acetylation.^16^ Many other RiPP classes contain secondary post-translational modifications (PTMs) that are critical for a reported bioactivity.^17,18^ Beyond the acetylation of microviridins as a secondary graspetide PTM,^20^ we are aware of only one additional example: methyl ester formation in graspimiditides, which can subsequently undergo spontaneous conversion to an aspartimide. A recent study further expanded the known chemistry of this class by identifying enzymes from a graspetide biosynthetic gene cluster that catalyze conversion of an Asp residue to an aminomalonate moiety on a linear precursor peptide.^19^ Altogether, secondary PTM-installing graspetide biosynthetic enzymes are understudied. As such, we undertook an investigation of graspetide BGCs from uncharacterized groups with additional predicted tailoring enzymes.

To identify graspetide BGCs with additional tailoring enzymes, we turned to the high-throughput genome-mining tool RODEO.^6,21–25^ The base function of RODEO is to retrieve the local genomic neighborhood of a given protein accession (or list of accessions), identify predicted ORFs in each neighborhood, annotate the predicted genes using HMM databases (e.g., PFAM, TIGRFAM), and collate the results into HTML or CSV files. Originally designed for lasso peptides, numerous additional RODEO modules have been developed that score and identify the precursor peptides for numerous RiPP classes.^26^ These features make this tool particularly useful in performing large-scale analyses of co-occurring proteins and RiPP precursor peptides nearby homologues for specific biosynthetic enzymes. A graspetide-scoring RODEO module was previously developed,^6^ which expanded the number of graspetide groups to 24. These groups are defined by conserved core peptide consensus sequences and putative ancillary modifications. However, in the four years since the release of the original graspetide RODEO scoring module, additional examples have been characterized that have revealed new insights into the class. We updated this scoring module to more robustly identify diverse BGCs and then used the updated module to survey predicted graspetide BGCs in the available genomic data. From this data set of predicted graspetides, we identified and characterized two BGCs with novel structures and an intriguing partner-protein-dependent biosynthesis.

## Results and Discussion

### Update to the Graspetide RODEO Module and Protein Co-Occurrence Analysis

Originally, the graspetide RODEO module was developed using biosynthetic information from characterized members of graspetide groups 1–6. Since that development, additional graspetide groups have been characterized, increasing our knowledge of graspetide biosynthesis (Figure 1). Using this information, we modified the graspetide module to more robustly identify diverse graspetide precursor peptides. This update included the addition of scoring metrics for the core peptide sequences in newly characterized graspetides, using regular expressions (regex) to increase the diversity of acceptor and donor residue patterns, and increased scoring for certain co-occurring proteins (Table S1). The support vector machine (SVM) classification was also retrained with the addition of new scoring heuristics (Table S2). The scoring metrics for the updated module were tested on a subset of high confidence positive and negative sequences to ensure the accuracy of the module (Figure S1). We then used the updated graspetide module to generate a data set of RODEO-curated graspetides using previously published methods (see Methods, Table S3, Supporting Information 1). While the graspetide survey and analysis using the original module identified 4356 predicted graspetides across 3923 BGCs, the updated data set contained 21,584 predicted graspetides across 17,967 BGCs, an almost 5-fold increase (Figure 2). In addition to identifying the majority (>75%) of BGCs found in the original 24 graspetide groups, the updated RODEO module identified 19 new groups of BGCs containing 20 or more members.

**Figure 2:**
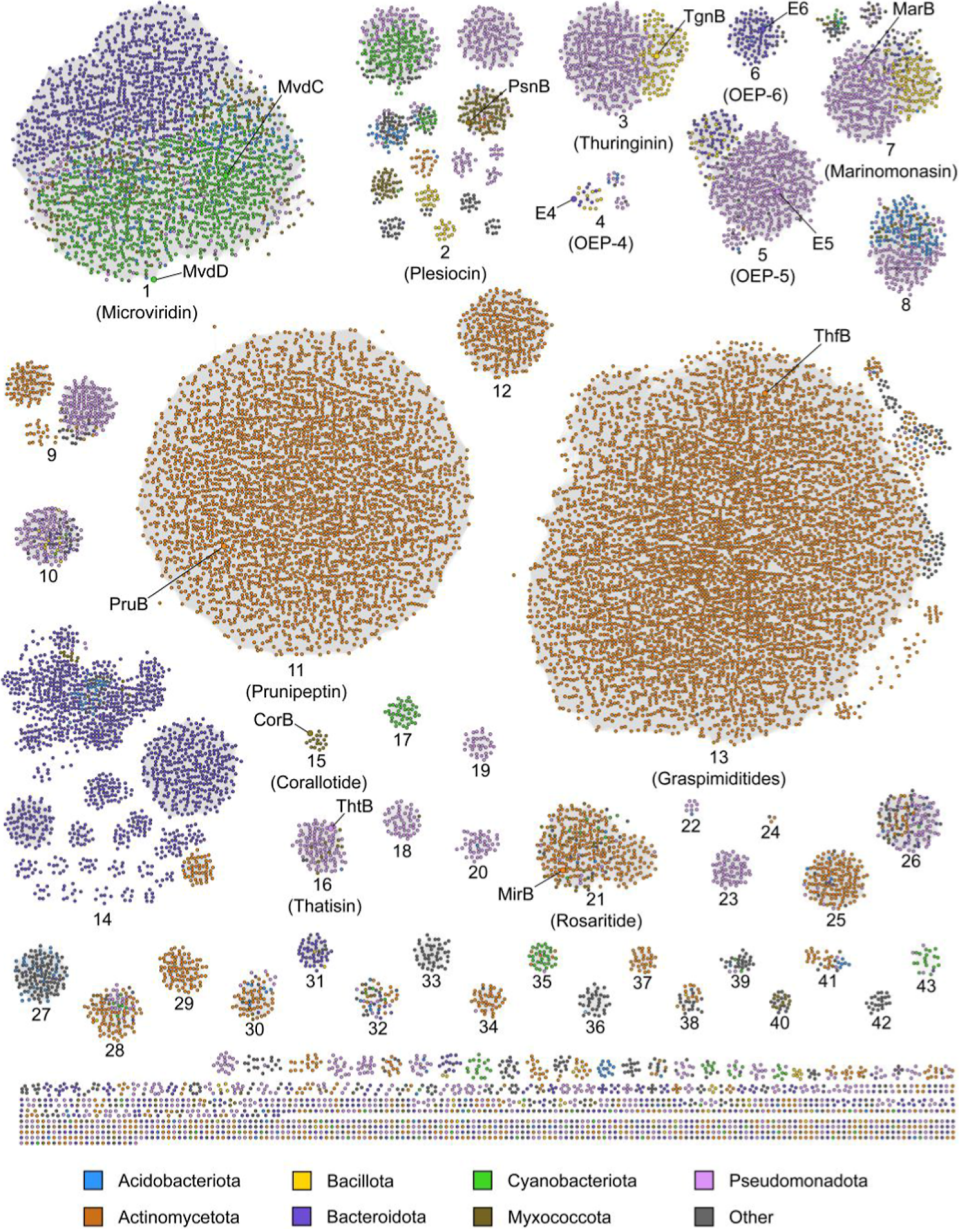
Sequence similarity network (SSN) of predicted graspetide synthetases with a RODEO-identified precursor peptide. An alignment score of 70 was used, which preserves the groups identified in previous studies. The graspetide groups are numbered based on the order of discovery and labeled based on prominent characterized graspetides from each group. Individual graspetide synthetases from these graspetides are also labeled. Groups are colored based on originating phylum. Supporting Information set 1 includes phylum, genus, and species assignments for all identified graspetides.

We performed an analysis of the co-occurring proteins in all the predicted graspetide groups (Table S4). This analysis yielded many new insights, especially within the newly identified groups (Figures S2–S20). Several of the new graspetide groups contain only graspetide synthetases and precursor peptides (groups 27, 28, and 35), but many others contain additional proteins that are domains of unknown function (DUFs) or that do not match any PFAM or TIGRFAM hidden Markov models (HMMs) (groups 25, 31, 34, 38, 42, and 43). Of these, groups 34 and 35 also often contain long precursor peptides with repeating motifs containing residues used in the formation of graspetide linkages. Additionally, many of these BGCs are from understudied organisms, such as groups 27 and 42, which include many examples from uncharacterized candidate bacterial phyla. The newly identified groups also contain many BGCs with diverse biosynthetic enzymes previously unseen in graspetide BGCs. Members of groups 29 and 37 often contain an additional protein predicted to be phosphotransferases and are found only in Actinomycetota. Groups 33 and 39 both contain proteins containing a predicted transglutaminase fold, which is also often associated with protease activity in prokaryotic contexts. Additionally, groups 26, 30, 32, and 40 contain a neighboring protease/peptidase/Peptidase-containing ABC transporter (PCAT), with group 32 containing diverse families of neighboring proteases. These proteases may be involved in graspetide maturation or function as resistance genes, both of which are promising avenues to discover bioactive graspetides. Members of group 36 are only found in Thermoprotea and often co-occur with a protein that weakly matches an HMM for a chromosomal segregation protein, which is unusual in the context of RiPP BGCs. Lastly, members of group 41 are taxonomically diverse and contain large precursor peptides co-occurring with a copper amine oxidase. Within many of these new graspetide groups are BGCs that contain more diverse proteins that co-occur at lower frequencies. This includes BGCs with enzymes characteristic of other natural products, such as adenylation domains, polyketide cyclases, prenyltransferases, and lanthipeptide dehydratases (Figure S21). Other examples are rich in oxidative and reductive enzymes, including dioxygenases, monooxygenases, oxidoreductases, and dehydrogenases (Figure S21). Overall, the new graspetide groups identified by the updated RODEO module contain diverse co-occurring proteins, many of which are from understudied organisms.

The gene co-occurrence of the previously identified groups also yielded intriguing insights. Group 1 graspetides often co-occur with AMP-binding proteins and/or an alpha/beta hydrolase protein, which may be responsible for further derivatization of those molecules. Group 7 BGCs often contain an acetyltransferase and/or a drug-efflux transporter, over half of the examples from group 9 graspetides contain a serine proteinase, and group 10 frequently contains a DNA helicase. The proteins from these groups may be duplicated housekeeping genes that are used for self-resistance.^27^ Group 13 comprises previously mentioned graspimidities that almost always co-occur with a protein isoaspartyl methyltransferase (PIMT).^19^ Additionally, groups 11 and 13 graspetides both may co-occur with genes for a DUF397 and DUF5753 protein, which are known to function as xenobiotic response element (XRE)-regulator pairs in Actinomycetota.^28^ Not only do these BGCs show promise in finding bioactive graspetides, but also genome mining for DUF397-DUF5753 pairs may open the door to discovering new types of natural product biosynthetic enzymes from Actinomycetota. Group 14 members all co-occur with a SPASM-domain containing protein, which typically contains two auxiliary [4Fe–4S] centers and are often associated with radical *S*-adenosylmethionine (rSAM) enzymes.^29^ Additionally, groups 11, 12, 16, 17, 18, 20, and 23 also have a high co-occurrence of various DUFs. Of these, DUF6624 was found in groups 18, 20, 23, and 42 at high frequencies and may represent an undiscovered class of biosynthetic enzyme. Group 18 also has a high co-occurrence of oxygenases, as observed with the recent discovery of an oxygenase pair that installed an aminomalonic acid moiety on a linear graspetide precursor peptide from group 18.^30^ Groups 2, 8, 14, 16, 18, 20, 21, and 23 all have high co-occurrence with a nitrile hydratase leader microcin (NHLM)-type transporter. In RiPP biosynthesis, these transporters often contain a peptidase that removes the leader sequence as part of the maturation before export from the cell.

Of particular interest were graspetide groups that contained suspected secondary PTM-installing enzymes with very high co-occurrence in the group. Given the observation of *cis*/*trans* Pro-mediated conformational isomerism in group 16 graspetides^6^ (thatisin and *iso*-thatisin), we were intrigued to find that graspetide BGCs from group 21 contained a predicted divergent PpiC-type peptidylprolyl isomerase (PPIase). As the precursor peptides also contained a unique residue pattern that was suggestive of new ring connectivity, we chose to characterize an example from this group. We next examined BGCs from graspetide group 15, which all encode a predicted divergent 2-oxoglutarate (2OG)-Fe­(II)-dependent oxygenase, a predicted β-lactamase, an unusually large precursor peptide, and graspetide synthetase. Characterized 2OG-Fe­(II)-dependent oxygenases perform hydroxylation, halogenation, and ring-forming reactions; thus, their inclusion in group 15 BGCs was expected to increase the chemical diversity of graspetide scaffolds.^31,32^ Additionally, we observed that the BGC architecture of this graspetide group was highly conserved and restricted to the genus *Corallococcus*, and homologues of the oxygenase only appeared in this graspetide BGC architecture. As such, we also selected an example from this group to characterize.

### Characterization of a PPIase-Associated Graspetide, Rosaritide

From group 21, we chose the example from *Micromonospora rosaria* NRRL 3718 that contained genes encoding a precursor peptide (MirA, NCBI accession ID: WP_083979189.1), a graspetide synthetase (MirB, WP_067373627.1, TIGR04187), a predicted PpiC-type peptidylprolyl isomerase (MirC, WP_067373628.1, TIGR04500), and a PCAT (MirD, WP_169807199.1, TIGR03796) (Figure 3). We obtained *Escherichia coli* codon-optimized genes for the BGC and expressed MirA as a tobacco etch virus (TEV) protease-cleavable, maltose-binding protein (MBP) fusion along with MirB and MirC in *E. coli* BL21 (DE3). We then purified MBP-tagged MirA by amylose-affinity purification and removed the MBP tag using TEV protease. Upon analysis by matrix-assisted laser desorption/ionization time-of-flight mass spectrometry (MALDI-TOF MS), we observed a mass loss of up to 54 Da compared to a control expressing only MirA (Figure 4). This corresponds to three dehydrations, consistent with three graspetide linkages. MirA contains a predicted PCAT cut site, which helped us to identify the core peptide sequence. However, to simplify the structural characterization, we removed the leader peptide by trypsinolysis which provided the triply dehydrated MirA core peptide lacking residues 1–3. We named the triply dehydrated core peptide rosaritide after the source organism, *M. rosaria*, and termed the product lacking the first three core residues rosaritide_trunc_ for simplicity in discussion.

**Figure 3:**
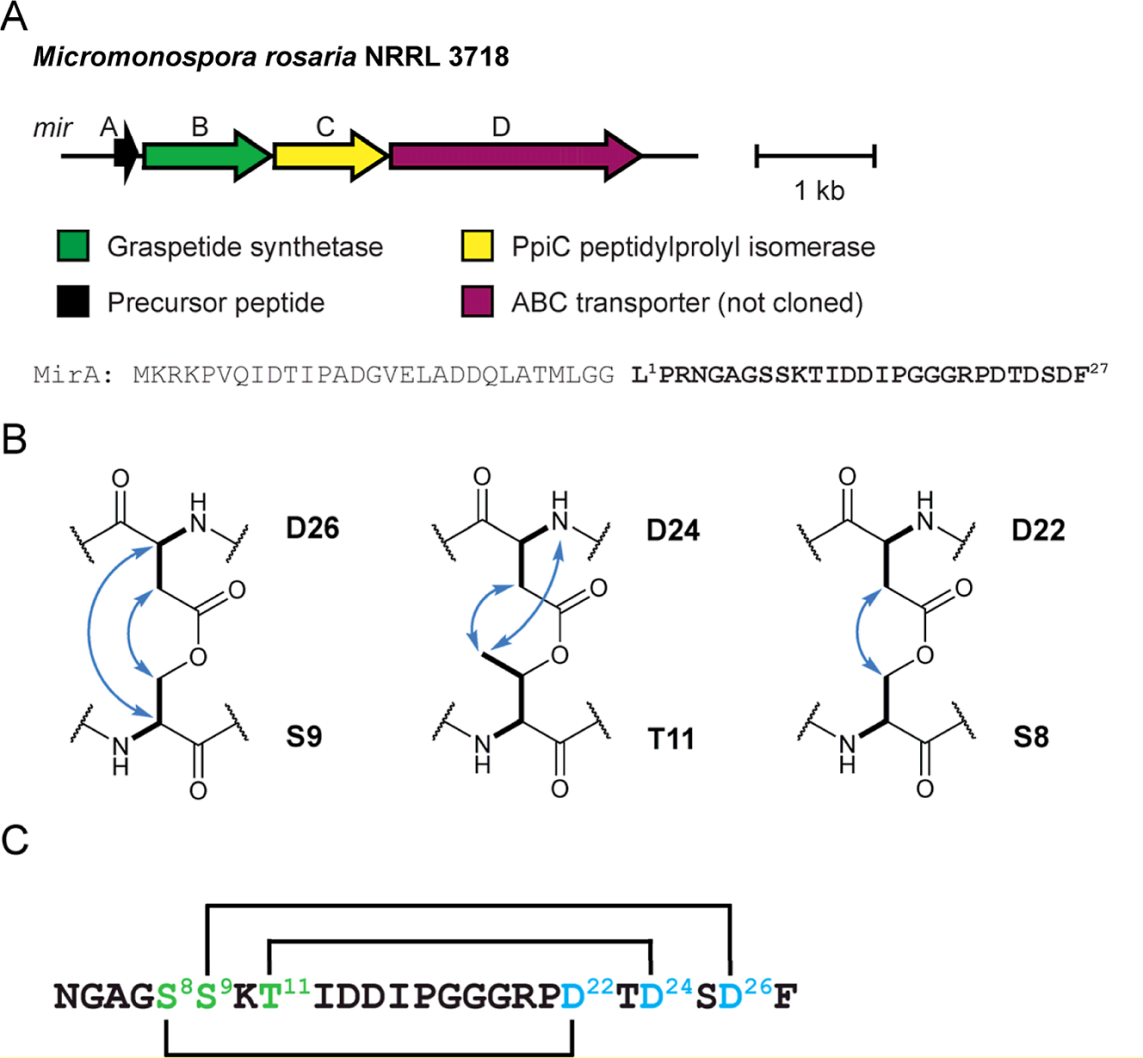
Rosaritide BGC, sequence, and linkage pattern. (A) Rosaritide BGC from *M*.*rosaria* NRRL 3718. The MirA precursor peptide sequence is shown with the predicted core peptide in bold. (B) NOESY correlations between cross-linked residues in rosaritide_trunc_. (C) Rosaritide_trunc_ linkage connectivity, with donor residues labeled green and acceptor residues labeled blue.

**Figure 4:**
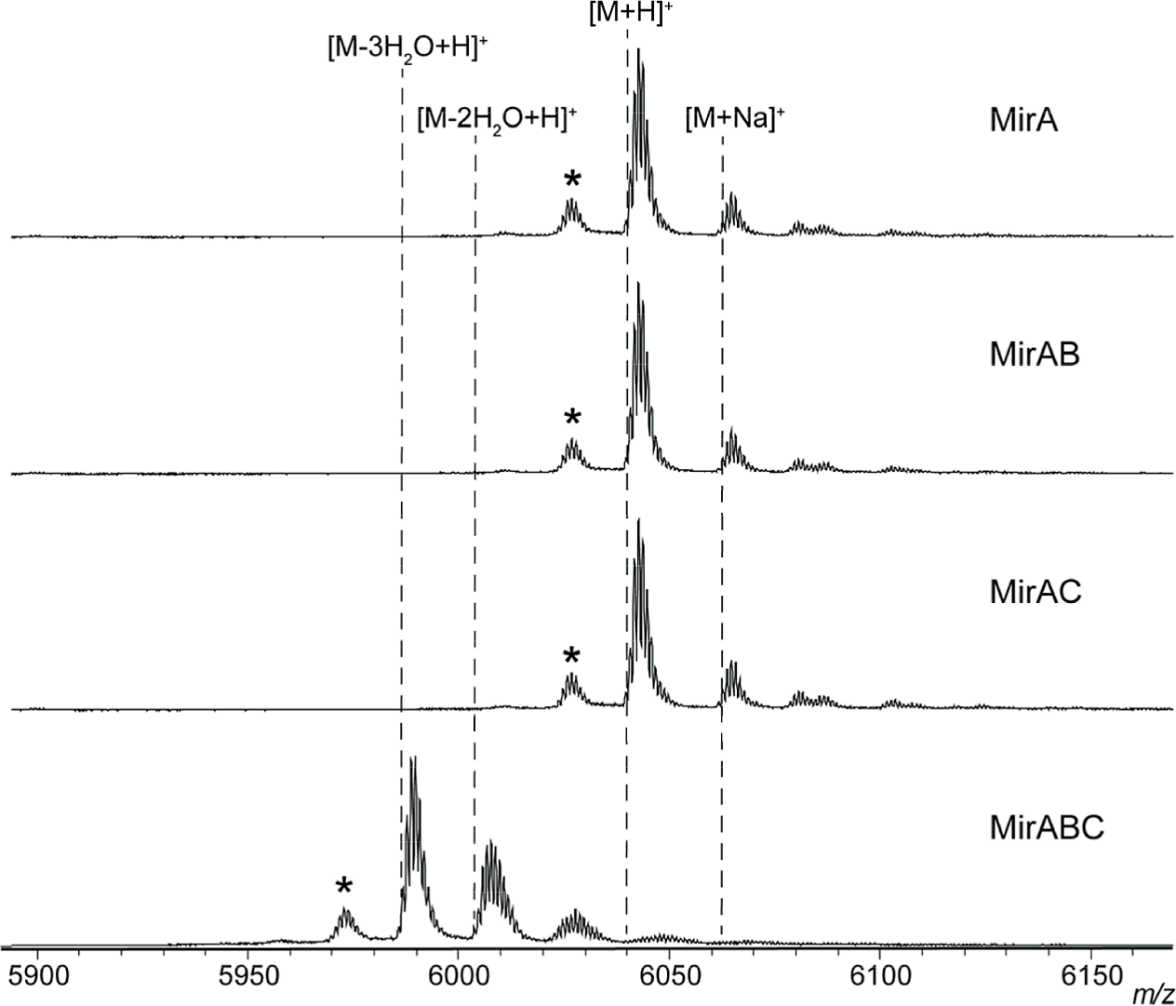
Rosaritide partner protein-dependent biosynthesis. MALDI-TOF mass spectra of MirA expressed either alone or in combination with various biosynthetic enzymes. Asterisks denote peaks from laser-induced deamination.

To discriminate between macrolactone and macrolactam linkages, we subjected rosaritide_trunc_ to methanolysis under alkaline conditions. Methanolysis under mildly alkaline conditions selectively cleaves macrolactones and labels the acceptor residue with a methyl group, allowing for localization of acceptor residue by tandem MS. Upon methanolysis, rosaritide_trunc_ produced masses consistent with up to three additions of 32 Da, showing that all three linkages formed were macrolactones (Figure S22). Analysis of fully methanolized rosaritide_trunc_ (+96 Da) by high-resolution and tandem mass spectrometry (HRMS/MS) localized the methyl groups to Asp22, Asp24, and Asp26 (Figure S23).

To confirm the identity of the acceptor residues labeled during alkaline methanolysis, we generated MirA variants in which all putative acceptor residues (Asp13, Asp14, Asp22, Asp24, and Asp26) were individually replaced with Ala. Following coexpression with MirB and MirC, purification, and proteolysis, the resultant products were analyzed by MALDI-TOF MS for any disruption in ring formation (Figure S24). As expected, a triply dehydrated product was not observed for the D22A, D24A, and D26A variants, while the D12A and D13A variants predominantly produced a triply dehydrated product. These results confirmed that Asp22, Asp24, and Asp26 are the acceptor residues. Ala variants were similarly generated for the potential donor residues (Ser8, Ser9, Thr11, Thr23, and Ser25) within the MirA core and analyzed as before. While three dehydrations were observed for the T23A and S25A MirA variants, only two dehydrations were observed at most for the S8A, S9A, and T11A MirA variants, implicating the latter as donor residues (Figure S25).

To fully determine the ring connectivity and the conformation of Pro residues in rosaritide_trunc_, we performed multiple two-dimensional NMR experiments. ^1^H–^1^H COSY, ^1^H–^1^H TOCSY, and ^1^H–^13^C HSQC spectra were used to assign 21 out of the 24 spin systems (Figures S26–S29, Table S5). The connectivity between the assembled amino acid was established using ^1^H–^1^H NOESY correlations, with H_α,β_(*i*) → HN­(*i* + 1) signals being utilized to assign the primary linear sequence (Figure S27). The NOE couplings between Asp24_βHa+b_ and Thr11_βH_ confirmed the macrolactone linkage predicted by the Ala-substitution experiments, while those between Asp26_βHa+b_ and Ser9_βH_ allowed us to confirm the identity of the second macrolactone (Figure 3, S28). Despite our initial unsuccessful efforts to retrieve NOESY correlations for the expected third linkage, the recollection of 2D-NOESY with a longer mixing time of 500 ms showed weak NOE correlations between Ser8_βH_ and Asp22_βHa+b_, providing evidence for the final macrolactone (Figures 3 and S28). Moreover, the presence of NOESY cross peaks between Ile15_αH_ and Pro16_δHa+b_ and between Arg20_αH_ and Pro21_δHa+b_ indicated that Pro16 and Pro21 were in the *trans* conformation (Figures S29). As *M. rosaria* is a soil dwelling microbe, rosaritide_trunc_ was then tested for growth inhibitor activity against a brief but diverse panel of bacteria (i.e., *E. coli*, *Bacillus subtilis*, *Sorangium cellulosum*, and *Micrococcus luteus*) using an agar disk diffusion assay. However, no significant growth inhibition or morphological activity was observed (Figure S30).

### Rosaritide Biosynthesis Requires the MirC Partner Protein

While *cis*/*trans* Pro isomerization has been observed previously in graspetides,^6^ a graspetide BGC containing a dedicated peptidylproline isomerase has not been characterized so far. To probe the role of the predicted PpiC-type peptidylprolyl isomerase, MirC, in rosaritide biosynthesis, we generated constructs of MirA alone and in various combinations with MirB and MirC (Figure 4). Interestingly, coexpression of MirA with MirB did not induce any observable mass shift although the ATP-binding residues and the “DFR” motif critical for graspetide synthetase activity are conserved in MirB^7^ (Figure S31). This suggests that unlike other characterized graspetide biosynthetic pathways, the graspetide synthetase alone is insufficient to install linkages on MirA. Coexpression of MirA with MirC also failed to induce a mass shift in MirA; however, coexpression of MirA, MirB, and MirC yielded a mass loss of up to 54 Da, consistent with three graspetide linkages (Figure 4). Thus, rosaritide biosynthesis requires both the graspetide synthetase (MirB) and the putative PpiC-type peptidylprolyl isomerase (MirC), a feature not observed among any characterized graspetides.

To explore the role of MirC in rosaritide biosynthesis, we first used a bioinformatics-based approach. HHPred^33,34^ and AlphaFold^35^ predictions of MirC exhibit an unknown N-terminal domain (N-domain; residues 1–90) along with a SurA-like C-terminal domain (C-domain; residues 105–315) (Figure S32). SurA is a periplasmic chaperone protein involved in the maturation of outer membrane proteins in *E. coli* and comprises a chaperone domain and two parvulin-type PPIase domains. Interestingly, *Micromonospora* lack a periplasm, highlighting the different biological roles of the proteins.^36^ FoldSeek^37^ searches using the AlphaFold predicted structure of MirC as the query returned Cj1289, a SurA-like periplasmic protein from *Campylobacter jejuni* (PDB: 3RGC), as the closest characterized structural homologue. Cj1289, in contrast to SurA, comprises only two structural domains—a SurA-like chaperone domain and a parvulin-type PPIase domain.^38^ Structural alignments with Cj1289 showed that the C-domain of MirC does not share any similarity to the PPIase domain of Cj1289 (Figure S32). However, domains formed by MirC residues 105–214 and 289–315 share structural similarities with the chaperone domain of Cj1289. We also observed that the N-domain of MirC bears weak resemblance to a RiPP recognition element (RRE), which is used in many RiPP biosynthetic pathways to aid in substrate recognition.^5^

To probe whether both domains of MirC are essential for rosaritide biosynthesis, we generated constructs containing MBP-MirA and MirB alongside MirC variants lacking either the N- or C-domain. Both constructs failed to produce any observable modifications on MirA, indicating their necessity for rosaritide biosynthesis (Figure S33). We then attempted to investigate the role of MirC further by purifying MirB and MirC individually but were unable to obtain soluble enzymes after extensive effort. However, when His_6_-tagged MirB was coexpressed with untagged MirC and purified using nickel nitrilotriacetic acid (NTA) resin, we observed an affinity copurification of both MirB and MirC (Figure S34–S36). We then assayed the production of rosaritide in vitro using these copurified proteins and found that they were catalytically active (Figure S37). Based on our analyses, we hypothesize that MirC-like proteins may have coevolved with the MirB-like graspetide synthetases to serve as a chaperone, forming a complex needed for proper folding, activity, and/or delivering the MirA substrate in the right conformation for macrocyclization.

### Characterization of a Large, 2OG-Associated Graspetide, Corallotide

We next turned to the BGC selected for characterization from graspetide group 15 that originated in *Corallococcus exercitus* (Figure 5). *E. coli*-optimized genes for a His_6_-tagged precursor peptide (CorA, WP_171433377.1) alongside the untagged graspetide synthetase (CorB, WP_171433379.1, TIGR04187), 2OG-Fe­(II) dependent oxygenase (CorC, WP_171433381.1, PF13640), and β-lactamase (CorD, WP_171433383.1, PF00144) were heterologously expressed. CorA was then purified by immobilized metal affinity chromatography (IMAC) and digested with trypsin before MS analysis. Several CorA trypsin fragments containing a 2 Da loss were observed (Figures S38–S39), which we hypothesized resulted from a dehydration (−18 Da) and an oxygenation (+16 Da). By analyzing the various fragments with −2 Da, we narrowed down the modification sites to five areas that contained a “KxxxD” motif (Figure S38).

**Figure 5:**
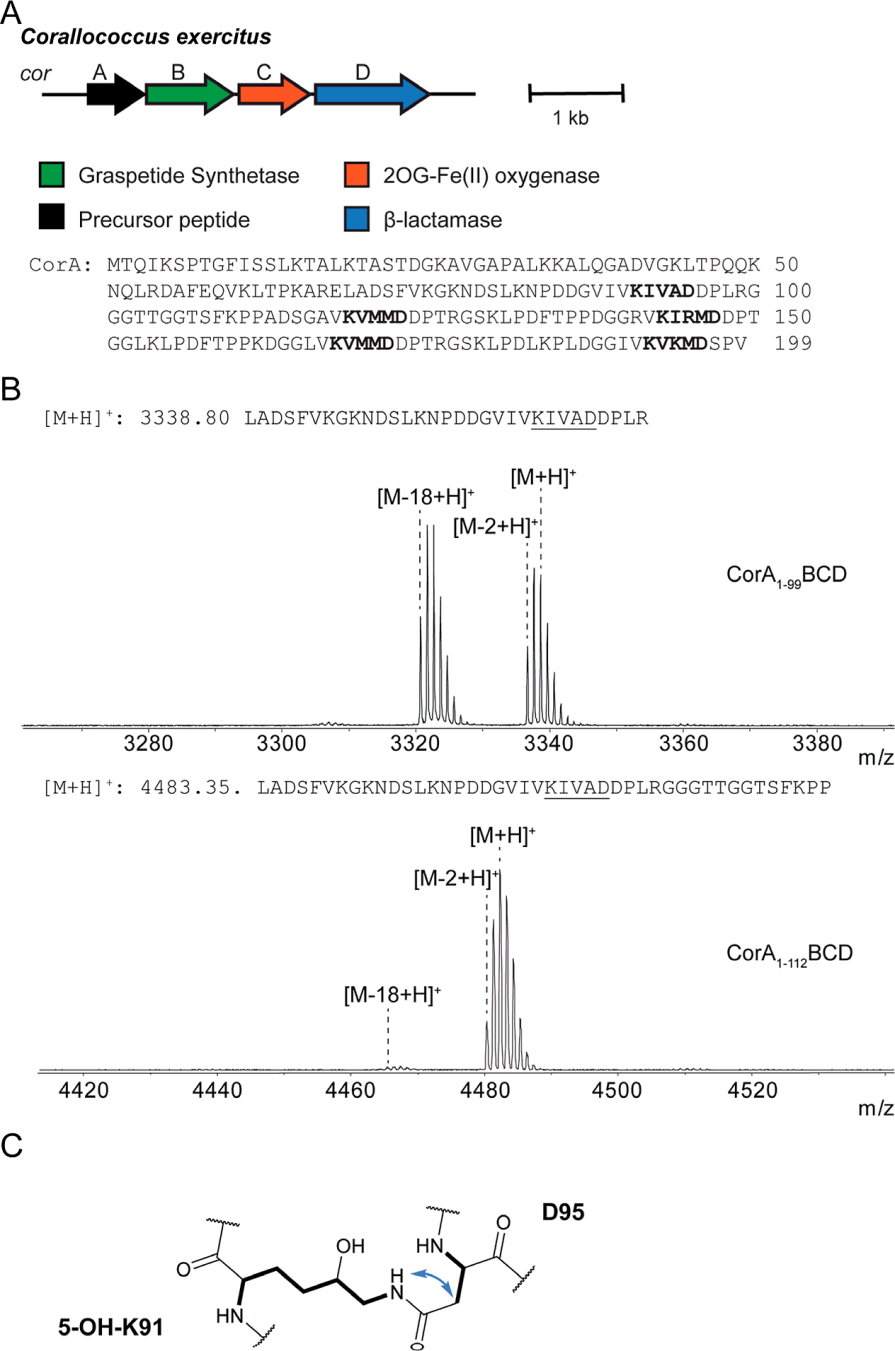
Corallotide BGC, sequence, and modifications. (A) Corallotide BGC from *C*.*exercitus*. The CorA sequence is shown with the modified repeats in bold. (B) GluC fragments of CorA truncates used to localize the sites of modification and minimal substrate. (C) The structure of the 5-hydroxyisopeptide modification installed in Corallotide with NOESY correlations.

As His_6_-CorA had a mass of ∼22 kDa, we next sought to find a minimal substrate containing all of the modifications for more in-depth analysis. We generated CorA truncates near and around the residues of the first modified “KxxxD” motif, which were then expressed with CorB, CorC, and CorD and digested with GluC. While CorA_1–86_ and CorA_1–93_ showed no modifications, CorA_1–99_ contained two products, one with the previously observed loss of 2 Da, and another with a loss of 18 Da (Figures 5 and S40). The CorA_1–112_ variant, however, showed nearly complete conversion to the −2 Da product (Figures 5 and S40). We analyzed the −18 Da CorA_1–99_ product using HRMS/MS, which localized the dehydration to the “KIVAD” motif within the peptide fragment (Figure S41). The −2 Da products from the CorA_1–99_ and CorA_1–112_ variants were also analyzed using HRMS/MS, which localized the modifications to the “KIVAD” motif as well, and yielded the exact mass of the modification as a loss of two hydrogens (Figure S42). The exact masses of the analyzed fragments and the observation of a dehydrated product led us to hypothesize that a macrolactam linkage was first installed by CorB between the single acceptor and donor residue within the “KIVAD” motif, forming an isopeptide bond, followed by the installation of a single hydroxyl group in the modified region by CorC. To test this hypothesis, we generated additional variants of CorA_1–112_, which replaced either the Lys or the Asp in this motif with Ala. These variants produced no modifications, corroborating the formation of a graspetide linkage prior to hydroxylation (Figure S43). The buildup of the dehydrated intermediate suggests that CorC may recognize or bind to the sequence C-terminal to the modified repeat.

To localize the proposed hydroxylation, we purified a trypsin fragment of CorA (NPDDGVIVKIVADDPLR) containing both modifications and elucidated the structure using multidimensional NMR. As with rosaritide, the assignment of the digested modified fragment of CorA was achieved using 2D NMR, including ^1^H–^1^H COSY, ^1^H–^1^H TOCSY, ^1^H–^1^H NOESY, and ^1^H–^13^C HSQC experiments (Figures S44–S47, Table S6). The H_α,β_(i) → HN­(i+1) NOESY correlations were used to assign the linear peptide sequence. Surprisingly, the ^1^H–^1^H TOCSY resonances showed that the Lys9 spin system of the fragment was split into two, such that the backbone amide-K9 coupled with α-γHs, while the side chain amide ζNH-K9 (resulting from the formation of the graspetide linkage) resonated with δ-εHs (Figures 5, S44, and S46). Moreover, the chemical shifts at the δ position of K9 were downshifted (δ_H_ = 3.63 and δ_C_ = 72.21), which assisted in assigning the oxygenation as 5-hydroxylysine on the proposed isopeptide bond.^39^ Eventually, the ^1^H–^1^H NOESY correlations between Lys9_ζNH_ and Asp13_βHa+b_ of the fragment were used to confirm the formation of the isopeptide bond between Lys9 and Asp13 (Figures 5 and S46). The NOESY resonances between Asn1_αH_ and Pro2_δHa+b_, and those between Asp14_αH_ and Pro15_δHa+b_, also showed that both prolyl peptide bonds were in the *trans* conformation (Figure S47). We named the full length, cyclized, and hydroxylated product corallotide, after the native organism. The BGCs in graspetide group 15 were only identified in organisms from the genus *Corallococcus*, a genus of predatory soil bacteria.^40,41^ As such, we performed an agar disk diffusion assay on a small panel of soil bacteria using these compounds to determine if there is any observable biological activity but were unable to observe any significant bioactivity (Figure S30).

To the best of our knowledge, this is the first characterized example of a 5-hydroxyisopeptide bond. Literature searches revealed examples of both 5-hydroxyLys and isopeptide bonds found on certain forms of collagen,^42^ but no hydroxylated isopeptide has been reported. The next closest example to this modification is a 5-hydroxyLys intermediate produced in the alazopeptin biosynthetic pathways^43^ and examples of 5-chloroLys installed by an unrelated group of 2OG-Fe­(II)-dependent halogenases.^44^ Overall, modifications to the Lys δ-carbon appear to be incredibly rare. This example is also the first example of hydroxylation observed on a cyclized graspetide. Another research group recently reported on a pair of oxygenases found within a graspetide BGC from group 18.^30^ These hydroxylases, SmaO and SmaX, perform the β-hydroxylation of aspartate and the conversion of β-hydroxyaspartate to aminomalonic acid, respectively. However, SmaO and SmaX performed modifications at the N-terminus of the linear peptide sequence in the absence of the graspetide synthetase, and it remains unknown whether or how these modifications impact the production of a cyclized graspetide.

### Corallotide Biosynthetic Timing and Requirements

We next investigated the role of each corallotide biosynthetic protein and the order of the modifications. To accomplish this, plasmids individually lacking CorB, CorC, or CorD alongside His_6_-CorA were created and heterologously expressed, followed by purification using Ni-NTA resin, tryptic digestion, and MS analysis of the CorA products (Figures S38 and 39). Unmodified CorA was often cleaved by trypsin at the Lys within each “KxxxD” motif where the modifications are installed. When CorB was omitted, the resulting fragments showed no observable modifications, again supporting the hypothesis that CorC acts only on the cyclized peptide. The omission of CorC from the expression construct led to the accumulation of only the dehydrated product, which was resistant to tryptic digestion, as the Lys was converted to an isopeptide bond. Lastly, when CorD was omitted, both the −18 Da and the −2 Da products were observed. Based on these observations, we hypothesized that the CorD is a nonessential partner protein for CorC that promotes efficient hydroxylation during heterologous expression. These results were confirmed using CorA_1–112_ in place of CorA (Figure 6).

**Figure 6:**
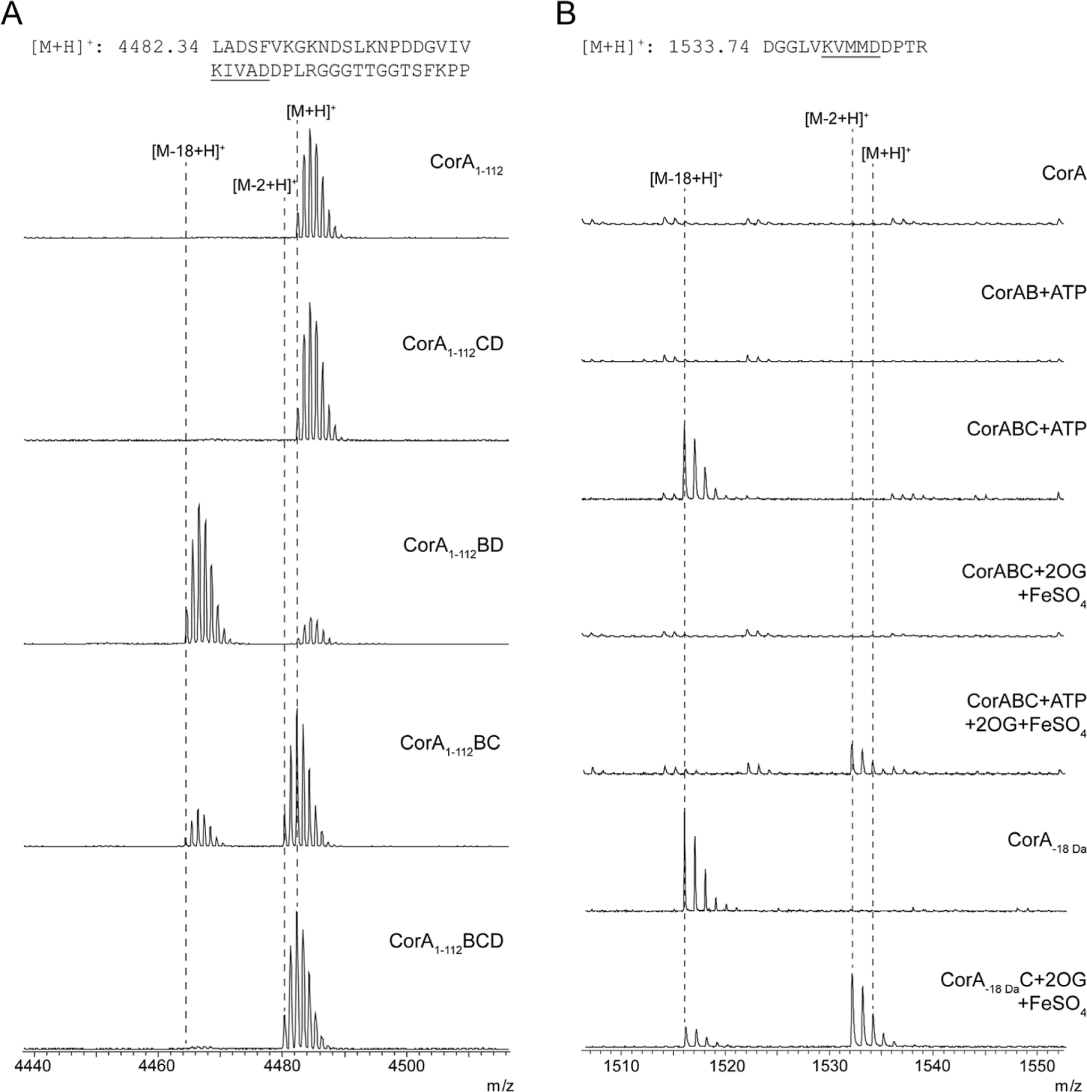
Corallotide biosynthesis. (A) Heterologous coexpressions of CorA_1–112_ with individual genes for Cor biosynthetic proteins being omitted. GluC was used in CorA_1–112_ expressions to keep the unmodified peptides intact. (B) In vitro reconstitution of Cor biosynthetic proteins. Unmodified fragments (e.g., CorA) are cleaved by trypsin and missing from the respective spectra.

We next determined the requirements for each of the Cor biosynthetic proteins by studying them in vitro. While CorC and CorD were robustly expressed and were purified as individual proteins, we initially were unable to purify soluble CorB. Using the strategy that was successful for rosaritide of coexpressing His_6_-CorB with CorC and/or CorD unfortunately also did not yield soluble CorB. Eventually, the use of an MBP tag and coexpression with chaperones permitted access to soluble CorB (Figure S48). However, when purified MBP-CorB was assayed in vitro with CorA and ATP, it showed no activity. Intriguingly, when CorC was also added to the in vitro reactions, we observed dehydration at each “KxxxD” motif (Figures 6 and S49). We found this dependence of CorB on CorC odd, as the heterologous expressions of CorB without CorC were able to produce graspetide linkages. We hypothesized that CorB may be able to install linkages at a severely limited rate without CorC, explaining the observed cyclized product in the heterologous expressions. We tested this by adding a 10-fold excess of CorB to CorA in vitro. As predicted, under these conditions, we observed masses corresponding to the formation of graspetide linkages (Figure S50). We reasoned that this phenomenon may arise either from CorC allosterically activating CorB or from CorC binding the precursor peptide and delivering it to CorB by forming a complex. To test these hypotheses, we performed several in vitro affinity copurification assays with the Cor biosynthetic proteins. First, we immobilized unmodified, N-terminally tagged His_6_-CorA_1–86_, His_6_-CorA_1–112_, and His_6_-CorA onto a Ni-NTA resin. We then treated each with MBP-CorB, MBP-CorC, and MBP-CorD. The immobilized proteins were then washed, eluted, and analyzed using SDS-PAGE (Figure S51). From this analysis, we identified that CorB, CorC, and CorD can bind to the precursor peptide, including the truncated domain with only the N-terminal domain. Having eliminated the CorC substrate-binding hypothesis, we next evaluated whether CorB and CorC formed a complex. We performed another copurification assay that immobilized MBP-CorB on amylose resin and flowed His_6_-CorC with and without CorA and/or CorD. After SDS-PAGE analysis, we found that CorB and CorC were copurified (Figure S52). Combined with the in vitro dependence of the CorB activity on the presence of CorC, we determined that CorC activates CorB.

Next, we reconstituted CorC activity in vitro. As each hydroxylation in corallotide only occurs after the graspetide linkage is formed, we first determined the activity of CorC in reactions containing CorAB and ATP. From this, we found CorC to require both 2-OG and Fe­(II) (as FeSO_4_) to hydroxylate cyclized CorA (Figures 6 and S49). As with the heterologous expressions, we observed no hydroxylations of the proteolyzed fragments in the in vitro reactions of CorA and CorC (or CorC and CorD). We next assessed if CorC could modify previously dehydrated CorA (produced via heterologous expression) in the absence of CorB in vitro and observed efficient hydroxylation (Figures 6 and S49). Intriguingly, these results indicate that while CorB requires CorC for full activity, CorC is stable and active alone. Additionally, although CorD appeared to increase the efficiency of CorC in vivo, we did not observe this effect in vitro when CorD was absent. The results of the affinity copurification assays demonstrate that CorD can bind CorA but is not strongly bound to the CorBC complex. As such, the function of CorD in corallotide biosynthesis remains unknown.

### Evaluation of Corallotide Secondary Structure

Lastly, we investigated the potential effect of the corallotide modifications on the secondary structure of CorA. An AlphaFold3 structure of the full-length precursor peptide predicts the N-terminus of CorA (residues 1–86) to form an alpha helical domain, while the C-terminal section was arranged in an oblong coil (Figure S53). To confirm this, circular dichroism (CD) spectra were collected for CorA samples that were unmodified (CorA, CorACD), or contained dehydrated (CorABD), or dehydrated and oxygenated (CorABCD) products, along with the various CorA_1–112_ products and unmodified CorA_1–86_ (Figure S54). The CD spectra of α helix-rich proteins often have a pair of minima at ∼207 and ∼223 nm. The CorA_1–86_ displayed a CD signal following this pattern. While the CD spectra for the various CorA_1–112_ products closely resembled the CorA_1–86_ spectrum, the CD spectra for the various full-length CorA products showed a strong minimum at ∼205 nm, which seemed unaffected by the various modifications. Interestingly, while the CD spectra of β sheet-rich proteins often contain a single minimum between 210 and 220 nm, certain forms of collagen are known to display a minimum at ∼205 nm.^45–47^ We then attempted to remove the N-terminal alpha helical portion of CorA and its modified products to collect CD spectra of the core sequence alone. However, after proteolysis with GluC to remove the N-terminal domain, the samples rapidly precipitated. Just as the 5-hydroxyisopeptide moiety in corallotide is similar to the modifications observed in collagen, the propensity to aggregate also appears to be another shared property between these two molecules. Other collagen-like peptides have been observed in bacteria that contain the extensive repeating motifs found in eukaryotic collagen (Gly-Xaa-Yaa);^48^ corallotide, however, lacks this characteristic. These observations of sequence divergent, collagen-like peptides in *Corallococcus* present an intriguing line of study for future research in determining the biological role of *cor* BGC.

## Conclusion

In this work, we discovered and investigated the previously unseen partner-protein-dependent biosynthesis of two new graspetides and discovered a novel 5-hydroxyisopeptide moiety on a natural product. We first updated the graspetide RODEO scoring module to identify more diverse graspetide sequences and used it to survey the available genomic data for graspetide BGCs. This resulted in the identification of 19 new graspetide groups, many of which contained co-occurring proteins that may be additional tailoring enzymes capable of functionalizing multimacrocyclic graspetides. We then performed an analysis of protein co-occurrence across all graspetide groups and selected several BGCs for characterization from graspetide groups that strictly co-occurred with certain proteins. We first investigated the *mir* BGC from *M. rosaria* (group 21), including the characterization of a triply cyclized graspetide with a previously unseen ring connectivity, which we named rosaritide. Both the graspetide synthetase MirB and the chaperone-domain-containing partner protein MirC were found to be required for rosaritide biosynthesis. Additionally, while MirB and MirC are unstable alone, when coexpressed, these enzymes copurify with each other in a catalytically active complex. We then turned to the *cor* BGC from *Corallococcus exerticus* (group 15), which produced an unprecedented graspetide with five repeated motifs that each contain a Lys that is first macrocyclized into an isopeptide bond and then hydroxylated at the δ-carbon. This graspetide was named corallotide and was found to have multiple biophysical properties similar to those found in collagen, including the installed modifications, propensity to aggregate, and CD spectral minimum at ∼205 nm. The corallotide biosynthetic timing and requirements were interrogated both in vivo and in vitro, revealing that the graspetide synthetase CorB forms a complex with the divergent 2OG-Fe­(II)-dependent oxygenase CorC. We also determined that while CorB requires CorC for full activity, CorC is active and stable alone. As CorC natively performs late-stage modifications on a macrocyclic peptide, it may be used in future directed evolution campaigns to generate a versatile enzyme for biocatalysis. Overall, this study expands the known chemical space of graspetides and sheds light on more diverse graspetide biosynthetic enzymes and strategies. A unifying theme among the graspetide BGCs studied here is the dependence of the graspetide synthetase on their associated biosynthetic proteins; future studies on new graspetide-tailoring enzymes, especially those with high co-occurrence, may show whether this trend is more widespread.

## Supplementary Material









## Notes

Updated data set of predicted graspetides (XLSX)
NMR data and full spectra (ZIP)
Methods, supporting figures and tables, accession codes, mass spectra, NMR spectra, CD spectra, SDS-PAGE data, enzymatic assay data, and predicted structures (PDF)
Sequences of genes and primers used in this study (XLSX)
