# Supplementary figures and images for: Discovery of Partner Protein-Dependent Graspetide Biosynthesis
Source: ACS Chem Biol. 2026 Mar 14;21(4):698–709. doi: 10.1021/acschembio.5c00957 (PMC13097080; doi:10.1021/acschembio.5c00957)

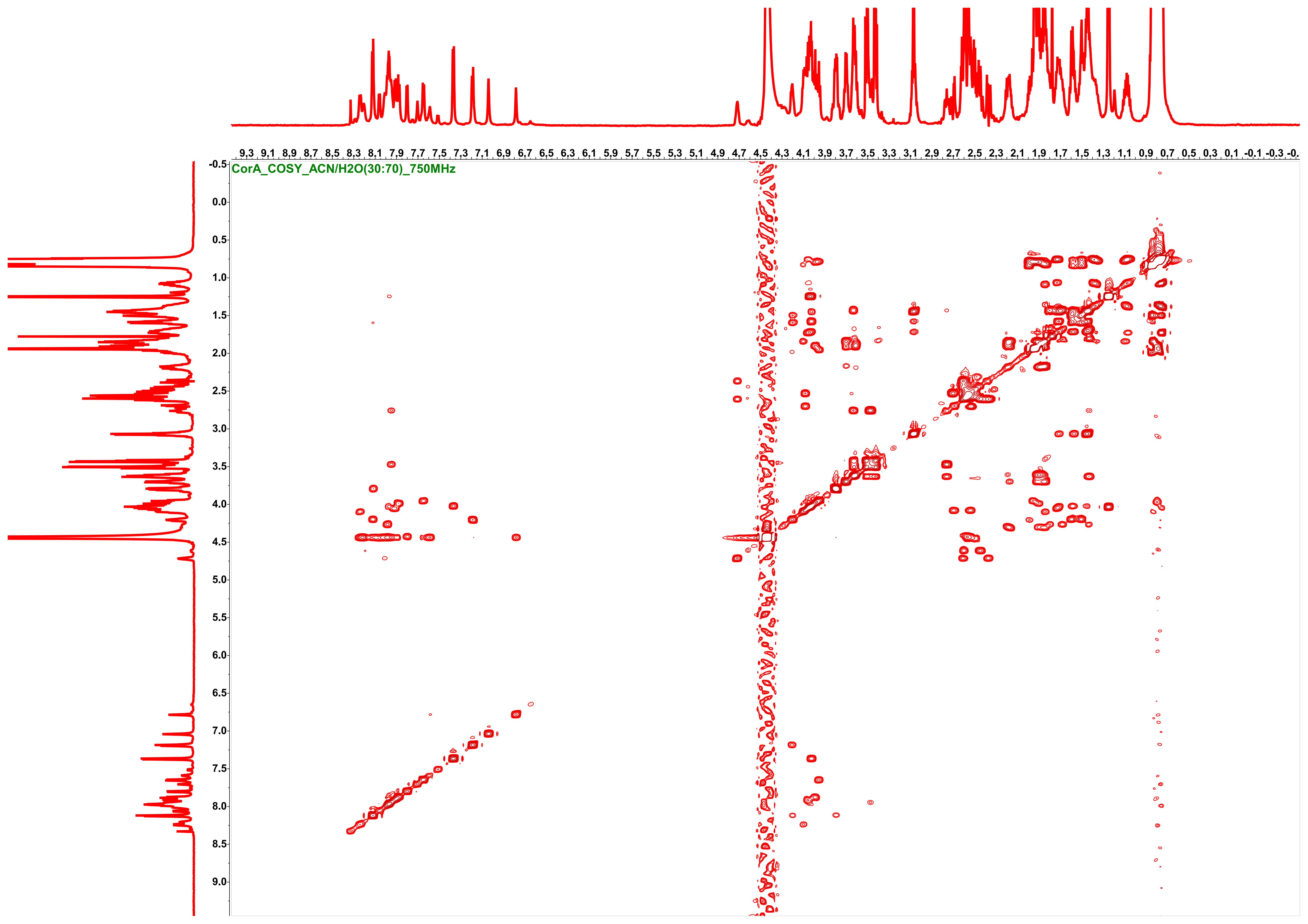

Supplement: Supplementary file 2 [file cb5c00957_si_002.zip › Supp_data_3_NMR/Corallotide_full_spectra_PDF/250721_CorA_30ACN-d3_70H2O_COSY_750 MHz.pdf]

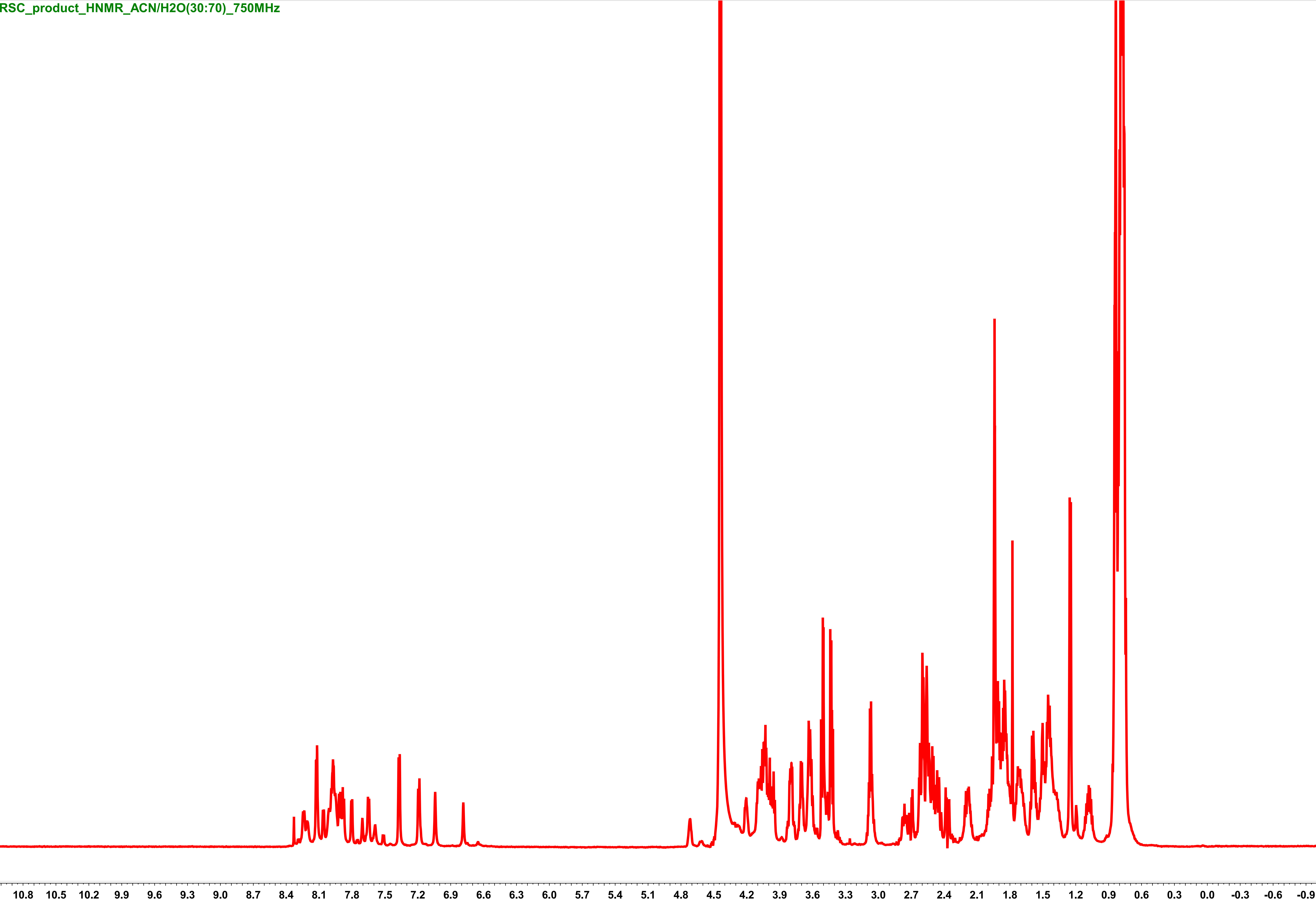

Supplement: Supplementary file 2 [file cb5c00957_si_002.zip › Supp_data_3_NMR/Corallotide_full_spectra_PDF/250721_CorA_30ACN-d3_70H2O_H_750 MHz.pdf]

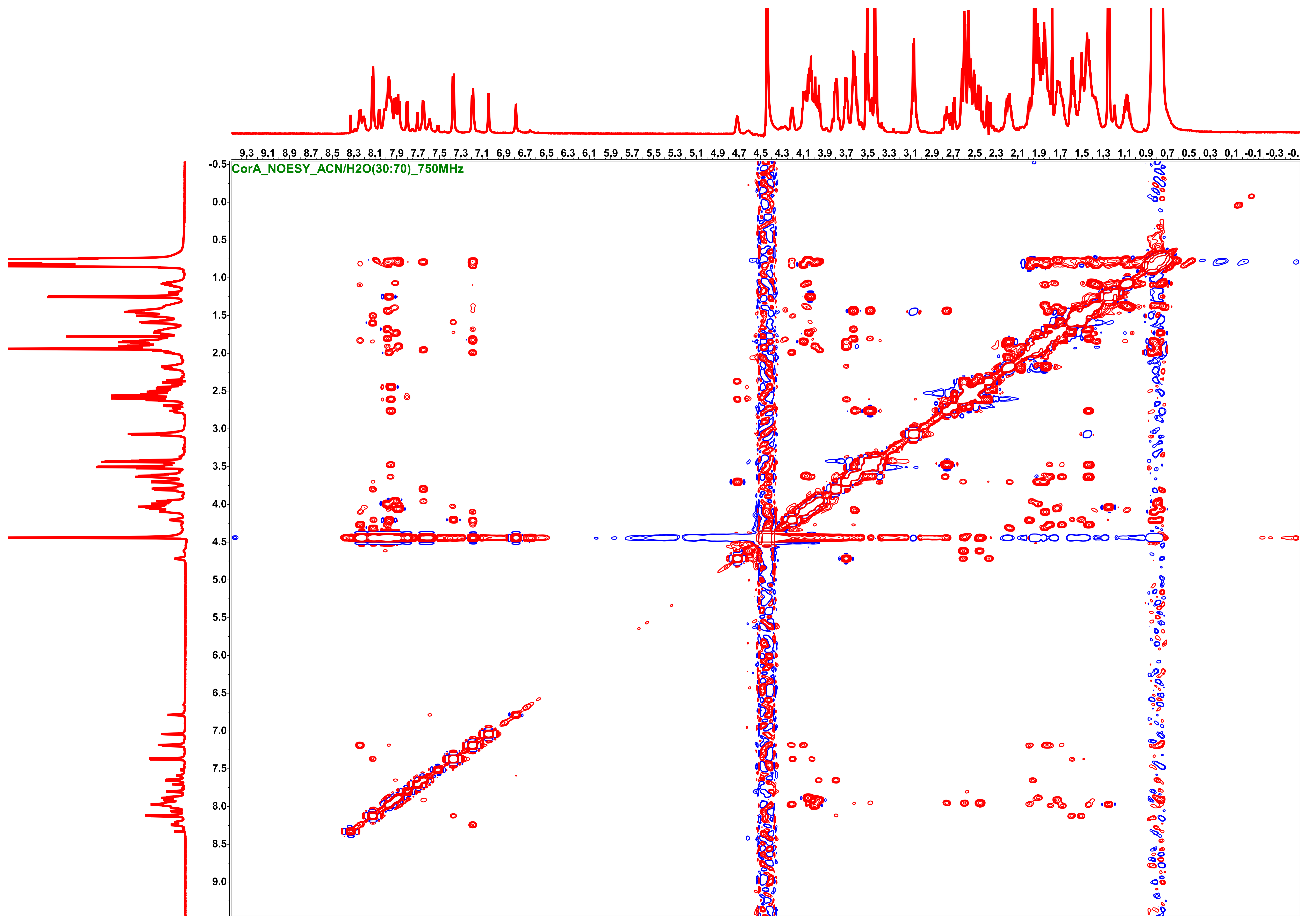

Supplement: Supplementary file 2 [file cb5c00957_si_002.zip › Supp_data_3_NMR/Corallotide_full_spectra_PDF/250721_CorA_30ACN-d3_70H2O_NOESY_750 MHz.pdf]

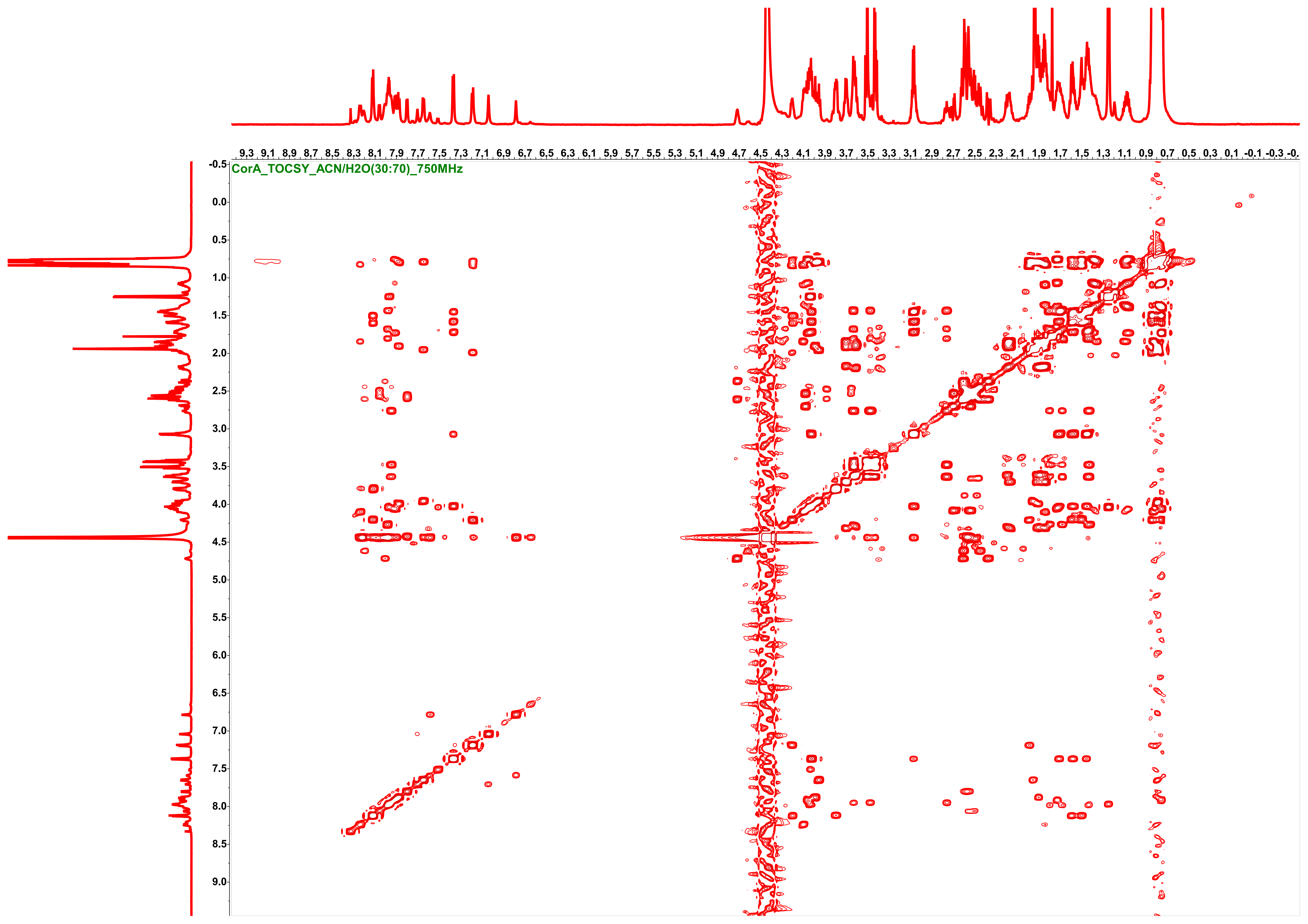

Supplement: Supplementary file 2 [file cb5c00957_si_002.zip › Supp_data_3_NMR/Corallotide_full_spectra_PDF/250721_CorA_30ACN-d3_70H2O_TOCSY_750 MHz.pdf]

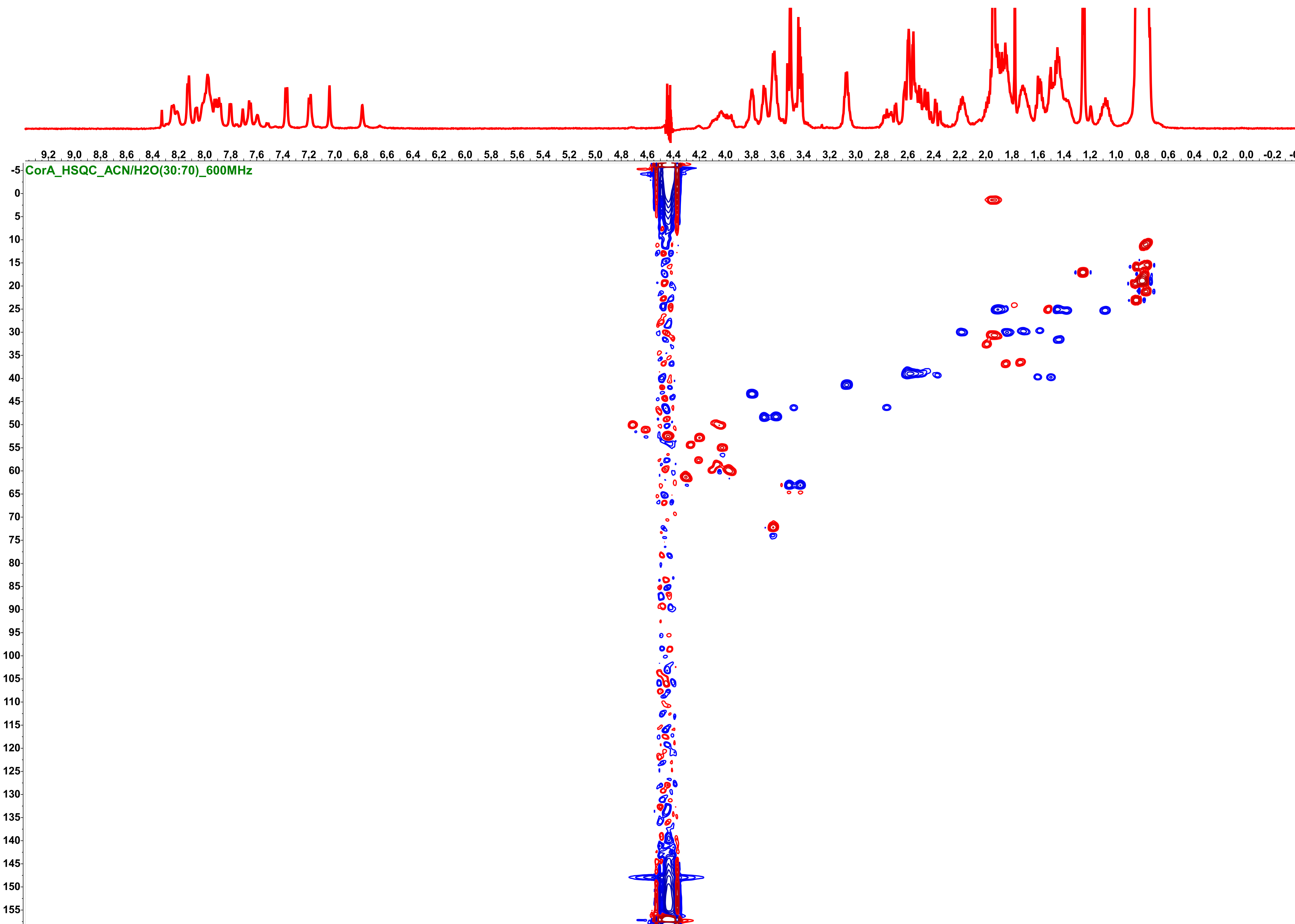

Supplement: Supplementary file 2 [file cb5c00957_si_002.zip › Supp_data_3_NMR/Corallotide_full_spectra_PDF/250721_CorA-30ACN-d3-70H2O_HSQC_600 MHz.pdf]
