# Supplementary material for: Discovery of Partner Protein-Dependent Graspetide Biosynthesis
Source: ACS Chem Biol. 2026 Mar 14;21(4):698–709. doi: 10.1021/acschembio.5c00957 (PMC13097080; doi:10.1021/acschembio.5c00957)
Supplement: Supplementary file 3 [file cb5c00957_si_003.pdf]

Supporting information for

Discovery of Partner Protein-Dependent Graspetide Biosynthesis

Riley S. Carter[1], Sangeetha Ramesh[2], Hamada Saad[3], Abdullah Mubarik[4], and Douglas A. Mitchell[3,5*]

[1]Department of Chemistry, University of Illinois at Urbana-Champaign, Urbana IL 61801, USA

[2]Department of Microbiology, University of Illinois at Urbana-Champaign, 600 South Mathews Avenue, Urbana, Illinois 61801, USA.

[3]Department of Biochemistry, Vanderbilt University School of Medicine, Nashville TN 37232, USA

[4]School of Molecular and Cellular Biology, University of Illinois at Urbana-Champaign, Urbana IL 61801, USA

[5]Department of Chemistry, Vanderbilt University, Nashville TN 37232, USA

*Correspondence to douglas.mitchell@vanderbilt.edu

Table of Contents

Methods……………………………………………………………………………......….....……..5

Table S1. Updated RODEO module heuristics………………………………………….……….….11

Table S2. Updated RODEO module SVM features……………………………….………..12

Table S3. Characterized graspetides…………………………………………………………...14

Table S4. Protein co-occurrence by graspetide group ……………………………………….…...16

Table S5. NMR peak assignments for rosaritide$_{trunc}$……………………………..…………..….30

Table S6. NMR peak assignments for corallotide fragment…...……………..……………..……33

Table S7. Optimal conditions for heterologous expression of graspetide BGC and biosynthetic enzymes………………………………………………………………………………..35

Fig. S1. RODEO module accuracy. ………..………..………..………..…………..…………36

Fig. S2. Graspetide group 25 representative BGC and conservation logo………..........................37

Fig. S3. Graspetide group 26 representative BGC and conservation logo……….........................38

Fig. S4. Graspetide group 27 representative BGC and conservation logo……….........................39

Fig. S5. Graspetide group 28 representative BGC and conservation logo……………..................40

Fig. S6. Graspetide group 29 representative BGC and conservation logo………..........................41

Fig. S7. Graspetide group 30 representative BGC and conservation logo………..........................42

Fig. S8. Graspetide group 31 representative BGC and conservation logo………..........................43

Fig. S9. Graspetide group 32 representative BGC and conservation logo…………….................44

Fig. S10. Graspetide group 33 representative BGC and conservation logo…………....................45

Fig. S11. Graspetide group 34 representative BGC and conservation logo…………....................46

Fig. S12. Graspetide group 35 representative BGC and conservation logo……….........................47

Fig. S13. Graspetide group 36 representative BGC and conservation logo……….........................48

Fig. S14. Graspetide group 37 representative BGC and conservation logo……….........................49

Fig. S15. Graspetide group 38 representative BGC and conservation logo……….........................50

Fig. S16. Graspetide group 39 representative BGC and conservation logo……….........................51

Fig. S17. Graspetide group 40 representative BGC and conservation logo……….........................52

Fig. S18. Graspetide group 41 representative BGC and conservation logo……….........................53

Fig. S19. Graspetide group 42 representative BGC and conservation logo……….........................54

Fig. S20. Graspetide group 43 representative BGC and conservation logo……..........................55

Fig. S21. Graspetide BGCs with additional tailoring enzymes. ………..………..………….……56

Fig. S22. Alkaline methanolysis of $rosaritide_{trunc}$…………………………...……….……....59

Fig. S23. HRMS/MS of fully methanolized $rosaritide_{trunc}$…………………………….……..……60

Fig. S24. MALDI-TOF mass spectra of putative acceptor-to-Ala MirA variants……………..……..61

Fig. S25. MALDI-TOF mass spectra of putative donor-to-Ala MirA variants..…...……………...62

Fig. S26. ^{1}H-^{1}H TOCSY correlations of $rosaritide_{trunc}$….……………………………...……63

Fig. S27. ^{1}H-^{1}H NOESY correlations of $rosaritide_{trunc}$………..………………………..……64

Fig. S28. ^{1}H-^{1}H NOESY correlations of $rosaritide_{trunc}$ macrolactone linkages.…………….......…65

Fig. S29. ^{1}H-^{1}H NOESY correlations of Pro residues in $rosaritide_{trunc}$…...……...…………….……66

Fig. S30. $Rosaritide_{trunc}$ & corallotide bioactivity assay……………..…………………...……67

Fig. S31 Sequence alignment of graspetide synthetases……………………………………………68

Fig. S32. MirC structural predictions………….……………………………………………...70

Fig. S33. MALDI-TOF MS of MirA co-expressed with MirB and MirC truncates………...……71

Fig. S34. His_6-MirB and MirC co-purification……………..…………..………...…..………..72

Fig. S35. In-gel trypsin digestion of His_6-MirB …………...……………...………...……..……..73

Fig. S36. In-gel trypsin digestion of MirC…………….....…...……..………….…...……..……..74

Fig. S37. *In vitro* reconstitution of MirBC activity…….…………………….……………………..75

Fig. S38. CorA tryptic fragments coverage……………………………………………………76

Fig. S39. CorA tryptic fragments…………………………………………………...……..…...77

Fig. S40. Heterologous expression of CorA truncates………………..……………………......…81

Fig. S41. HRMS/MS of the dehydrated $CorA_{1\text{-}99}$ product……………………....……..…..….82

Fig. S42. HRMS/MS of the dehydrated and oxygenated $CorA_{1\text{-}112}$ product...………….........…...83

Fig. S43. $CorA_{1\text{-}112}$ acceptor-donor variants……………………………………………………...84

Fig. S44. ^{1}H-^{1}H TOCSY correlations of corallotide fragment……….…………………………....85

Fig. S45. ^{1}H-^{1}H NOESY correlations of corallotide fragment...……………………………………86

Fig. S46. ^{1}H-^{1}H NMR correlations of corallotide modifications ………………...……...………87

Fig. S47. ^{1}H-^{1}H NOESY correlations of Pro residues in corallotide fragment………………..…….88

Fig. S48. SDS-PAGE of Cor biosynthetic enzymes and CorA variants………..………………...…89

Fig. S49. In vitro reconstitution of Cor biosynthetic enzymes……….……………….….….…90
Fig. S50. *In vitro* reactions with 10-fold excess CorB......……………………………………...…95
Fig. S51. *In vitro* affinity assay of immobilized His_6-CorA variants with Cor proteins…………97
Fig. S52. *In vitro* affinity assay of immobilized MBP-CorB with Cor protein……………………98
Fig. S53. AlphaFold predicted structure of CorA. ……….……….…………..……………..….99
Fig. S54. CorA Circular dichroism spectra……….……….……….…………..……….....…..100
References……………………………………………………………………………………101

Methods

Bioinformatic analyses

A list of potential graspetide synthetases was generated by using the graspetide synthetases of characterized graspetides (**Table S3**) individually as BLAST-P queries against the non-redundant NCBI protein database with up to 10,000 accessions retained for each query. Duplicate accessions were removed, and the remaining accessions were submitted to analysis by the updated RODEO graspetide scoring module. An updated graspetide dataset was generated by filtering the RODEO outputs for potential graspetide precursors with a score of 20 or higher.

A sequence similarity networks (SSNs) was generated for the graspetide synthetases with valid precursors identified by the graspetide RODEO module using the Enzyme Function Initiative Enzyme Similarity Tool (EFI-EST) (https://efi.igb.illinois.edu/)[1]. SSN visualization was performed using an organic layout within Cytoscape[2]. Multiple sequence alignments were performed using MUSCLE[3].

Formulas

Formula 1:

$$\text{Precision} = \frac{\text{true positive}}{\text{true positive} + \text{false positive}}$$

Formula 2:

$$\text{Recall} = \frac{\text{true positive}}{\text{true positive} + \text{false negative}}$$

Formula 3:

$$f1\ score = 2 \times \frac{\text{recall} \times \text{precision}}{\text{recall} + \text{precision}}$$

Genes and Primers

E. coli optimized genes for the *mir* and *cor* BGCs were ordered through TWIST bioscience, while primers used to generate constructs were ordered through Integrated DNA Technologies (See supplementary data 2).

Mass Spectrometry

Samples were analyzed for modifications using a Bruker UltrafleXtreme MALDI-TOF/TOF (matrix-assisted laser desorption/ionization time-of-flight) mass spectrometer (MS). Samples were prepared with equal parts sample and MALDI matrix solution (5% w/v Super-DHB, 60% v/v MeCN, 0.1% formic acid).

High resolution (HR) and tandem MS (MS/MS) spectra were collected using a ThermoFisher Scientific Orbitrap Fusion ESI-MS with an Advion TriVersa Nanomate 100. The MS was calibrated and tuned with Pierce LTQ Velos ESI Positive Ion Calibration Solution (ThermoFisher). The samples were diluted 1:1 with an 80% acetonitrile / 1% acetic acid solution and followed by direct infusion. The MS was operated using the following parameters: mass range, 100-2000 m/z; resolution, 120,000; isolation width (MS/MS), 0.5-1 m/z; normalized collision energy (MS/MS), 35; activation q value (MS/MS), 0.4; activation time (MS/MS), 30 ms. Fragmentation was performed using CID at 30% or 50%. Data analysis was conducted using the Qualbrowser application of Xcalibur software (ThermoFisher Scientific) and the interactive peptide spectral anatator (IPSA).

Screening for heterologous expression of graspetide BGCs and biosynthetic proteins

E. coli-optimized genes for N-terminally His_6 or MBP tagged precursor peptides and untagged tailoring proteins for each studied graspetide BGC were synthesized and assembled into expression plasmids. These BGCs were expressed in *E. coli* BL21 (DE3) and screened for enzymatic modifications using different combinations of growth media (LB, TB, M9), induction temperature (18 °C, 37 °C), and induction time (3, 12, 18 h). Expression media for Cor proteins was supplemented with trace metals[4]. Individual colonies were grown separately overnight in 5 mL of LB at 37 °C and combined. The starter cultures were then inoculated (1:100) into 250 mL fresh medium. The cell cultures were grown at 37 °C with shaking at 200 RPM until OD_{600} = 0.6-1.0 and induced with 500 μM isopropyl β-D-1-thiogalactopyranoside (IPTG). The precursor peptides were purified using either nickel-nitrilotriacetic acid (Ni-NTA) or amylose resin (SI methods), then digested using a RiPP leader peptidase ($LahT_{150}$)[5], Tobacco Etch Virus (TEV) protease, or a commercially available protease (trypsin, GluC). The peptides were then analyzed using a Bruker UltrafleXtreme MALDI-TOF/TOF (matrix-assisted laser desorption/ionization time-of-flight) mass spectrometry (MS) to assess the post-translational modification status of the peptides.

Plasmids were cloned for graspetide biosynthetic enzymes, either as individual or pairs of proteins. Screening was performed as above with the additional variable of co-expressing the Takara pGro7 chaperones. Chaperone expression was induced by adding 0.5 mg/mL L-arabinose to the culture media at inoculation. The optimal growth and expression conditions found for each graspetide BGC and biosynthetic proteins are listed in Table S7.

Purification of His_6-tagged proteins using Ni-NTA resin

For each expressed protein 2 g of wet cell pellet were resuspended in 30 mL of Ni-NTA lysis buffer (50 mM Tris pH 7.5, 500 mM NaCl, 2.5% v/v glycerol), mixed with 100 mg lysozyme, and incubated at 4 °C with rocking for 30 min. The cells were lysed by sonicating cell suspension for 1 min at 50% amplitude every 15 min for a total of 3 min of sonication. The cell lysate was clarified by centrifugation at 17,000 × g for 45 min at 4 °C. Cell lysate was run over 1 mL of Ni-NTA resin preequilibrated with 10 mL of Ni-NTA lysis buffer. After the cell lysate was flowed through the resin, the resin was washed with 10 mL of Ni-NTA lysis buffer, followed by 10 mL of Ni-NTA wash buffer (50 mM Tris pH 7.5, 1 M NaCl, 30 mM imidazole, 2.5% v/v glycerol). Proteins were eluted using 5 mL of Ni-NTA elution buffer (50 mM Tris pH 7.5, 1 M NaCl, 250 mM imidazole, 2.5% v/v glycerol). This eluent was concentrated, and buffer exchanged using Amicon ultra centrifugal filters with either a 3 or 10 kDa MW cutoff. Purified biosynthetic proteins and peptides were flash frozen in liquid nitrogen and stored at -80 °C in Tris storage buffer (50 mM Tris pH 7.5, 150 mM NaCl, 2.5% v/v glycerol). Protein concentration was quantified using a bicinchoninic acid (BCA) assay and by measuring the A280 absorbance.

Purification of maltose-binding protein (MBP) tagged proteins using amylose resin

2 g of wet cell pellet were resuspended in 30 mL of amylose lysis buffer (50 mM Tris pH 7.5, 500 mM NaCl, 2.5% v/v glycerol, 0.1% Triton X-100), mixed with 100 mg lysozyme, and incubated at 4 °C with rocking for 30 min. The cells were lysed by sonicating cell suspension for 1 min at 50% amplitude every 15 min for a total of 3 min of sonication. The cell lysate was clarified by centrifugation at 17000g for 45 min at 4 °C. Cell lysate was run over 1 mL of amylose resin preequilibrated with 10 mL of amylose lysis buffer. After the cell lysate was flowed through the resin, the resin was washed with 10 mL of amylose lysis buffer, then 10 mL of amylose wash buffer (50 mM Tris pH 7.5, 500 mM NaCl, 2.5% v/v glycerol). Proteins were eluted using 5 mL of amylose elution buffer (50 mM Tris pH 7.5, 150 mM NaCl, 10 mM maltose, 2.5% v/v glycerol). This eluent was concentrated and buffer exchanged using Amicon Ultra centrifugal filters with either a 3 KDa or 10 KDa MW cutoff. Purified biosynthetic proteins were flash frozen in liquid nitrogen and stored at -80 °C in Tris storage buffer (50 mM Tris pH 7.5, 150 mM NaCl, 2.5% v/v glycerol). Protein concentration was quantified using a BCA assay.

HPLC purification of full length triply modified MirA

MeCN was added to Amicon-filtered modified MirA dissolved in 10 mM aq. NH_4HCO_3 until a final ratio of 80/20 10 mM aq. NH_4HCO_3/MeCN. This solution was subsequently 0.22 μ m syringe filtered (BD technologies) before purification by high-performance liquid chromatography (HPLC) using a Themo Vanquish UHPLC equipped with an Acclaim C18 column (150 × 4.6 mm, 3 μm particle size, 300 Å pore size) at a flow rate of 1 mL/min. Dissolved MirA was injected with a mobile phase of 10 mM aq. NH_4HCO_3/MeCN with a gradient from 20-30% MeCN over 20 min, 30-90% MeCN over 1 min, hold at 90% MeCN for 5 min, followed by 90-20% MeCN over 1 min

and hold at 20% MeCN for 5 min for subsequent queues. Absorbance was monitored at 220 and 280 nm. Fractions containing triply modified MirA were collected and lyophilized for subsequent analyses.

HPLC purification of Rosaritide$_{trunc}$

HPLC-purified, lyophilized triply modified full-length MirA peptide was digested with trypsin overnight in 50 mM Tris-HCl pH 7 at 37C. The digested sample was subjected to filtration using a 10 kDa MWCO Amicon Ultra 15 mL centrifugal filter and the flow-through containing rosaritide$_{trunc}$ was collected. The filtrate was resuspended in 50 mM Tris-HCl pH 7 and further filtered to collect flow-through until no residual rosaritide$_{trunc}$ in the filtrate was identified by MALDI TOF-MS.

MeCN was added to Amicon-filtered rosaritide$_{trunc}$ to a final concentration of 5%. This solution was subsequently 0.22 µm syringe filtered (BD technologies) and purified by HPLC using a Themo Vanquish UHPLC equipped with an Accucore aq. C18 column (150 × 2.1 mm, 2.6 µm particle size, 80 Å pore size) at a flow rate of 1.5 mL/min. Rosaritide$_{trunc}$ was injected with a mobile phase of 10 mM aq. NH_4HCO_3/MeCN with a gradient from 5-20% MeCN over 15 min, 20-90% MeCN over 1 min, hold at 90% MeCN for 2 min, followed by 90-5% MeCN over 1 min and hold at 5% MeCN for 5 min for subsequent queues. Absorbance was monitored at 220 and 280 nm. Fractions containing rosaritide$_{trunc}$ were collected and lyophilized for subsequent analyses.

HPLC purification of CorA fragment

A sample of concentrated, fully modified CexA$_{1\text{-}120}$ was digested with trypsin overnight in 50 mM Tris pH 7.5 at 37 °C. Acetonitrile was added to a final concentration of 10%, and the sample was then filtered using a 0.22 µm membrane. The tryptic fragment containing the dehydration and oxygenation were purified by HPLC using a Themo Vanquish UHPLC equipped with an Accucore aq. C18 column (150 × 2.1 mm, 2.6 µm particle size, 80 Å pore size) at a flow rate of 1.5 mL/min. The mobile phase consisted of 10 mM NH_4HCO_3/MeCN, with a gradient of 10-45% MeCN over 30 min, 45-90% over 5 min, hold at 90% MeCN for 10 min, followed by 90-10% over 5 min and hold at 10% MeCN for 15 min for subsequent queues. Absorbance was monitored at 220 and 280 nm. Fractions containing the modified fragment were collected and lyophilized for subsequent analyses.

NMR

For the nuclear magnetic resonance (NMR) structure elucidation of rosaritide$_{trunc}$, ~1.25 mg of peptide was dissolved in 300 µL of 1:9 D2O and deionized (Sigma-Aldrich, 99.9% atom D) and placed in a 5 mm D2O-matched Shigemi tube (Millipore-Sigma). For the NMR structure elucidation of the modified corallotide fragment, ~1.5 mg of CorA modified fragment was

dissolved in 500 µl of 7:3 deionized H2O and acetonitrile, d3 (Sigma-Aldrich, 99.9% atom D) and was transferred in a 5 mm diameter NMR tube (Millipore-Sigma). For both rosaritide$_{trunc}$ and the modified corallotide fragment, a series of one-dimensional (1D) and two-dimensional (2D) NMR experiments, including 1H NMR, 1H–1H correlation spectroscopy (1H–1H COSY), 1H–1H total correlation spectroscopy (1H–1H TOCSY), 1H–1H nuclear Overhauser enhancement spectroscopy (1H–1H NOESY) were collected on a 750 MHz Agilent VNMRS narrow-bore NMR operating at 749.375 MHz equipped with a triple resonance 1H–13C/15N Varian probe with gradient and pulse shaping capabilities at 297 K. Data was recorded on a console operating VnmrJ 3.2A. Standard Varian Biopack pulse sequences were used for all acquisitions and mixing times for experiments were set to 30 ms for 1H–1H COSY, 70 ms for 1H–1H TOCSY, and 400 ms for 1H–1H NOESY. An additional 1H–1H NOESY with 500 ms experiment was collected for rosaritide$_{trunc}$ to address the missing correlations for the final macrolactone ring. All spectra were collected with 96 increments in the indirect dimension under water suppression enhanced through T1 effects (WET) solvent suppression. The multiplicity-edited 1H–13C HSQC and 1H–1H TOCSY experiments were conducted on a Bruker AVANCE NEO 600 MHz spectrometer with a 5 mm prodigy BBO probe at 297 K using Topspin 4.1.4 software. All spectra were processed in MestReNova 12.0.1 and calibrated to the residual solvent signals (δH/C 1.94/1.39 for CD3CN). Chemical shift assignments based on all 1D and 2D data are shown in Tables S5-6.

Alkaline methanolysis

Methanolysis was performed on lyophilized, triply modified MirA using 250 mM sodium methoxide in methanol (pH > 11) for 10 min prior to quenching with 5 M acetic acid. Methanolized samples were then dried using a speedvac concentrator, digested with 100 ng of trypsin in 50 mM ammonium bicarbonate for 1h, desalted using C18 ZipTips (EMD Millipore) and eluted into 75% aq. MeCN for MS analysis.

^{1}H-^{1}H NOESY methanolized samples were diluted 1:1 in ESI mix and directly infused onto a ThermoFisher Scientific Orbitrap Fusion ESI-MS using an Advion TriVersa Nanomate 100. Instrument calibration was performed using Pierce LTQ Velos ESI Positive Ion Calibration Solution (ThermoFisher). The MS was operated using the following parameters: 100,000 resolution, 1 *m/z* isolation width (MS/MS), 35 normalized collision energy (MS/MS), 0.4 activation q value (MS/MS), and 30 ms activation time (MS/MS). Fragmentation was performed using collision-induced dissociation (CID) at 30% and 70%. Data analysis was conducted using the Qualbrowser tool within the Xcalibur software suite (ThermoFisher Scientific).

In gel digestion

To perform an in gel digestion, 100 µg of purified His_6-MirB was separated by SDS-PAGE. Bands corresponding to calculated molecular weights of MirB and MirC were subjected to in-gel trypsinolysis per established protocols[6].

In vitro conditions and analysis of graspetide biosynthesis

In vitro reactions were performed at 22 °C for 18 hours, then digested using either 4 µg LahT, 4 ug TEV protease, or 0.1 ng trypsin. Reactions were then desalted using C18 ziptips before analysis by MALDI-TOF. Reactions for the *mir* BGC contained (if present) 10 µM MirA, 1 µM MirBC complex, 50 mM Tris pH 7.5, 150 mM NaCl, 5 mM ATP, 10 mM MgCl2, 1 mM TCEP. Reactions for the *cor* BGC contained (if present) 10 µM CorA, 1 µM CorB, 1 µM CorC ,1 µM CorD, 50 mM HEPES pH 8.0, 100 mM NaCl, 20 mM $MgCl_2$, 5 mM ATP, 10 mM 2-oxoglutarate, 20 mM FeSO4, 5 mM TCEP. Reactions with a 10-fold excess of CorB contained 10 µM CorA, 100 µM CorB, 50 mM HEPES pH 8.0, 100 mM NaCl, 20 mM $MgCl_2$, 5 mM ATP, and 1 mM TCEP.

Bioactivity

An agar disk diffusion assay was performed using purified peptides on Hinton Meuller (HM) agar against several bacterial species. Bacteria were first streaked out on HM agar, then single colonies were grown in 5 mL of HM broth overnight at 30°C. Overnight cultures of each bacterium were then used to inoculate (100 µL) agar plates and allowed to dry. Purified graspetides (100 µg) were added to sterile 6 mm diameter paper disks, allowed to dry, and added to the HM agar plates. Sterile water was used as a negative control and 30 µg gentamycin was used as a positive control. These plates were incubated at 30 °C for 18 h before being analyzed for bioactivity.

In vitro affinity co-purification assay

The affinity co-purification assays for the Cor biosynthetic proteins were accomplished by equilibrating 300 µL of a solution containing 50 mM Tris pH 7.5, 150 mM NaCl, 2.5% v/v glycerol and 5 µM of each included proteins at 22 °C for 30 min. Either 250 µL of preconditioned amylose resin or 100 µL of preconditioned Ni-NTA resin was resuspended with 250 µL of a buffer 1 (50 mM Tris pH 7.5, 150 mM NaCl, 2.5% v/v glycerol) and added to each protein solution. These mixtures were incubated with gentle rocking at 22 °C for 30 min. The mixture was then applied to a fritted column, and the flowthrough collected. The resin was washed twice with 500 µL buffer 2 (50 mM Tris pH 7.5, 500 mM NaCl, 2.5% v/v glycerol), with the flowthrough being collected each time. Finally, the bound proteins were eluted using buffer 3 (50 mM Tris pH 7.5, 150 mM NaCl, 10 mM maltose, 2.5% v/v glycerol) for amylose resin or buffer 4 (50 mM Tris pH 7.5, 500 mM NaCl, 250 mM imidazole, 2.5% v/v glycerol) for Ni-NTA resin. The resulting fractions were analyzed using SDS-PAGE.

Circular dichroism of corallotide products and truncations

Circular dichroism spectra were collected using a Chirascan VX CD spectrometer. Spectra were collected from 280 nm to 180 nm at 25 °C with 0.25 seconds per nm. Samples were buffer exchanged into 20 mM sodium phosphate pH 7.5 and measured in a 1 mm pathlength CD cuvette. Measurements were taken in triplicate, averaged, and baselined using 20 mM sodium phosphate pH 7.5 as a blank.

Table S1. Updated RODEO module heuristics. Updated scoring metrics for the graspetide RODEO module. Updated changes include refinement of heuristic scoring, inclusion of newly published graspetide motifs and features, utilizing regular expressions to capture more potential sequence diversity, and a retrained SVM including these features.

	Feature	Weight
Heuristic Scoring	Precursor peptide <300 bases from graspetide synthetase	+1
	Precursor peptide <1100 bases from graspetide synthetase	+1
	Precursor peptide >2000 bases from graspetide synthetase	-1
	Core peptide >8% Asp	+1
	Core peptide >8% Thr	+1
	Core peptide >7% Pro	+1
	Core peptide contains <3 acceptor residues	-1
	Core peptide contains <3 donor residues	-1
	Core peptide contains more donor than acceptor residues	+1
	Core peptide contains more acceptor residues than leader	+1
	Core peptide contains >13% acceptor residues	+1
	Core peptide contains >18% donor residues	+1
	Precursor peptide ends in Asp	+1
	Leader contains PFxL motif	+1
	Precursor peptide on same strand as graspetide synthetase	+2
	Co-occurring graspetide associated ABC transporter	+1
	Co-occurring graspetide associated acetyltransferase	+1
	Co-occurring graspetide associated methyltransferase	+1
	Precursor peptide matches “serine endopeptidase inhibitor” HMM	+5
	Precursor peptide matches RiPP precursor peptide HMM	+5
	Acceptor compression index > donor compression index*	+1
	Leader peptide contains more positive charge than core	+1
	Precursor peptide matches both acceptor and donor regex	+3
+MEME	Leader motif in precursor peptide identified by FIMO	+1
	Core motif in precursor peptide identified by FIMO	+n
+SVM	SVM classifies precursor peptide as a graspetide	+10

*Compression index is defined as: The position of each donor (Ser, Thr, Lys) and accepter (Asp, Glu) in the putative precursor peptide is divided by the length of the precursor peptide. The minimum, maximum, range, average, and standard deviation for donors and acceptors are then separated calculated. These values are then again averaged to give a single value for the C terminal compression of acceptor and donor residues.

Table S2. Updated RODEO module SVM features. The updated SVM contains features from the heuristic scoring and the biophysical properties of different sections of the precursor peptide. Features were identified using known graspetide BGCs.

**Feature**	**Data type**	**Feature**	**Data type**
Precursor peptide <300 bases from graspetide synthetase	Boolean	Co-occurring graspetide associated ABC transporter: PF0005, PF06472, PF00664, PF03412, TIGR03796, TIGR01846, TIGR03797, TIGR00954, TIGR02203, TIGR02204, TIGR03375	Boolean
Precursor peptide <1100 bases from graspetide synthetase	Boolean	Co-occurring graspetide associated acetyltransferase: PF13302, PF00583, PF13523	Boolean
Precursor peptide >2000 bases from graspetide synthetase	Boolean	Co-occurring graspetide associated methyltransferase: TIGR04188, TIGR04364, PF01135, TIGR00080	Boolean
Core peptide >8% Asp	Boolean	Precursor peptide matches “serine endopeptidase inhibitor” HMM: PF12559, TIGR04186	Boolean
Core peptide >8% Thr	Boolean	Precursor peptide matches RiPP precursor peptide HMM: PF14404, PF14406, PF14407, PF14408, PF14409	Boolean
Core peptide >7% Pro	Boolean	RADAR score	Integer
Core peptide contains <3 acceptor residues	Boolean	Precursor peptide ends in Asp	Boolean
Core peptide contains <3 donor residues	Boolean	Leader contains PFxL motif	Boolean
Core peptide contains more donor than acceptor residues	Boolean	Precursor peptide on same strand as graspetide synthetase	Boolean
Core peptide contains more acceptor residues than leader	Boolean	Acceptor compression index > donor compression index*	Boolean
Core peptide contains >13% acceptor residues	Boolean	Leader peptide contains more positive charge than core	Boolean
Core peptide contains >18% donor residues	Boolean	Precursor peptide matches the acceptor REGEX: [DE]X_{0-3}[DE]X_{0-3}[DE]	Boolean
Precursor peptide contains MEME motif (1-14)	Boolean	Precursor peptide matches the donor REGEX: [TSK]X_{0-3}[TSK]X_{0-3}[TSK]	Boolean
Count of each MEME motif in precursor peptide (1-14)	Integer	Precursor peptide matches both acceptor and donor REGEX	Boolean

Total count of motifs in precursor peptide	Integer	Minimum distance between precursor peptide and graspetide synthetase	Integer
Precursor peptide contains no MEME motifs	Boolean	Precursor peptide length	Integer
Estimated charge of first half of precursor peptide	Integer	Number of each amino acid in the first half of precursor	Integer
Estimated charge of second half of precursor peptide	Integer	Number of each amino acid type (aromatic, aliphatic, hydroxyl, basic, acidic, donors, acceptors) in first half of precursor	Integer
Estimated precursor peptide charge	Integer	% of each amino acid in the first half of precursor	Float
Total number of charged residues in the first half of the precursor peptide	Integer	% of each amino acid type (aromatic, aliphatic, hydroxyl, basic, acidic, donors, acceptors) in the first half of precursor	Float
Total number of charged residues in the second half of the precursor peptide	Integer	Number of each amino acid in the second half of precursor	Integer
Total number of charged residues in precursor peptide	Integer	Number of each amino acid type (aromatic, aliphatic, hydroxyl, basic, acidic, donors, acceptors) in second half of precursor	Integer
Number of each amino acid in full length precursor	Integer	% of each amino acid in the second half of precursor	Float
Number of each amino acid type (aromatic, aliphatic, hydroxyl, basic, acidic, donors, acceptors) in full length precursor	Integer	% of each amino acid type (aromatic, aliphatic, hydroxyl, basic, acidic, donors, acceptors) in second half of precursor	Float
% of each amino acid in full-length precursor	Float	Acceptor compression index	Float
% of each amino acid type (aromatic, aliphatic, hydroxyl, basic, acidic, donors, acceptors) in full length precursor	Float	Donor compression index	Float

Table S3. Characterized graspetides. List of characterized graspetides, their graspetide synthetase accession (if available), the native organism, and the graspetide group.

**Graspetide**	**Graspetide synthetase accession**	**Species**	**Group**
Microviridin A	WP_125730166.1, WP_125730167.1	*Microcystis viridis* NIES-102	1
Microviridin B	WP_069474628.1, WP_103111176.1	*Microcystis aeruginosa* NIES-298	1
Microviridin C	WP_069474628.1, WP_103111176.1	*Microcystis aeruginosa* NIES-298	1
Microviridin D	BBD53456.1, BBD53457.1	*Planktothrix agardhii* NIES-204	1
Microviridin E	BBD53456.1, BBD53457.1	*Planktothrix agardhii* NIES-204	1
Microviridin F	BBD53456.1, BBD53457.1	*Planktothrix agardhii* NIES-204	1
Microviridin G	RCJ24327.1, RCJ24328.1	*Nostoc minutum* NIES-26	1
Microviridin H	RCJ24327.1, RCJ24328.1	*Nostoc minutum NIES*-26	1
Microviridin I	-	*Planktothrix agardhii* strain 2 and 18	1
Microviridin J	-	*Microcystis* sp. UWOCC MRC	1
Microviridin K	WP_042156022.1, WP_042156020.1	*Planktothrix agardhii* NIVA-CYA 126/8	1
Microviridin L	WP_012265553.1, WP_012265554.1	*Microcystis aeruginosa* NIES-843	1
Microviridin SD 1634	-	*Microcystis aeruginosa* IL-215	1
Microviridin SD 1652	-	*Microcystis aeruginosa* IL-215	1
Microviridin SD 1684	-	*Microcystis aeruginosa* IL-215	1
Microviridin LH 1667	-	*Microcystis aeruginosa* TAU-IL 376	1
Microviridin 1777	WP_150975421.1, WP_150975420.1	*Microcystis aeruginosa* EAWAG 127a	1
Marinostatin 1-12	BAA81784.1	*Alteromonas* sp B-10-31	1
Chryseoviridin	WP_027385895.1, WP_027385896.1	*Chryseobacterium gregarium* DS19109	1

Grimoviridin	CZF84007.1, CZF84004.1	*Grimontia marina* CETC 8713	1
Plesiocin	WP_006971586.1	*Plesiocystis pacifica*	2
Thuringinin	WP_000849148.1	*Bacillus thuringiensis* sv. *huazhongensis* BGSC 4BD1	3
OEP-4	OJW02008.1	Sphingobacteriales bacterium 44-61	4
OEP-5	GAJ78971.1	*Vibrio* sp. JCM 18905	5
OEP-6	WP_059136627.1	*Chryseobacterium greenlandenses* UMB34	6
Marinomonasin	CUB04732.1	*Marinomonas fungiae*	7
Prunipeptin	WP_019056921.1	*Streptomyces prunicolor*	11
Fuscimiditide	WP_193587235.1	*Thermobifida fusca*	13
Amycolimiditide	WP_211358019.1	*Amycolatopsis cihanbeyliensis* DSM 45679	13
Thatisin	WP_057916645.1	*Lysobacter antibioticus*	16
Gra-3	WP_121868132.1	*Bacillus* sp. S66	7
Gra-4	WP_124608905.1	*Burkholderia seminalis*	Singleton
Gra-5	WP_110429223.1	*Glaciecola* sp. KUL10	2
Gra-7	WP_106184356.1	*Legionella pneumophila*	8
Gra-8	WP_051546700.1	*Lysobacter capsici*	18
Albusimiditide	WP_003948704.1	*Streptomyces albus* J1074	13
Rosaritide	WP_067373627.1	*Micromonospora rosaria* NRRL 3718	21; this study
Corallotide	WP_171433379.1	*Corallococcus exergitus*	15; this study

Table S4. Protein co-occurrence by graspetide group. The protein co-occurrence for each of the 39 graspetide groups. Co-occurrence was defined as PFAM and TIGRFAM profile hidden Markov models (HMMs) matches within eight open reading frames on either side of queried graspetide synthetases. HMMs that co-occurred in each group at least 15% and with five or more members are recorded below. Co-occurrence values >100% signifies that multiple examples are found in some BGCs.

**Group 1 - 1313 BGCs**	**Count**	**%**	**HMM Description**
Graspetide Synthetases	2509	191.1	
PF12559	1979	150.7	Serine endopeptidase inhibitors
PF00501	409	31.2	AMP-binding enzyme
TIGR00954	356	27.1	3a01203: peroxysomal long chain fatty acyl transporter
PF00561	338	25.7	alpha/beta hydrolase fold
PF03740	337	25.7	Pyridoxal phosphate biosynthesis protein PdxJ
PF00924	331	25.2	Mechanosensitive ion channel
TIGR03466	327	24.9	HpnA: hopanoid-associated sugar epimerase
TIGR01240	325	24.8	mevDPdecarb: diphosphomevalonate decarboxylase
PF04306	270	20.6	Protein of unknown function (DUF456)
PF00903	227	17.3	Glyoxalase/Bleomycin resistance protein/Dioxygenase superfamily
PF12893	210	16.0	Putative lumazine-binding
PF03372	209	15.9	Endonuclease/Exonuclease/phosphatase family
**Group 2 - 801 BGCs**	**Count**	**%**	**HMM Description**
Graspetide Synthetase	826	103.1	
TIGR03796	250	31.2	NHLM_micro_ABC1: NHLM bacteriocin system ABC transporter, peptidase/ATP-binding protein
TIGR01843	222	27.7	type_I_hlyD: type I secretion membrane fusion protein, HlyD family
PF00196	176	22.0	Bacterial regulatory proteins, luxR family
PF13519	137	17.1	von Willebrand factor type A domain
PF00368	129	16.1	Hydroxymethylglutaryl-coenzyme A reductase
TIGR00390	127	15.9	hslU: ATP-dependent protease HslVU, ATPase subunit
**Group 3 - 432 BGCs**	**Count**	**%**	**HMM Description**
Graspetide Synthetase	448	103.7	
PF06412	133	30.8	Conjugal transfer protein TraD
PF08805	107	24.8	PilS N terminal
PF03389	100	23.1	MobA/MobL family
PF00589	99	22.9	Phage integrase family
PF04917	96	22.2	Bacterial shufflon protein, N-terminal constant region

PF01381	87	20.1	Helix-turn-helix
PF13356	74	17.1	Arm DNA-binding domain
**Group 4 - 33 BGCs**	**Count**	**%**	**HMM Description**
Graspetide Synthetase	35	106.1	
PF00239	8	24.2	Resolvase, N terminal domain
PF00665	8	24.2	Integrase core domain
PF00589	6	18.2	Phage integrase family
PF14022	6	18.2	Protein of unknown function (DUF4238)
PF11185	5	15.2	Protein of unknown function (DUF2971)
PF15978	5	15.2	Tn7-like transposition protein D
**Group 5 - 542 BGCs**	**Count**	**%**	**HMM Description**
Graspetide Synthetase	565	104.2	
TIGR03979	84	15.5	His_Ser_Rich: His-Xaa-Ser repeat protein HxsA
TIGR03977	82	15.1	rSAM_pair_HxsC: His-Xaa-Ser system radical SAM maturase HxsC
**Group 6 - 142 BGCs**	**Count**	**%**	**HMM Description**
Graspetide Synthetase	150	105.6	
**Group 7 - 433 BGCs**	**Count**	**%**	**HMM Description**
Graspetide Synthetase	456	105.3	
PF00583	201	46.4	Acetyltransferase (GNAT) family
PF03466	112	25.9	LysR substrate binding domain
TIGR00710	108	24.9	efflux_Bcr_CflA: drug resistance transporter, Bcr/CflA subfamily
PF10554	65	15.0	Ash protein family
**Group 8 - 230 BGCs**	**Count**	**%**	**HMM Description**
Graspetide Synthetase	273	118.7	
TIGR03796	99	43.0	NHLM_micro_ABC1: NHLM bacteriocin system ABC transporter, peptidase/ATP-binding protein
TIGR01843	90	39.1	type_I_hlyD: type I secretion membrane fusion protein, HlyD family
PF13646	81	35.2	HEAT repeats
**Group 9 - 253 BGCs**	**Count**	**%**	**HMM Description**
Graspetide Synthetase	265	104.7	
TIGR02204	174	68.8	MsbA_rel: ABC transporter, permease/ATP-binding protein
PF01972	141	55.7	Serine dehydrogenase proteinase
PF00578	101	39.9	AhpC/TSA family
PF07291	75	29.6	Methylamine utilisation protein MauE

PF01965	61	24.1	DJ-1/PfpI family
PF00144	60	23.7	Beta-lactamase
TIGR02203	56	22.1	MsbA_lipidA: lipid A export permease/ATP-binding protein MsbA
PF11730	54	21.3	Protein of unknown function (DUF3297)
PF04828	43	17.0	Glutathione-dependent formaldehyde-activating enzyme
TIGR02160	43	17.0	PA_CoA_Oxy5: phenylacetate-CoA oxygenase/reductase, PaaK subunit
**Group 10 - 156 BGCs**	**Count**	**%**	**HMM Description**
Grasp	162	103.8	
PF13175	67	42.9	AAA ATPase domain
TIGR01073	63	40.4	pcrA: ATP-dependent DNA helicase PcrA
PF00589	61	39.1	Phage integrase family
PF03432	54	34.6	Relaxase/Mobilisation nuclease domain
PF01668	34	21.8	SmpB protein
PF04355	28	17.9	SmpA / OmlA family
PF03364	27	17.3	Polyketide cyclase / dehydrase and lipid transport
PF03658	25	16.0	RnfH family Ubiquitin
TIGR00634	25	16.0	recN: DNA repair protein RecN
**Group 11 - 3324 BGCs**	**Count**	**%**	**HMM Description**
Graspetide Synthetase	3515	105.7	
PF14404	3505	105.4	Ribosomally synthesized peptide in Streptomyces species
PF06803	1466	44.1	Protein of unknown function (DUF1232)
PF17196	1304	39.2	Protein of unknown function (DUF5133)
PF12833	974	29.3	Helix-turn-helix domain
PF10027	942	28.3	Predicted integral membrane protein (DUF2269)
PF00248	890	26.8	Aldo/keto reductase family
PF01408	881	26.5	Oxidoreductase family, NAD-binding Rossmann fold
PF07228	874	26.3	Stage II sporulation protein E (SpoIIE)
TIGR02203	859	25.8	MsbA_lipidA: lipid A export permease/ATP-binding protein MsbA
PF00941	760	22.9	FAD binding domain in molybdopterin dehydrogenase
TIGR03194	758	22.8	4hydrxCoA_A: 4-hydroxybenzoyl-CoA reductase, alpha subunit
TIGR03198	758	22.8	pucE: xanthine dehydrogenase E subunit
PF04149	618	18.6	Domain of unknown function (DUF397)
PF00144	554	16.7	Beta-lactamase
PF13581	505	15.2	Histidine kinase-like ATPase domain
PF19054	500	15.0	Domain of unknown function (DUF5753)

Group 12 - 279 BGCs	Count	%	HMM Description
Graspetide Synthetase	285	102.2	
TIGR03719	281	100.7	ABC_ABC_ChvD: ATP-binding cassette protein, ChvD family
PF19575	279	100.0	Helix-turn-helix domain
PF00440	242	86.7	Bacterial regulatory proteins, tetR family
PF12802	241	86.4	MarR family
PF07883	237	84.9	Cupin domain
TIGR01341	208	74.6	aconitase_1: aconitate hydratase 1
PF13561	188	67.4	Enoyl-(Acyl carrier protein) reductase
PF20381	187	67.0	Family of unknown function (DUF6676)
TIGR01790	136	48.7	carotene-cycl: lycopene cyclase family protein
PF00877	130	46.6	NlpC/P60 family
PF03176	126	45.2	MMPL family
TIGR02772	122	43.7	Ku_bact: Ku protein
TIGR00051	110	39.4	TIGR00051: acyl-CoA thioester hydrolase, YbgC/YbaW family
PF00583	83	29.7	Acetyltransferase (GNAT) family
PF03061	78	28.0	Thioesterase superfamily
PF13279	43	15.4	Thioesterase-like superfamily
**Group 13 - 5765 BGCs**	**Count**	**%**	**HMM Description**
Graspetide Synthetase	6146	106.6	
TIGR04188	5833	101.2	methyltr_grsp: methyltransferase, ATP-grasp peptide maturase system
TIGR04186	4981	86.4	GRASP_targ: putative ATP-grasp target RiPP
PF19054	2214	38.4	Domain of unknown function (DUF5753)
PF04149	1996	34.6	Domain of unknown function (DUF397)
PF13581	1851	32.1	Histidine kinase-like ATPase domain
PF13560	1245	21.6	Helix-turn-helix domain
**Group 14 - 1643 BGCs**	**Count**	**%**	**HMM Description**
Graspetide Synthetase	1753	106.7	
TIGR04193	1657	100.9	SPASM_w_grasp: SPASM domain peptide maturase, grasp-with-spasm system
TIGR03796	581	35.4	NHLM_micro_ABC1: NHLM bacteriocin system ABC transporter, peptidase/ATP-binding protein
TIGR01843	537	32.7	type_I_hlyD: type I secretion membrane fusion protein, HlyD family
TIGR04139	376	22.9	CxxCx5CxxC_targ: putative peptide modification target, TIGR04139 family
TIGR01200	285	17.3	GLPGLI: GLPGLI family protein

Group 15 - 16 BGCs	Count	%	HMM Description
Graspetide Synthetase	19	118.8	
PF00144	18	112.5	Beta-lactamase
PF00400	18	112.5	WD domain, G-beta repeat
PF13640	18	112.5	2OG-Fe(II) oxygenase superfamily
PF03069	17	106.3	Acetamidase/Formamidase family
PF10604	17	106.3	Polyketide cyclase / dehydrase and lipid transport
PF00155	16	100.0	Aminotransferase class I and II
PF05139	15	93.8	Erythromycin esterase
PF06127	15	93.8	2-hydroxy-palmitic acid dioxygenase Mpo1-like
PF11373	15	93.8	Protein of unknown function (DUF3175)
PF03423	14	87.5	Carbohydrate binding domain (family 25)
PF20106	11	68.8	Family of unknown function (DUF6496)
PF01638	5	31.3	HxlR-like helix-turn-helix
PF13561	5	31.3	Enoyl-(Acyl carrier protein) reductase
**Group 16 - 107 BGCs**	**Count**	**%**	**HMM Description**
Graspetide Synthetase	117	109.3	
TIGR01843	56	52.3	type_I_hlyD: type I secretion membrane fusion protein, HlyD family
TIGR03796	55	51.4	NHLM_micro_ABC1: NHLM bacteriocin system ABC transporter, peptidase/ATP-binding protein
PF00072	35	32.7	Response regulator receiver domain
PF03797	35	32.7	Autotransporter beta-domain
TIGR01783	34	31.8	TonB-siderophor: TonB-dependent siderophore receptor
PF04235	29	27.1	Protein of unknown function (DUF418)
PF00672	27	25.2	HAMP domain
PF00144	26	24.3	Beta-lactamase
PF11528	26	24.3	Protein of unknown function (DUF3224)
TIGR03561	25	23.4	organ_hyd_perox: peroxiredoxin, Ohr subfamily
PF01047	24	22.4	MarR family
PF03929	24	22.4	PepSY-associated TM region
PF00593	23	21.5	TonB dependent receptor
PF09982	23	21.5	Uncharacterized protein conserved in bacteria (DUF2219)
TIGR00949	23	21.5	2A76: homoserine/Threonine efflux protein
TIGR00431	17	15.9	TruB: tRNA pseudouridine(55) synthase
TIGR00952	17	15.9	S15_bact: ribosomal protein uS15
TIGR03591	17	15.9	polynuc_phos: polyribonucleotide nucleotidyltransferase
**Group 17 - 39 BGCs**	**Count**	**%**	**HMM Description**

Graspetide Synthetase	43	110.3	
TIGR01784	43	110.3	T_den_put_tspse: conserved hypothetical protein
PF02656	14	35.9	Domain of unknown function (DUF202)
TIGR02048	11	28.2	gshA_cyano: putative glutamate--cysteine ligase
TIGR00185	10	25.6	tRNA_yibK_trmL: tRNA (cytidine(34)-2'-O)-methyltransferase
TIGR00595	9	23.1	priA: primosomal protein N'
PF00128	8	20.5	Alpha amylase, catalytic domain
PF01551	8	20.5	Peptidase family M23
PF00027	7	17.9	Cyclic nucleotide-binding domain
PF00459	7	17.9	Inositol monophosphatase family
PF00563	7	17.9	EAL domain
PF04832	7	17.9	SOUL heme-binding protein
TIGR00149	7	17.9	TIGR00149_YjbQ: secondary thiamine-phosphate synthase enzyme
TIGR00417	7	17.9	speE: spermidine synthase
TIGR00661	7	17.9	MJ1255: conserved hypothetical protein
TIGR01317	7	17.9	GOGAT_sm_gam: glutamate synthase, NADH/NADPH, small subunit
TIGR01728	7	17.9	SsuA_fam: ABC transporter, substrate-binding protein, aliphatic sulfonates family
TIGR03137	7	17.9	AhpC: peroxiredoxin
PF00578	6	15.4	AhpC/TSA family
PF01679	6	15.4	Proteolipid membrane potential modulator
PF06967	6	15.4	Mo-dependent nitrogenase C-terminus
TIGR00266	6	15.4	TIGR00266: TIGR00266 family protein
**Group 18 - 51 BGCs**	**Count**	**%**	**HMM Description**
Graspetide Synthetase	58	113.7	
TIGR03796	47	92.2	NHLM_micro_ABC1: NHLM bacteriocin system ABC transporter, peptidase/ATP-binding protein
TIGR01843	46	90.2	type_I_hlyD: type I secretion membrane fusion protein, HlyD family
PF13640	21	41.2	2OG-Fe(II) oxygenase superfamily
PF03466	20	39.2	LysR substrate binding domain
PF20329	12	23.5	Family of unknown function (DUF6624)
PF20327	11	21.6	Family of unknown function (DUF6622)
PF04303	10	19.6	PrpF protein
PF08908	10	19.6	Putative oxygenase MesX
TIGR00711	10	19.6	efflux_EmrB: drug resistance MFS transporter, drug:H+ antiporter-2 (14 Spanner) (DHA2) family
TIGR01728	10	19.6	SsuA_fam: ABC transporter, substrate-binding protein, aliphatic sulfonates family
TIGR03566	10	19.6	FMN_reduc_MsuE: FMN reductase

PF00069	9	17.6	Protein kinase domain
PF02219	9	17.6	Methylenetetrahydrofolate reductase
PF03965	9	17.6	Penicillinase repressor
PF05569	9	17.6	BlaR1 peptidase M56
PF13561	9	17.6	Enoyl-(Acyl carrier protein) reductase
TIGR02999	9	17.6	Sig-70_X6: RNA polymerase sigma factor, TIGR02999 family
PF00089	8	15.7	Trypsin
**Group 19 - 33 BGCs**	**Count**	**%**	**HMM Description**
Graspetide Synthetase	37	112.1	
TIGR02203	30	90.9	MsbA_lipidA: lipid A export permease/ATP-binding protein MsbA
TIGR00001	29	87.9	rpmI_bact: ribosomal protein bL35
TIGR00168	29	87.9	infC: translation initiation factor IF-3
TIGR00631	29	87.9	uvrb: excinuclease ABC subunit B
TIGR01032	29	87.9	rplT_bact: ribosomal protein bL20
PF05567	28	84.8	Neisseria PilC beta-propeller domain
PF16732	28	84.8	Type IV minor pilin ComP, DNA uptake sequence receptor
TIGR00418	28	84.8	thrS: threonine--tRNA ligase
PF14341	27	81.8	PilX N-terminal
PF16074	27	81.8	Type IV Pilus-assembly protein W
TIGR00468	25	75.8	pheS: phenylalanine--tRNA ligase, alpha subunit
TIGR02523	25	75.8	type_IV_pilV: type IV pilus modification protein PilV
TIGR00472	20	60.6	pheT_bact: phenylalanine--tRNA ligase, beta subunit
PF12019	17	51.5	Type II transport protein GspH
PF13517	5	15.2	FG-GAP-like repeat
**Group 20 - 44 BGCs**	**Count**	**%**	**HMM Description**
Graspetide Synthetase	47	106.8	
TIGR01843	42	95.5	type_I_hlyD: type I secretion membrane fusion protein, HlyD family
TIGR03796	40	90.9	NHLM_micro_ABC1: NHLM bacteriocin system ABC transporter, peptidase/ATP-binding protein
PF20329	28	63.6	Family of unknown function (DUF6624)
PF01915	11	25.0	Glycosyl hydrolase family 3 C-terminal domain
PF06824	10	22.7	Metal-independent alpha-mannosidase (GH125)
PF17753	10	22.7	Ig-fold domain
PF00728	9	20.5	Glycosyl hydrolase family 20, catalytic domain
**Group 21 - 325 BGCs**	**Count**	**%**	**HMM Description**
Graspetide Synthetase	346	106.5	

TIGR04500	350	107.7	PpiC_rel_mature: putative peptide maturation system protein
TIGR03796	241	74.2	NHLM_micro_ABC1: NHLM bacteriocin system ABC transporter, peptidase/ATP-binding protein
TIGR04497	210	64.6	GRASP_targ_2: putative ATP-grasp target RiPP
PF03704	70	21.5	Bacterial transcriptional activator domain
PF00072	66	20.3	Response regulator receiver domain
PF07730	57	17.5	Histidine kinase
PF00196	54	16.6	Bacterial regulatory proteins, luxR family
**Group 22 - 9 BGCs**	**Count**	**%**	**HMM Description**
Graspetide Synthetase	9	100.0	
**Group 23 - 59 BGCs**	**Count**	**%**	**HMM Description**
Graspetide Synthetase	59	100.0	
TIGR01843	63	106.8	type_I_hlyD: type I secretion membrane fusion protein, HlyD family
TIGR03796	62	105.1	NHLM_micro_ABC1: NHLM bacteriocin system ABC transporter, peptidase/ATP-binding protein
PF20329	61	103.4	Family of unknown function (DUF6624)
TIGR01407	61	103.4	dinG_rel: putative DnaQ family exonuclease/DinG family helicase
PF00199	60	101.7	Catalase
TIGR00507	56	94.9	aroE: shikimate dehydrogenase
PF13857	49	83.1	Ankyrin repeats (many copies)
PF07676	40	67.8	WD40-like Beta Propeller Repeat
TIGR00742	36	61.0	yjbN: tRNA dihydrouridine synthase A
TIGR01387	31	52.5	cztR_silR_copR: heavy metal response regulator
TIGR01386	23	39.0	cztS_silS_copS: heavy metal sensor kinase
PF12850	22	37.3	Calcineurin-like phosphoesterase superfamily domain
PF13561	22	37.3	Enoyl-(Acyl carrier protein) reductase
TIGR00883	15	25.4	2A0106: MFS transporter, metabolite:H+ symporter (MHS) family protein
PF12796	11	18.6	Ankyrin repeats (3 copies)
**Group 25 - 145 BGCs**	**Count**	**%**	**HMM Description**
Graspetide Synthetase	154	106.2	
PF07228	22	15.2	Stage II sporulation protein E (SpoIIE)
**Group 26 - 123 BGCs**	**Count**	**%**	**HMM Description**
Graspetide Synthetase	126	100.0	
PF05618	61	48.4	Putative ATP-dependant zinc protease

PF04952	34	27.0	Succinylglutamate desuccinylase / Aspartoacylase family
PF00924	23	18.3	Mechanosensitive ion channel
**Group 27 - 122 BGCs**	**Count**	**%**	**HMM Description**
Graspetide Synthetase	131	107.4	
TIGR03796	32	26.2	NHLM_micro_ABC1: NHLM bacteriocin system ABC transporter, peptidase/ATP-binding protein
PF01584	29	23.8	CheW-like domain
TIGR03709	27	22.1	PPK2_rel_1: polyphosphate:nucleotide phosphotransferase, PPK2 family
TIGR01221	24	19.7	rmlC: dTDP-4-dehydrorhamnose 3,5-epimerase
TIGR03436	23	18.9	acidobact_VWFA: VWFA-related Acidobacterial domain
TIGR00813	20	16.4	sss: transporter, solute:sodium symporter (SSS) family
PF00072	19	15.6	Response regulator receiver domain
**Group 28 - 107 BGCs**	**Count**	**%**	**HMM Description**
Graspetide Synthetase	110	102.8	
TIGR02203	19	17.8	MsbA_lipidA: lipid A export permease/ATP-binding protein MsbA
**Group 29 - 75 BGCs**	**Count**	**%**	**HMM Description**
Graspetide Synthetase	80	106.7	
PF13581	50	66.7	Histidine kinase-like ATPase domain
PF19462	39	52.0	Family of unknown function (DUF5999)
PF03372	35	46.7	Endonuclease/Exonuclease/phosphatase family
PF19560	34	45.3	Family of unknown function (DUF6082)
TIGR04186	33	44.0	GRASP_targ: putative ATP-grasp target RiPP
PF01370	32	42.7	NAD dependent epimerase/dehydratase family
PF13560	31	41.3	Helix-turn-helix domain
PF01041	27	36.0	DegT/DnrJ/EryC1/StrS aminotransferase family
TIGR03960	26	34.7	rSAM_fuse_unch: radical SAM family uncharacterized protein
TIGR03992	24	32.0	Arch_glmU: UDP-N-acetylglucosamine diphosphorylase/glucosamine-1-phosphate N-acetyltransferase
TIGR02133	21	28.0	RPI_actino: ribose 5-phosphate isomerase
PF00196	17	22.7	Bacterial regulatory proteins, luxR family
PF00293	17	22.7	NUDIX domain
PF19979	16	21.3	Family of unknown function (DUF6415)
**Group 30 - 65 BGCs**	**Count**	**%**	**HMM Description**

Graspetide Synthetase	71	109.2	
PF01400	64	98.5	Astacin (Peptidase family M12A)
PF09619	52	80.0	Type III secretion system lipoprotein chaperone (YscW)
TIGR02824	11	16.9	quinone_pig3: putative NAD(P)H quinone oxidoreductase, PIG3 family
PF00501	10	15.4	AMP-binding enzyme
PF04865	10	15.4	Baseplate J-like protein
PF13385	10	15.4	Concanavalin A-like lectin/glucanases superfamily
PF13561	10	15.4	Enoyl-(Acyl carrier protein) reductase
**Group 31 - 49 BGCs**	**Count**	**%**	**HMM Description**
Graspetide Synthetase	49	100.0	
PF05099	15	30.6	Tellurite resistance protein TerB
PF08238	10	20.4	Sel1 repeat
PF00069	9	18.4	Protein kinase domain
PF00350	9	18.4	Dynamin family
PF14281	8	16.3	PD-(D/E)XK nuclease superfamily
**Group 32 - 46 BGCs**	**Count**	**%**	**HMM Description**
Graspetide Synthetase	48	104.3	
TIGR03296	10	21.7	M6dom_TIGR03296: M6 family metalloprotease domain
**Group 33 - 44 BGCs**	**Count**	**%**	**HMM Description**
Graspetide Synthetase	46	104.5	
PF01142	34	77.3	tRNA pseudouridine synthase D (TruD)
TIGR00283	33	75.0	arch_pth2: peptidyl-tRNA hydrolase
PF18481	23	52.3	Domain of unknown function (DUF5616)
PF01841	22	50.0	Transglutaminase-like superfamily
TIGR00337	21	47.7	PyrG: CTP synthase
PF03966	19	43.2	Trm112p-like protein
TIGR03335	16	36.4	F390_ftsA: coenzyme F390 synthetase
PF05763	14	31.8	Protein of unknown function (DUF835)
PF06745	13	29.5	KaiC
PF07670	13	29.5	Nucleoside recognition
PF00072	11	25.0	Response regulator receiver domain
PF03190	11	25.0	Protein of unknown function, DUF255
PF18911	10	22.7	PKD domain
TIGR03402	7	15.9	FeS_nifS: cysteine desulfurase NifS
TIGR03419	7	15.9	NifU_clost: FeS cluster assembly scaffold protein NifU
**Group 34 - 39 BGCs**	**Count**	**%**	**HMM Description**

Graspetide Synthetase	41	105.1	
PF00440	11	28.2	Bacterial regulatory proteins, tetR family
PF07690	9	23.1	Major Facilitator Superfamily
PF00183	8	20.5	Hsp90 protein
PF01266	8	20.5	FAD dependent oxidoreductase
PF13649	7	17.9	Methyltransferase domain
PF00270	6	15.4	DEAD/DEAH box helicase
PF00999	6	15.4	Sodium/hydrogen exchanger family
PF01163	6	15.4	RIO1 family
PF02610	6	15.4	L-arabinose isomerase
PF04203	6	15.4	Sortase domain
PF05076	6	15.4	Suppressor of fused protein (SUFU)
PF11239	6	15.4	Protein of unknown function (DUF3040)
PF17132	6	15.4	alpha-L-rhamnosidase
TIGR00891	6	15.4	2A0112: MFS transporter, sialate:H+ symporter (SHS) family
TIGR01481	6	15.4	ccpA: catabolite control protein A
TIGR01950	6	15.4	SoxR: redox-sensitive transcriptional activator SoxR
TIGR04559	6	15.4	SoxH_rel_PQQ_2: quinoprotein relay system zinc metallohydrolase 2
**Group 35 - 34 BGCs**	**Count**	**%**	**HMM Description**
Graspetide Synthetase	34	100.0	
PF00400	19	55.9	WD domain, G-beta repeat
PF00248	16	47.1	Aldo/keto reductase family
**Group 36 - 33 BGCs**	**Count**	**%**	**HMM Description**
Graspetide Synthetase	33	100.0	
TIGR00758	29	87.9	UDG_fam4: uracil-DNA glycosylase, family 4
PF09843	26	78.8	Predicted membrane protein (DUF2070)
PF02475	24	72.7	Met-10+ like-protein
PF09921	24	72.7	Uncharacterized protein conserved in archaea (DUF2153)
PF04266	23	69.7	ASCH domain
TIGR02168	22	66.7	SMC_prok_B: chromosome segregation protein SMC
PF00557	19	57.6	Metallopeptidase family M24
TIGR02236	17	51.5	recomb_radA: DNA repair and recombination protein RadA
PF09339	12	36.4	IclR helix-turn-helix domain
PF13412	9	27.3	Winged helix-turn-helix DNA-binding
PF14279	9	27.3	HNH endonuclease
TIGR02169	7	21.2	SMC_prok_A: chromosome segregation protein SMC

PF12802	5	15.2	MarR family
**Group 37 - 29 BGCs**	**Count**	**%**	**HMM Description**
Graspetide Synthetase	29	100.0	
TIGR04186	19	65.5	GRASP_targ: putative ATP-grasp target RiPP
PF01636	18	62.1	Phosphotransferase enzyme family
TIGR03396	12	41.4	PC_PLC: phospholipase C, phosphocholine-specific
PF10025	10	34.5	Uncharacterized conserved protein (DUF2267)
PF00069	9	31.0	Protein kinase domain
PF13561	8	27.6	Enoyl-(Acyl carrier protein) reductase
PF00171	7	24.1	Aldehyde dehydrogenase family
PF03176	7	24.1	MMPL family
PF07729	7	24.1	FCD domain
TIGR02100	7	24.1	glgX_debranch: glycogen debranching enzyme GlgX
PF07722	6	20.7	Peptidase C26
PF13560	6	20.7	Helix-turn-helix domain
PF20401	6	20.7	Rhomboid-like protein
TIGR04188	6	20.7	methyltr_grsp: methyltransferase, ATP-grasp peptide maturase system
PF00120	5	17.2	Glutamine synthetase, catalytic domain
PF01425	5	17.2	Amidase
PF03457	5	17.2	Helicase associated domain
PF10604	5	17.2	Polyketide cyclase / dehydrase and lipid transport
TIGR00908	5	17.2	2A0305: ethanolamine permease
**Group 38 - 29 BGCs**	**Count**	**%**	**HMM Description**
Graspetide Synthetase	30	103.4	
PF00293	5	17.2	NUDIX domain
**Group 39 - 28 BGCs**	**Count**	**%**	**HMM Description**
Graspetide Synthetase	48	171.4	
RREFam002	26	92.9	Stand-alone RRE protein in a lasso peptide cluster
TIGR04352	23	82.1	HprK_rel_A: HprK-related kinase A
PF14907	19	67.9	Uncharacterised nucleotidyltransferase
PF13471	15	53.6	Transglutaminase-like superfamily
PF04116	14	50.0	Fatty acid hydroxylase
PF00578	12	42.9	AhpC/TSA family
TIGR02203	12	42.9	MsbA_lipidA: lipid A export permease/ATP-binding protein MsbA
TIGR01134	9	32.1	purF: amidophosphoribosyltransferase
PF14597	8	28.6	Metallo-beta-lactamase superfamily
TIGR01736	8	28.6	FGAM_synth_II: phosphoribosylformylglycinamidine synthase II

TIGR01737	7	25.0	FGAM_synth_I: phosphoribosylformylglycinamidine synthase I
PF13424	6	21.4	Tetratricopeptide repeat
PF02700	5	17.9	Phosphoribosylformylglycinamidine (FGAM) synthase
**Group 40 - 22 BGCs**	**Count**	**%**	**HMM Description**
Graspetide Synthetase	22	100.0	
PF14409	45	204.5	Ribosomally synthesized peptide in Herpetosiphon
TIGR03796	20	90.9	NHLM_micro_ABC1: NHLM bacteriocin system ABC transporter, peptidase/ATP-binding protein
TIGR02956	12	54.5	TMAO_torS: TMAO reductase sytem sensor TorS
PF01012	8	36.4	Electron transfer flavoprotein domain
PF03031	5	22.7	NLI interacting factor-like phosphatase
PF13620	5	22.7	Carboxypeptidase regulatory-like domain
TIGR02203	5	22.7	MsbA_lipidA: lipid A export permease/ATP-binding protein MsbA
**Group 41 - 32 BGCs**	**Count**	**%**	**HMM Description**
Graspetide Synthetase	32	100.0	
PF01179	10	31.3	Copper amine oxidase, enzyme domain
PF01872	8	25.0	RibD C-terminal domain
PF00924	6	18.8	Mechanosensitive ion channel
PF04266	6	18.8	ASCH domain
TIGR00768	6	18.8	rimK_fam: alpha-L-glutamate ligase, RimK family
TIGR01235	6	18.8	pyruv_carbox: pyruvate carboxylase
TIGR02712	6	18.8	urea_carbox: urea carboxylase
TIGR03467	6	18.8	HpnE: squalene-associated FAD-dependent desaturase
TIGR03866	6	18.8	PQQ_ABC_repeats: PQQ-dependent catabolism-associated beta-propeller protein
TIGR04025	6	18.8	PPOX_FMN_DR2398: pyridoxamine 5'-phosphate oxidase, FMN-binding family
TIGR04243	6	18.8	nodulat_NodB: chitooligosaccharide deacetylase NodB
TIGR04515	6	18.8	P450_rel_GT_act: P450-derived glycosyltranferase activator
PF03466	5	15.6	LysR substrate binding domain
PF04149	5	15.6	Domain of unknown function (DUF397)
PF07883	5	15.6	Cupin domain
PF13091	5	15.6	PLD-like domain
PF19054	5	15.6	Domain of unknown function (DUF5753)
PF19912	5	15.6	Domain of unknown function (DUF6385)
PF20562	5	15.6	Domain of unknown function (DUF6772)

TIGR00883	5	15.6	2A0106: MFS transporter, metabolite:H+ symporter (MHS) family protein
TIGR01481	5	15.6	ccpA: catabolite control protein A
**Group 42 - 22 BGCs**	**Count**	**%**	**HMM Description**
Graspetide Synthetase	23	104.5	
PF20329	18	81.8	Family of unknown function (DUF6624)
**Group 43 - 16 BGCs**	**Count**	**%**	**HMM Description**
Graspetide Synthetase	18	112.5	
PF01637	11	68.8	ATPase domain predominantly from Archaea

Table S5. NMR peak assignments for rosaritide$_{trunc}$. Residues involved in a graspetide linkage are annotated with an asterisk.

RESIDUE	POSITION	Δ_C	Δ_H, MULT (J IN HZ)
**ASN4**	CO	n.d.	---------
	α	n.d.	n.d.
	β	n.d.	n.d.
	γ	n.d.	---------
	$-NH_2$	---------	6.85, s + 7.54, overlapped
	$-NH_2$	---------	n.d.
**GLY5**	CO	n.d.	---------
	α	n.d.	n.d.
	-NH-	---------	n.d.
**ALA6**	CO	n.d.	---------
	α	50.33, CH	4.07, overlapped
	β	15.91, CH_3	1.23, d (7.7)
	-NH-	---------	8.15, br s
**GLY7**	CO	n.d.	---------
	α	40.xx, CH_2	3.74, overlapped + 3.81, overlapped
	-NH-	---------	8.24, overlapped
**SER8***	CO	n.d.	---------
	α	53.57, CH	4.58, overlapped
	β	65.17, CH_2	4.14, overlapped + 4.49, overlapped
	OH	---------	n.d.
	-NH-	---------	7.51, d (7.2)
**SER9***	CO	n.d.	---------
	α	51.38, CH	4.85, overlapped
	β	65.74, CH_2	3.77, overlapped
	-NH-	---------	8.41, overlapped
**LYS10**	CO	n.d.	---------
	α	n.d.	4.66, m
	β	31.17, CH_2	1.60, m + 1.72, m
	γ	22.18, CH_2	1.26, overlapped +1.37, overlapped
	δ	26.30, CH_2	1.55, overlapped
	ε	39.35, CH_2	2.83, overlapped
	$-NH_2$	---------	n.d.
	-NH-	---------	8.79, d (8.7)
**THR11***	CO	n.d.	---------
	α	n.d.	4.69, m
	β	72.76, CH	5.22, q (6.6)
	γ	16.04, CH_3	0.75, d (6.6)
	-NH-	---------	7.82, d (9.6)
**ILE12**	CO	n.d.	---------
	α	58.19, CH	4.17, overlapped
	β	37.18, CH_2	1.60, m
	γ^1	24.36, CH_2	0.91, overlapped +1.20, overlapped
	γ^2	14.52, CH_3	0.64, overlapped
	δ	9.91, CH_3	0.65, overlapped
	-NH-	---------	8.43, overlapped
**ASP13**	CO	n.d.	---------
	α	50.88, CH	4.53, overlapped
	β	39.08, CH_2	2.52, overlapped
	CO_2H	n.d.	---------
	-NH-	---------	8.43, overlapped
**ASP14**	CO	n.d.	---------
	α	51.37, CH	4.42, m

	β	37.72, CH_2	2.38, dd (5.4, 17.5) + 2.62, dd (7.2, 17.5)
	CO_2H	n.d.	---------
	-NH-	---------	8.31, overlapped
**ILE15**	CO	n.d.	---------
	α	50.29/58.18, CH	4.07, m
	β	35.00, CH_2	1.80, m
	$γ^1$	24.47, CH_2	0.94, m +1.26, m
	$γ^2$	14.45, CH_3	0.73, d (7.3)
	δ	10.00, CH_3	0.68, t (8.1)
	-NH-	---------	7.97, br s
**PRO16**	CO	n.d.	---------
	α	61.94, CH	4.19, m
	β	28.43, CH_2	1.71, m + 2.16, m
	γ	24.90/25.10, CH_2	1.81, m + 1.96, m
	δ	47.90, CH_2	3.50, m + 3.74, m
	=N-	---------	---------
**GLY17**	CO	n.d.	---------
	α	n.d.	n.d.
	-NH-	---------	n.d.
**GLY18**	CO	n.d.	---------
	α	42.56, CH_2	3.72, overlapped + 4.05, overlapped
	-NH-	---------	8.05, t (6.1)
**GLY19**	CO	n.d.	---------
	α	42.56, CH_2	3.77, overlapped + 3.90, overlapped
	-NH-	---------	8.37, overlapped
**ARG20**	CO	n.d.	---------
	α	n.d.	4.65, m
	β	31.17, CH_2	1.61, m + 1.74, m
	γ	24.09, CH_2	1.54, m
	δ	40.67, CH_2	3.10, m
	εNH	---------	7.24
	ζ	n.d.	---------
	η1NH	---------	n.d.
	η1NH_2	---------	n.d.
	-NH-	---------	8.36, overlapped
**PRO21**	CO	n.d.	---------
	α	60.25, CH	4.58, m
	β	29.74, CH_2	1.83, m + 2.25, m
	γ	24.78, CH_2	1.93, m
	δ	48.02, CH_2	3.51, m + 3.72, m
	=N-	---------	---------
**ASP22**	CO	n.d.	---------
	α	48.20, CH	4.82, overlapped
	β	35.78, CH_2	2.72, m + 3.20, m
	CO_2H	n.d.	---------
	-NH-	---------	8.26, overlapped
**THR23**	CO	n.d.	---------
	α	57.57, CH	5.16, m
	β	69.80, CH	4.10, m
	γ	19.00, CH_3	1.09, d (6.6)
	OH	---------	n.d.
	-NH-	---------	8.31, overlapped
**ASP24***	CO	n.d.	---------
	α	47.50, CH	4.77, overlapped
	β	38.97, CH_2	2.51, overlapped + 3.12, overlapped
	γ	n.d.	---------

	-NH-	---------	8.12, d (8.4)
**SER25**	CO	n.d.	---------
	α	55.87, CH	4.49/4.65, overlapped
	β	65.79, CH_2	3.67, overlapped + 3.80, overlapped
	OH	---------	n.d.
	-NH-	---------	8.39, overlapped
**ASP26***	CO	n.d.	---------
	α	51.18, CH	4.28, overlapped
	β	35.78, CH_2	2.55, m + 2.68, m
	γ	n.d.	---------
	-NH-	---------	8.49, br d
**PHE27**	CO	n.d.	---------
	α	56.14, CH	4.24, overlapped
	β	37.46, CH_2	2.79, overlapped + 2.98, dd (14.7, 4.8)
	1	n.d.	---------
	2, 6	129.48, CH	7.09, d (7.8)
	3, 5	128.61, CH	7.22, t (7.5)
	4	126.84, CH	7.16, t (7.5)
	CO_2H	n.d.	n.d.
	-NH-	---------	7.73, d (8.3)

Table S6. NMR peak assignments for corallotide fragment. Residues involved in a graspetide linkage are annotated with an asterisk.

RESIDUE	POSITION	*Δ*C	*Δ*H, MULT (*J* IN HZ)
**ASN1**	CO	n.d.	-------
	α	49.70, CH	4.08
	β	38.60, CH_2	2.53, overlapped +2.70, dd (6, 17)
	γ	n.d.	-------
	NH_2	-------	6.78 or 7.04, br s (*cis*)
			7.59 or 7.70, br s (*trans*)
	NH_2	-------	n.d.
**PRO2**	CO	n.d.	-------
	α	61.55, CH	4.29, overlapped
	β	30.08, CH_2	1.85, overlapped +2.19, overlapped
	γ	25.12, CH_2	1.90, overlapped
	δ	48.28, CH_2	3.61, m
	=N-	-------	-------
**ASP3**	CO	n.d.	-------
	α	52.36, CH	4.44, overlapped
	β	38.80, CH_2	2.49, overlapped +2.57, overlapped
	CO_2H	n.d.	n.d.
	-NH-	-------	8.06, d (6.9)
**ASP4**	CO	n.d.	-------
	α	52.51, CH	4.43, overlapped
	β	38.90, CH_2	2.58, overlapped
	CO_2H	n.d.	-------
	-NH-	-------	7.80, d (6.5)
**GLY5**	CO	n.d.	-------
	α	43.36, CH_2	3.80, m
	-NH-	---------	8.11, overlapped
**VAL6**	CO	n.d.	---------
	α	60.10, CH	3.95, overlapped
	β	30.50, CH	1.95, overlapped
	γ1	17.70, CH_3	0.76, overlapped
	γ2	19.35, CH_3	0.84, overlapped
	-NH-	---------	7.65, d (8)
**ILE7**	CO	n.d.	-------
	α	58.60, CH	4.06, overlapped
	β	36.50, CH	1.73, m
	γ1	25.28, CH_2	1.07, overlapped +1.39, overlapped
	γ2	15.47, CH_3	0.76, overlapped
	δ	10.95, CH_3	0.76, overlapped
	-NH-	-------	7.92, overlapped
**VAL8**	CO	n.d.	---------
	α	59.80, CH	3.99, overlapped
	β	30.75, CH	1.91, overlapped
	γ1+γ2	19.00, 2X CH_3	0.79, overlapped
	-NH-	---------	7.88, d (8.5)
**5-HYLYS9***	CO	n.d.	---------
	α	54.30, CH	4.26, overlapped
	β	30.00, CH_2	1.68, overlapped +1.80, overlapped
	γ	31.61, CH_2	1.43, overlapped
	δ	72.21, CH	3.63, overlapped
	ε	46.30, CH_2	2.76, overlapped +3.48, overlapped
	ζ-NH-	---------	7.95, overlapped
	-NH-	---------	7.98, overlapped

**ILE10**	CO	n.d.	---------
	α	59.82, CH	4.10, overlapped
	β	36.82, CH	1.84, overlapped
	γ1	25.35, CH_2	1.09, overlapped +1.38, overlapped
	γ2	15.84, CH_3	0.84, overlapped
	δ	11.20, CH_3	0.78, overlapped
	-NH-	---------	8.24, d (8.6)
**VAL11**	CO	n.d.	---------
	α	57.71, CH	4.21, overlapped
	β	32.50, CH_2	1.99, overlapped
	γ1+γ2	18.50, 2X CH_3	0.80, overlapped
	-NH-	---------	7.19, d (8.5)
**ALA12**	CO	n.d.	---------
	α	50.20, CH	4.04, overlapped
	β	17.01, CH_3	1.25, d (7.5)
	-NH-	---------	7.96, overlapped
**ASP13***	CO	n.d.	---------
	α	51.10, CH	4.61, overlapped
	β	38.30, CH_2	2.44, overlapped +2.61, overlapped
	γ	n.d.	---------
	-NH-	---------	8.20, br s
**ASP14**	CO	n.d.	---------
	α	50.10, CH	4.71, overlapped
	β	39.20, CH_2	2.37, dd (7.2, 16.70) +2.62, overlapped
	CO_2H	n.d.	n.d.
	-NH-	---------	8.01, overlapped
**PRO15**	CO	n.d.	---------
	α	61.42, CH	4.31, overlapped
	β	29.91, CH_2	1.85, overlapped +2.17, overlapped
	γ	25.06, CH_2	1.91, overlapped
	δ	48.48, CH_2	3.70, m
	=N-	---------	---------
**LEU16**	CO	n.d.	---------
	α	52.80 , CH	4.20, overlapped
	β	39.70 , CH_2	1.50, overlapped +1.60, overlapped
	γ	25.05, CH	1.52, overlapped
	δ1	21.20, CH_3	0.76, overlapped
	δ2	23.10, CH_3	0.84, overlapped
	-NH-	---------	8.12, overlapped
**ARG17**	CO2H	n.d.	n.d.
	α	55.00, CH	4.02, overlapped
	β	29.66, CH_2	1.58, overlapped +1.72, overlapped
	γ	25.05, CH_2	1.45, overlapped
	δ	41.41, CH_2	3.08, m
	εNH	---------	8.52
	ζ	n.d.	---------
	η1NH	---------	n.d.
	η2 NH_2	---------	n.d.
	-NH-	---------	7.37, d (7.7)

Table S7. Optimal conditions for heterologous expression of graspetide BGC and biosynthetic enzymes. See Figs. S35 and S49 for SDS-PAGE images.

**Proteins expressed**	**Growth Media**	**Induction temp (°C)**	**Induction time (h)**	**Co-expression with chaperones**
MBP-MirA, MirBC	TB	18	18	No
His_6-CorA, CorBCD	TB	18	18	No
MBP-MirA	TB	18	18	No
His_6-MirB, MirC	TB	18	18	No
His_6-CorA	TB	18	18	Yes
MBP-CorB	TB	37	3	Yes
His_6-CorC	TB	18	18	No
MBP-CorC	TB	37	3	No
His_6-CorD	TB	18	18	No
MBP-CorD	TB	18	18	No

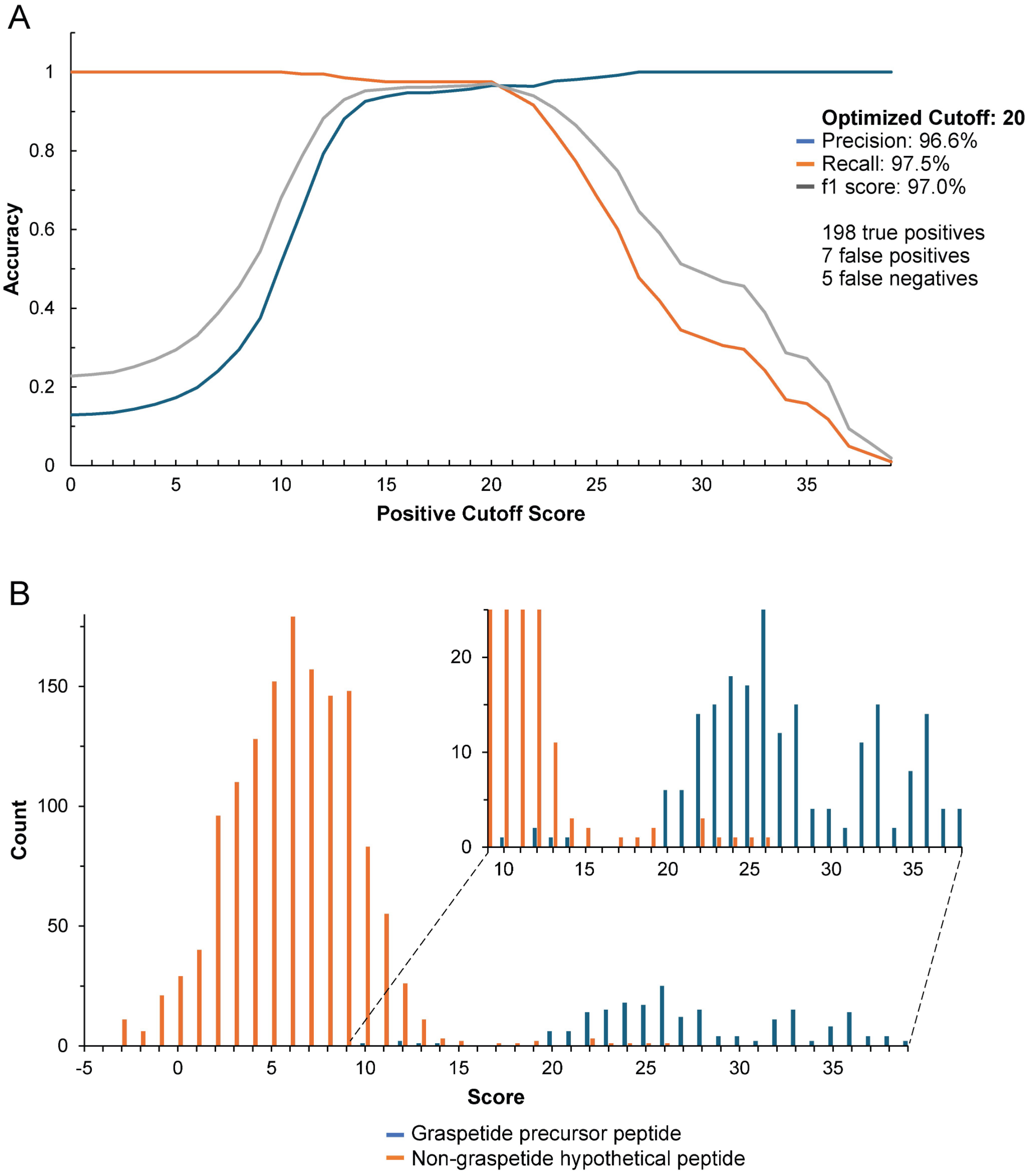

Fig. S1. RODEO module accuracy. The accuracy of the updated graspetide RODEO module calculated using 203 graspetides and the non-graspetide hypothetical protein within each BGC, and several types of non-graspetide RiPPs. A) The precision, recall, and f1 score of the module accuracy was calculated using formulas 1-3. The number with the highest f1 score was used as the cutoff score for the graspetide module to assign a precursor peptide as a valid graspetide. B) The count of the number of peptide sequences at each score cutoff in the testing dataset.

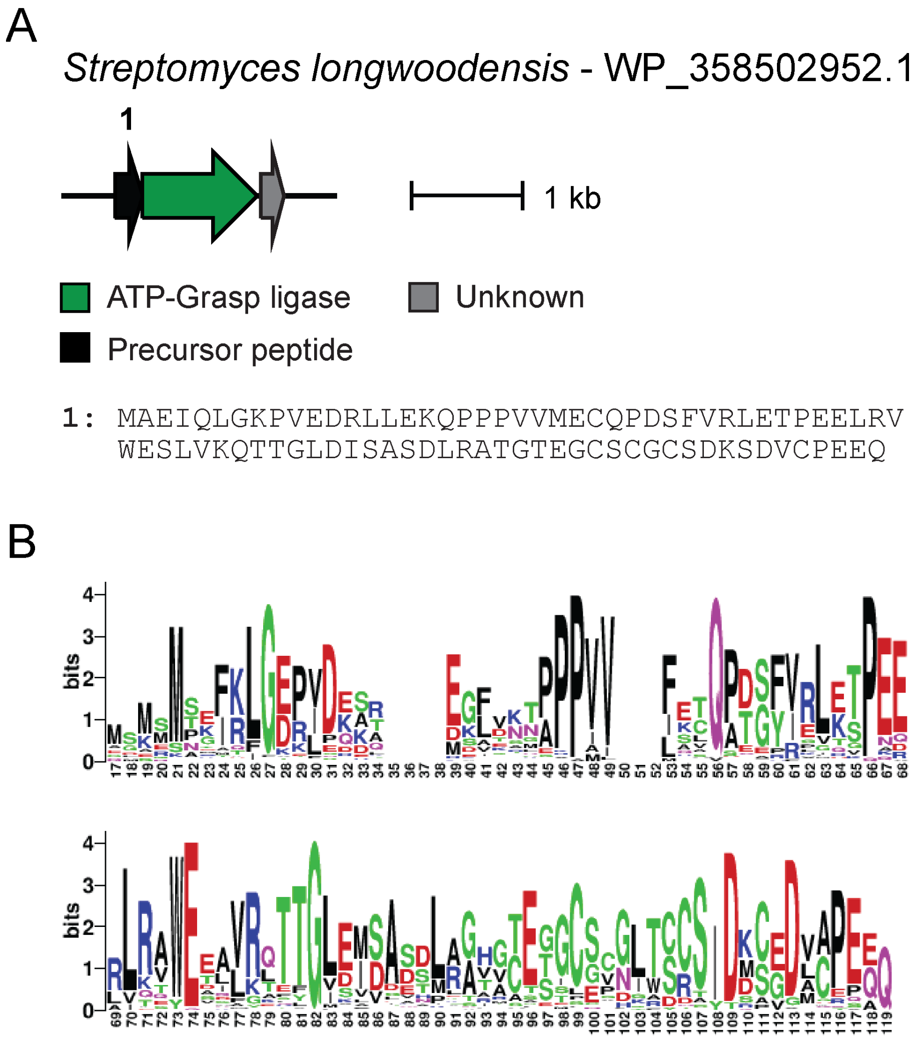

Fig. S2. Graspetide group 25 representative BGC and conservation logo. A) Representative BGC of graspetide group 25 from *Streptomyces longwoodensis*. B) Sequence logo (n = 166) for predicted precursor peptides for graspetide group 25.

A

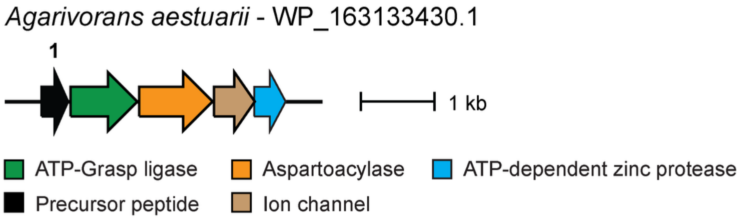

1: MIIDFRFGNKRFYDDKRFKHGFRKSGDFTLLESELLSLYG
ETMRALDAGEMMPEGNQEQRFLLVCSQQLEPETKLERIWM
KYKTLAHSRRRFHTLHSRCKSVTDNFENEDETFVVEDD

B

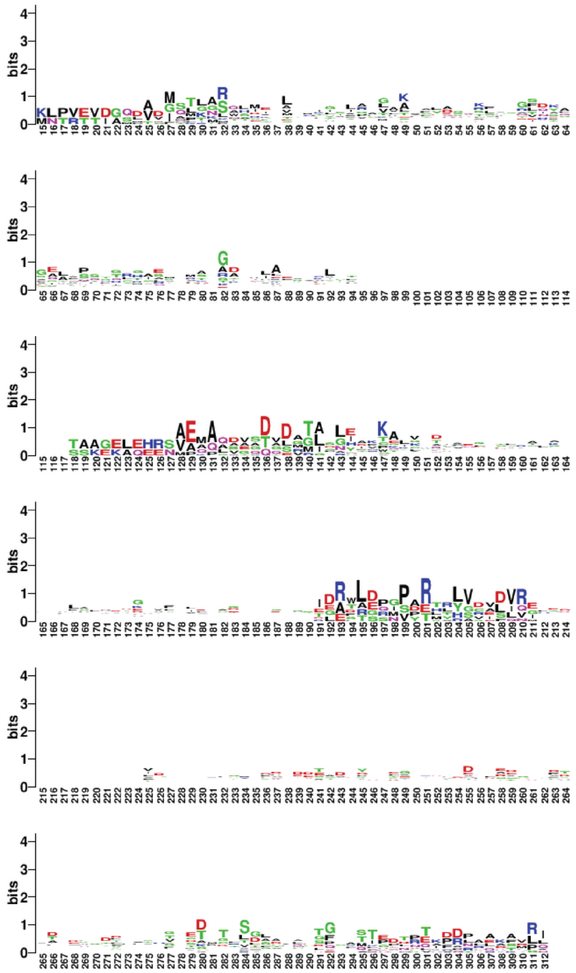

Fig. S3. Graspetide group 26 representative BGC and conservation logo. A) Representative BGC of graspetide group 26 from *Agarivorans aestuarii* containing an ATP-dependent zinc protease, ion channel, and an aspartoacylase. B) Sequence logo (n = 127) for predicted precursor peptides for graspetide group 26. The precursor peptides in this group are more divergent, with fewer conserved residues across the group.

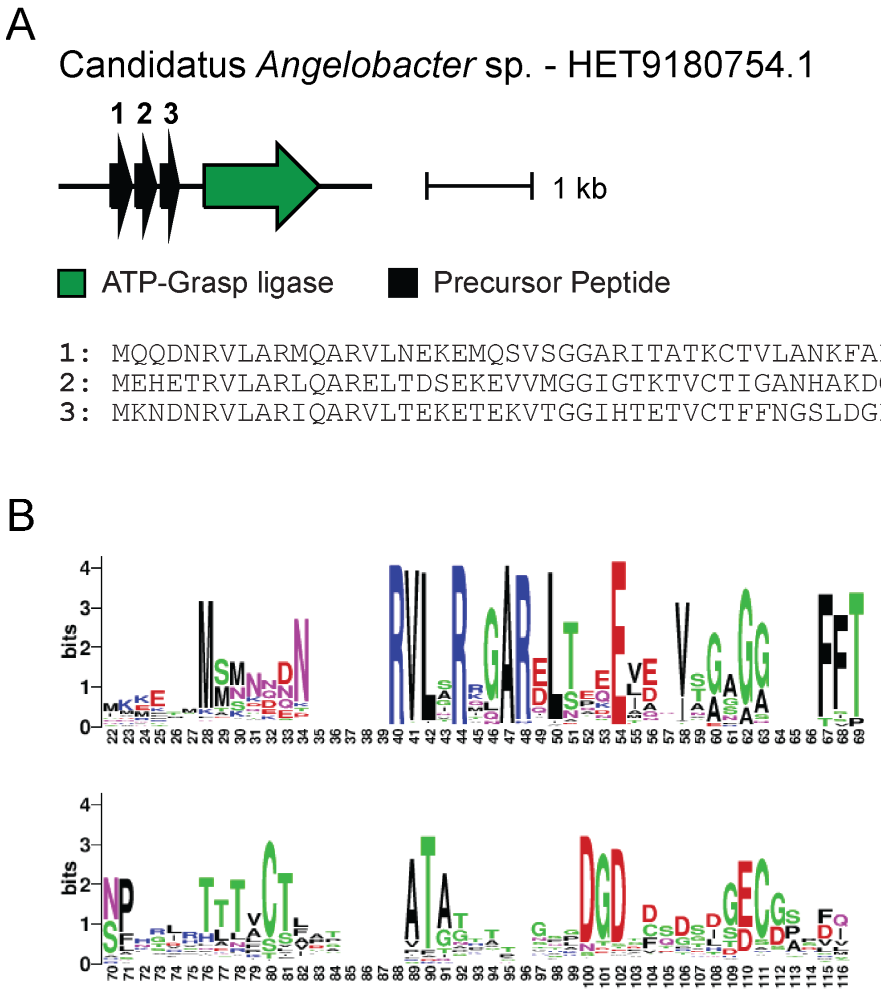

Fig. S4. Graspetide group 27 representative BGC and conservation logo. A) Representative BGC of graspetide group 27 from Candidatus *Angelobacter* sp. with multiple precursor peptides. B) Sequence logo (n = 337) for predicted precursor peptides for graspetide group 27.

A

Thermomonospora amylolytica - WP_119726576.1

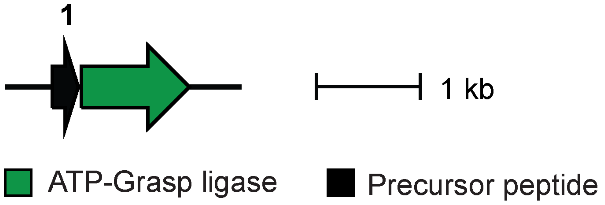


```
1: MTVDTQVRPAIYTGQERMAIAPAPKLAQWERDLIE
   RLELEWDLHQIESMHPDETISGSGDGWDDCDYMP
```

B

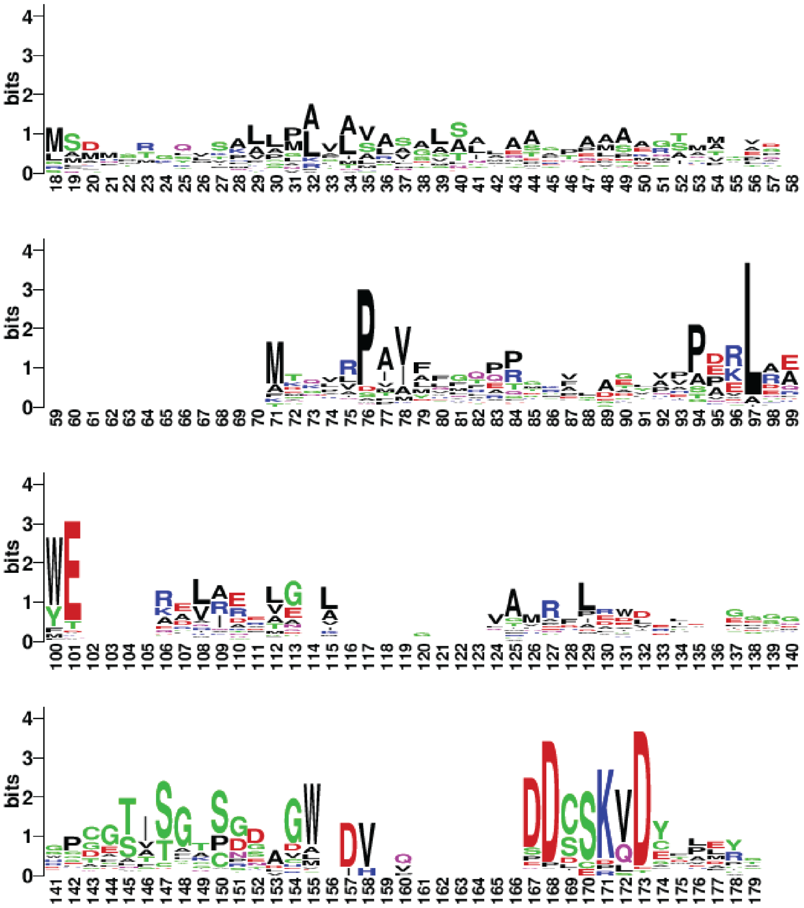

Fig. S5. Graspetide group 28 representative BGC and conservation logo. A) Representative BGC of graspetide group 28 from *Thermomonospora amylolytica*. B) Sequence logo (n = 120) for predicted precursor peptides for graspetide group 28.

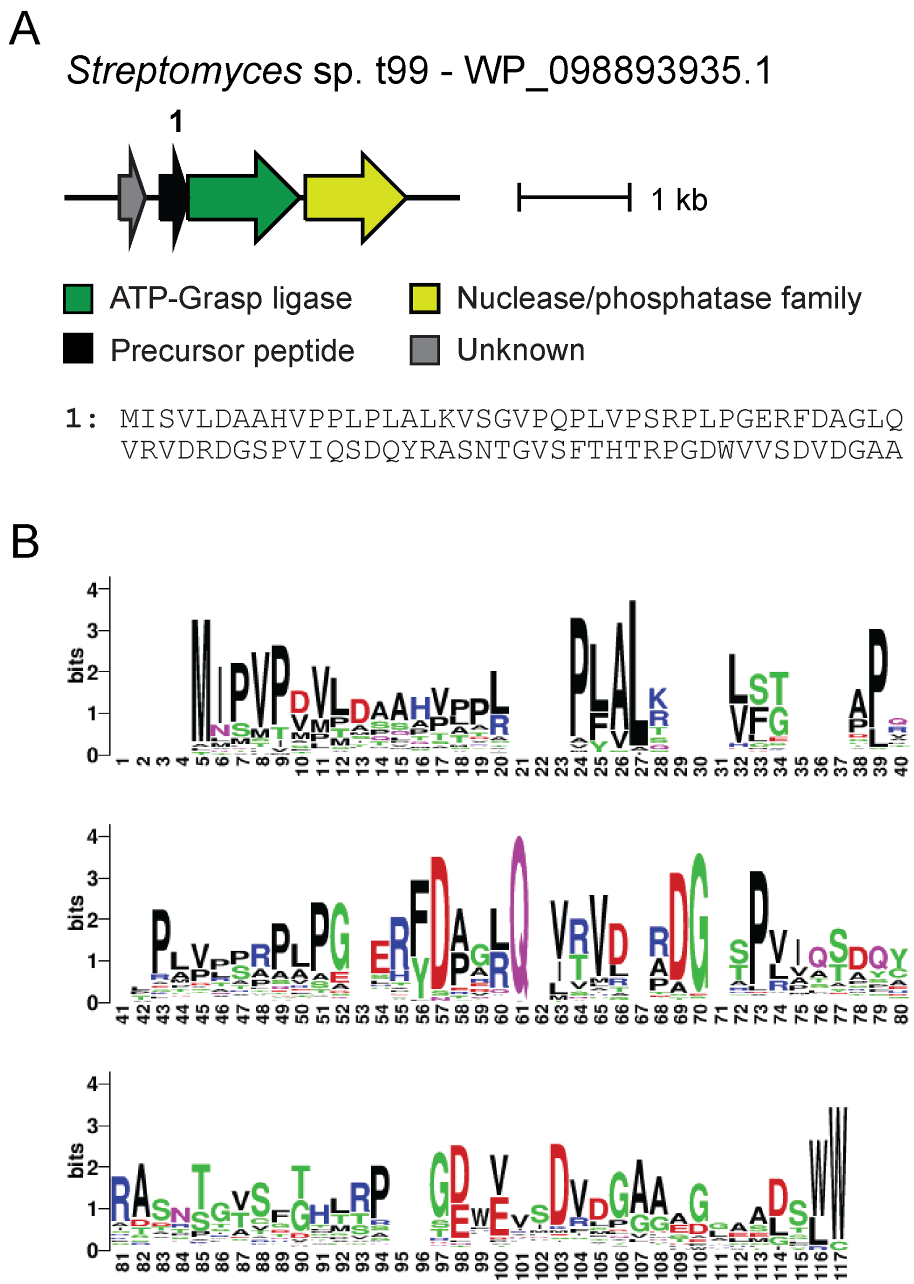

Fig. S6. Graspetide group 29 representative BGC and conservation logo. A) Representative BGC of graspetide group 29 from *Streptomyces* sp. t99 containing a predicted phosphatase family protein. B) Sequence logo (n = 86) for predicted precursor peptides for graspetide group 29.

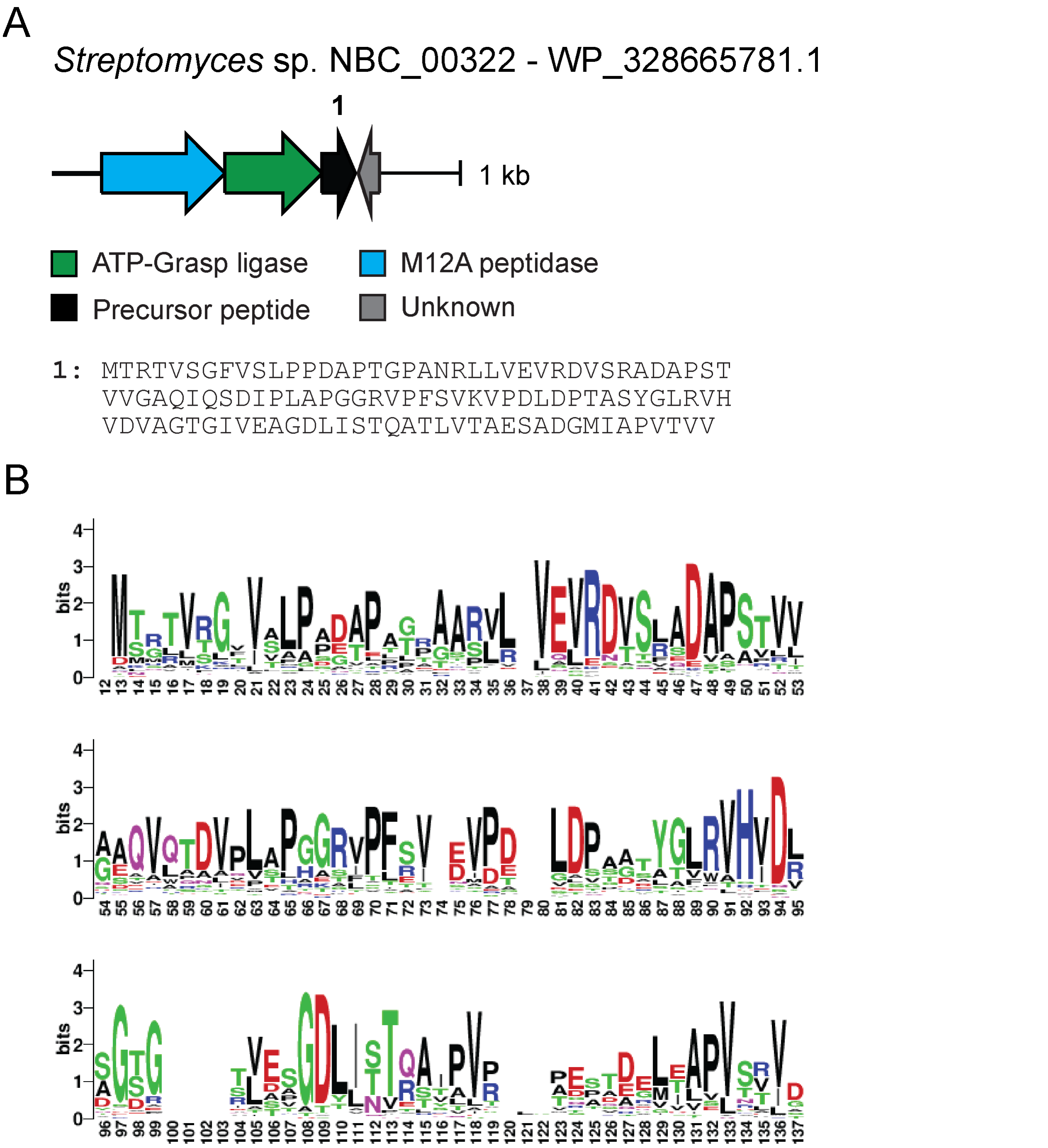

Fig. S7. Graspetide group 30 representative BGC and conservation logo. A) Representative BGC of graspetide group 30 from *Streptomyces* sp. NBC_00322 which contains an M12A peptidase. B) Sequence logo (n = 67) for predicted precursor peptides for graspetide group 30.

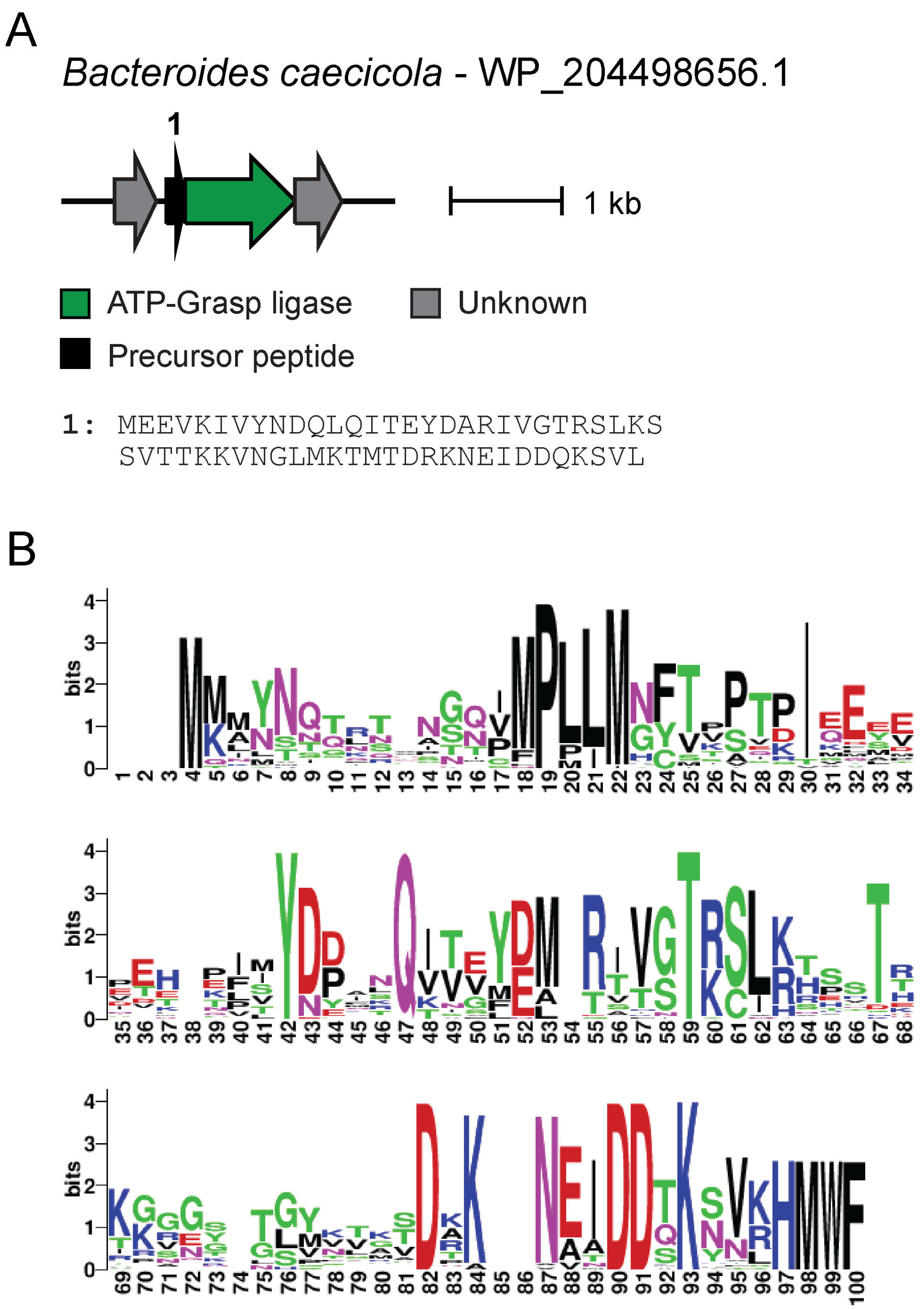

Fig. S8. Graspetide group 31 representative BGC and conservation logo. A) Representative BGC of graspetide group 31 from *Bacteroides caecicola* that contains several unknown proteins. B) Sequence logo (n = 43) for predicted precursor peptides for graspetide group 31.

A

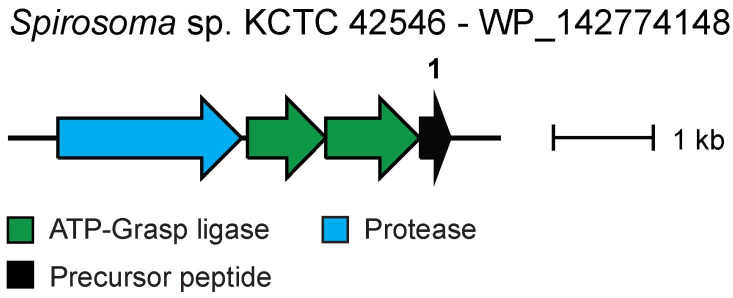


```
1: MQIIENWTRVRGIIKTFSAESDTDNFGEMALSI
   QQTSSLEGIADLISPKALTKARIYVPTADLADT
   TITPGQAVELILHITASGRLYAQPGSLHVLNE
```

B

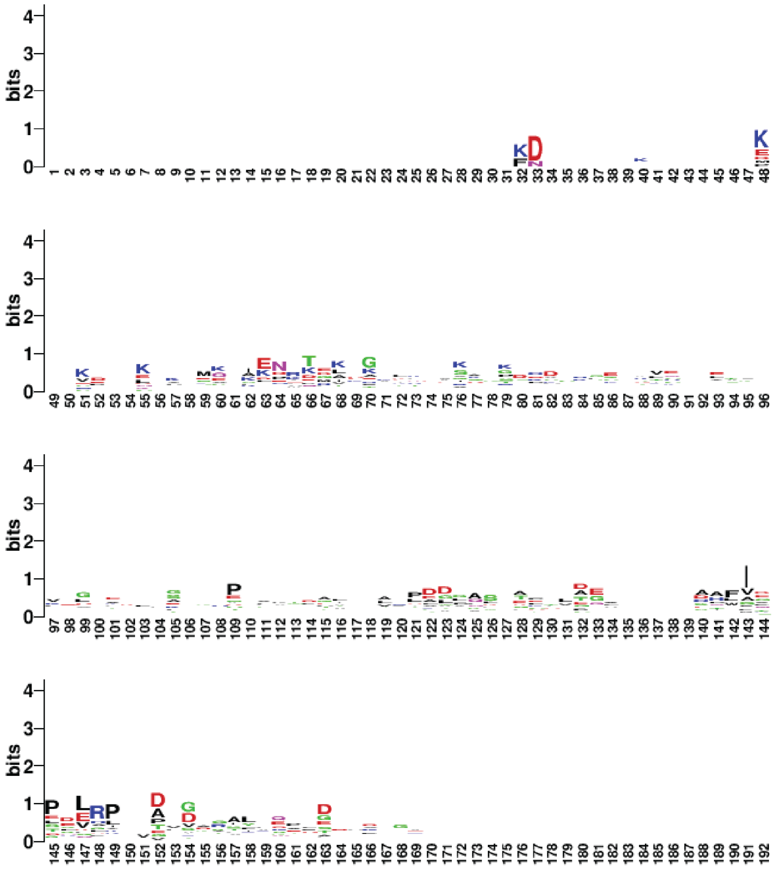

Fig. S9. Graspetide group 32 representative BGC and conservation logo. A) Representative BGC of graspetide group 32 from *Spirosoma* sp. KCTC 42546. Members of this group contain a diverse array of different proteases within each BGC. B) Sequence logo (n = 50) for predicted precursor peptides for graspetide group 32. The precursor peptides in this group are more divergent, with fewer conserved residues across the group.

A

Methanolobus zinderi - WP_176965570.1

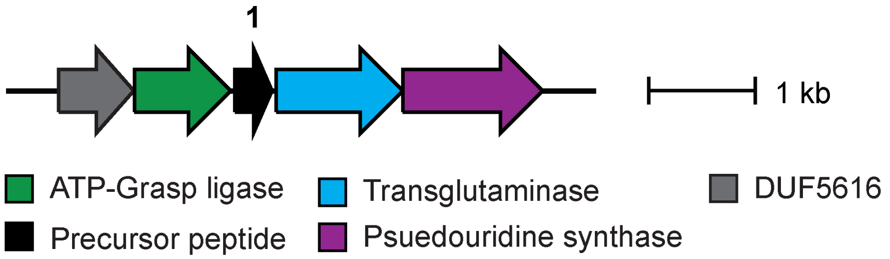


```
1: MSEYKQCIIVRDDLKLSKGKLAVQVAHAAVSASEWADRT
   TLEKWKEGGQKKVVLRVADTRGLFELKEIARKHNIATA
   LIQDAGLTEIKPGTVTVLGIGPAKEEELDRITGDLKLL
```

B

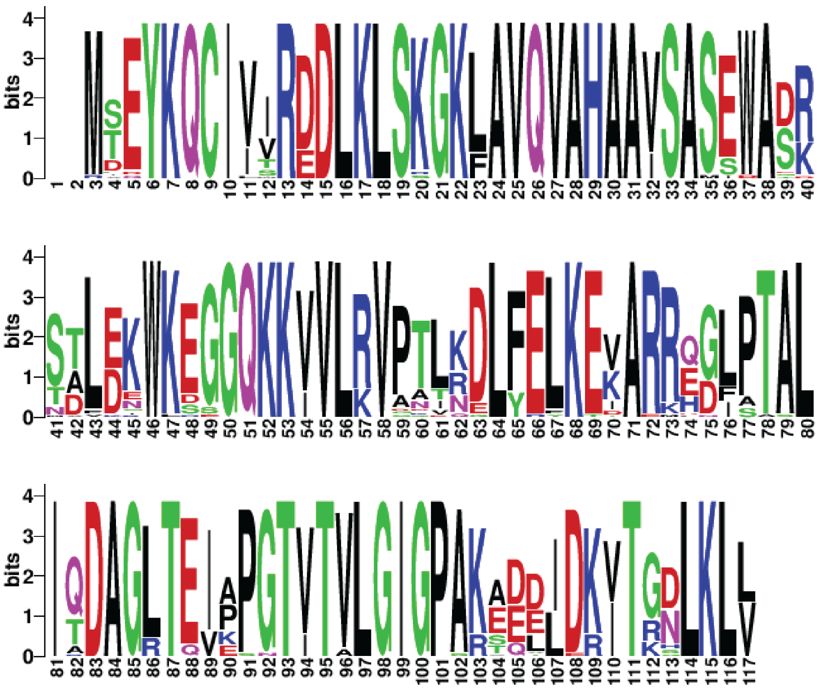

Fig. S10. Graspetide group 33 representative BGC and conservation logo. A) Representative BGC of graspetide group 33 from *Methanolobus zinderi* that contains a transglutaminase and psuedouridine synthase gene within the BGC. The precursor peptide also matches an HMM for a peptidyl-tRNA hydrolase. B) Sequence logo (*n* = 34) for predicted precursor peptides for graspetide group 33.

A

Streptomyces iakyrus - WP_033307070.1

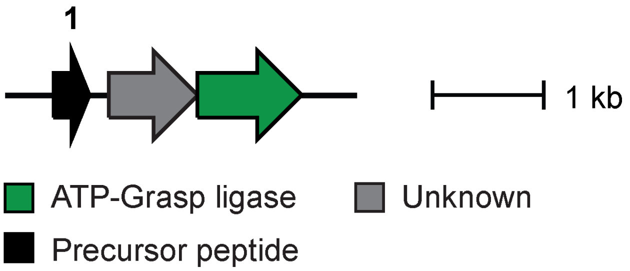


```
1: MTDKAFLSDFTSFPVVVQAAARRFATLQSGGAVGGV
   LPSLTRMKEGSEGSETSETSENSENSERSEFSEGSE
   FGERSTAGAIGRLGMSLSSAPVSEWGPVTSPVGRD
```

B

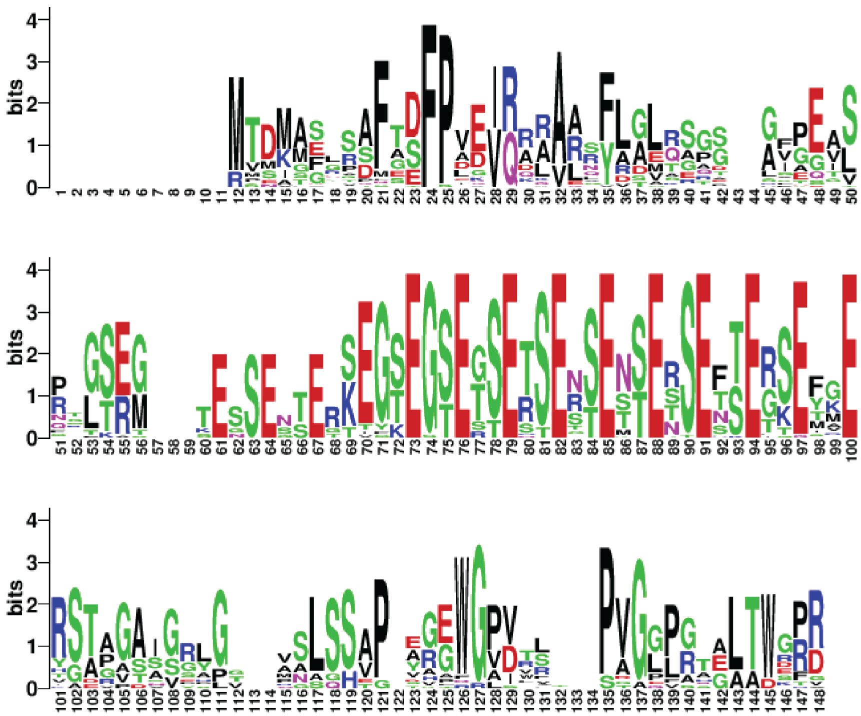

Fig. S11. Graspetide group 34 representative BGC and conservation logo. A) Representative BGC of graspetide group 34 from *Streptomyces iakyrus* which contains an unknown protein. B) Sequence logo (n = 37) for predicted precursor peptides for graspetide group 34.

A

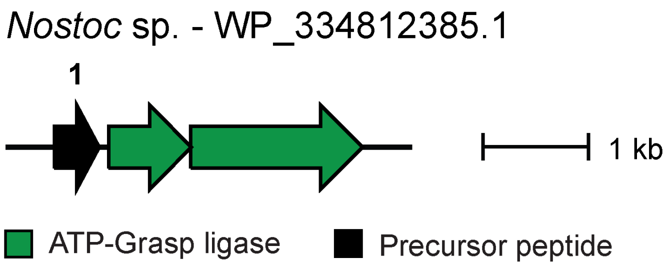


```
1: MKITSSTSTRLALVMGLAIASSIISTVAKAESIPQVNSHNSVSNKPF
   ISNAARRDTLYNLNQRPLNSVLETDGGKTKESSDKGKDSSDKGKDSK
   ETCESGNCGGGLIDRINPVILPADQLKIPVLRNARPQTLIKVNKPIF
```

B

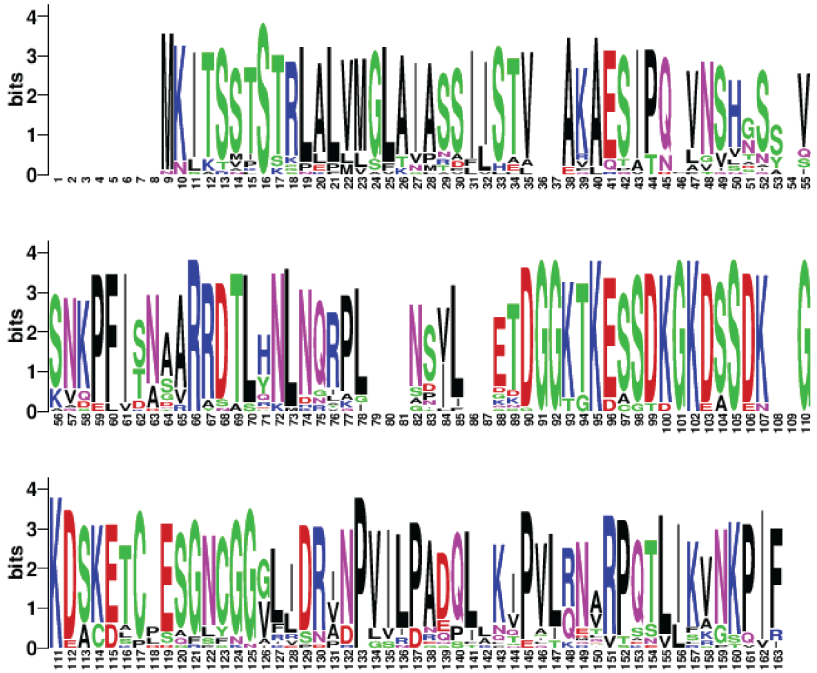

Fig. S12. Graspetide group 35 representative BGC and conservation logo. A) Representative BGC of graspetide group 35 from a *Nostoc* sp. that contains two graspetide synthetases and a large precursor peptide with repeating motifs with acceptor and donor residues. B) Sequence logo (n = 30) for predicted precursor peptides for graspetide group 35.

A

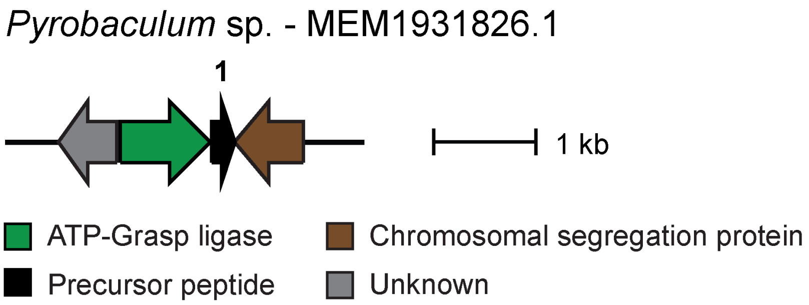

1: MTKWVLKCAACGEEKIFEAAFNLAPFGGRIYL
YCKKCRSNRDHVVLGCLDGEICQSAGADVID

B

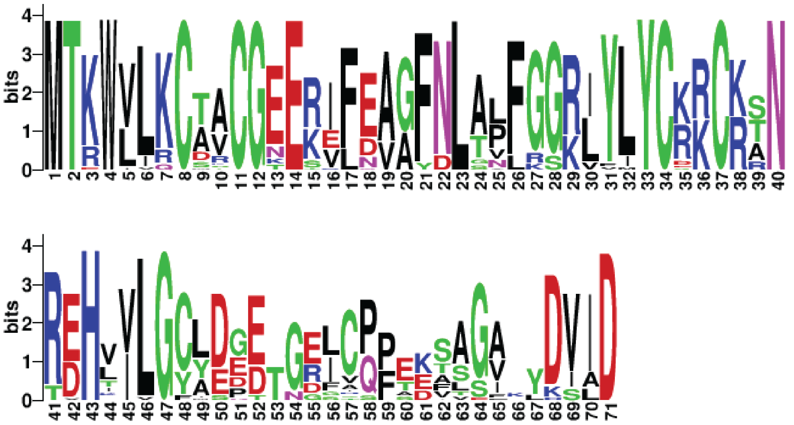

Fig. S13. Graspetide group 36 representative BGC and conservation logo. A) Representative BGC of graspetide group 36 from *Pyrobaculum* sp. that contains a predicted chromosomal segregation protein and an unknown protein. B) Sequence logo (n = 33) for predicted precursor peptides for graspetide group 36.

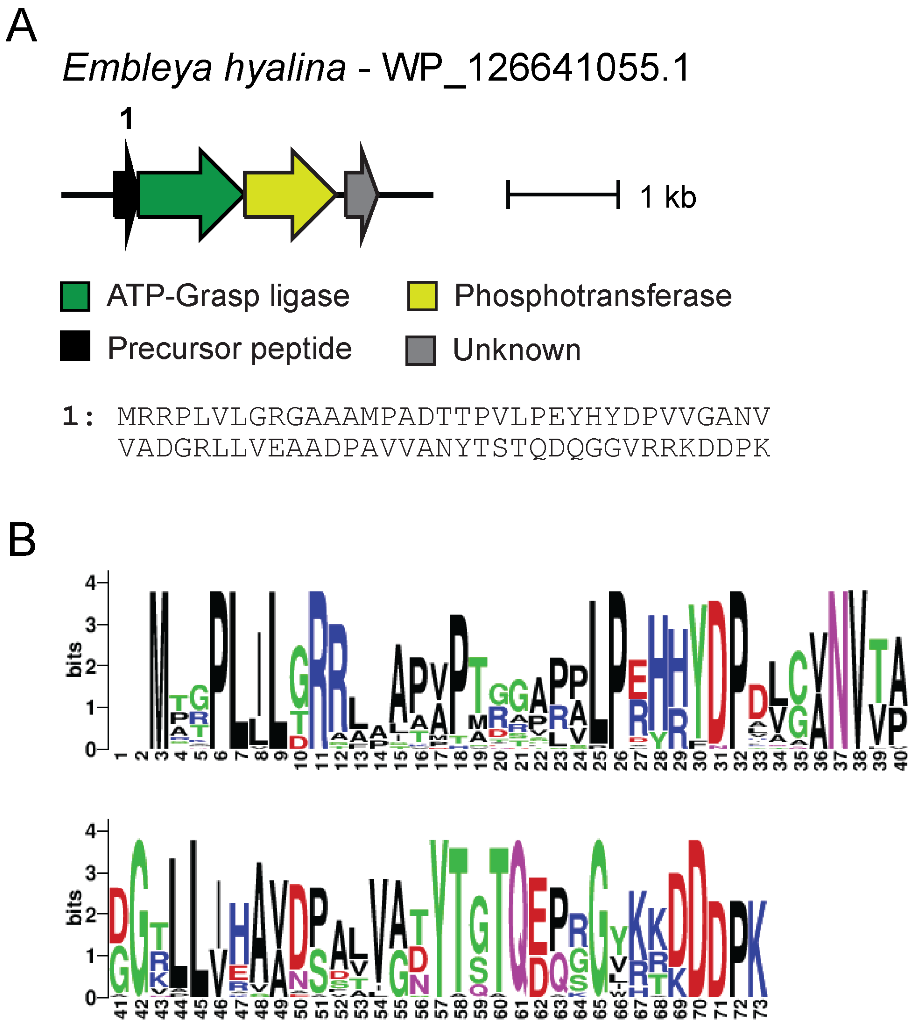

Fig. S14. Graspetide group 37 representative BGC and conservation logo. A) Representative BGC of graspetide group 37 from *Embleya hyaline* that contains a phosphotransferase protein. B) Sequence logo (n = 29) for predicted precursor peptides for graspetide group 37.

A

Micromonospora sp. NPDC005172 - WP_355618140.1

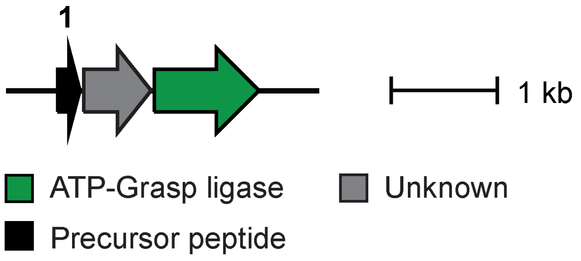

1: MNEQQLPPAQGDDGTAGGRTRRISVLE
ALADAARGVGPSRSNQSTVAAGDADDQ

B

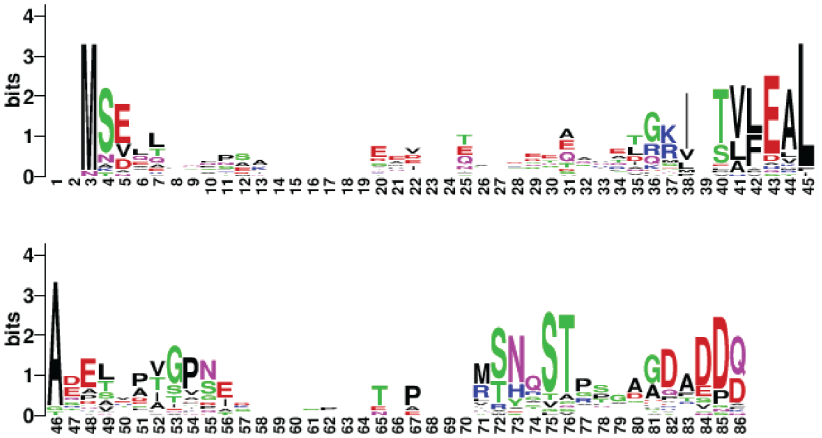

Fig. S15. Graspetide group 38 representative BGC and conservation logo. A) Representative BGC of graspetide group 38 from *Micromonospora* sp. NPDC005172 that contains an unknown protein. B) Sequence logo (n = 27) for predicted precursor peptides for graspetide group 38.

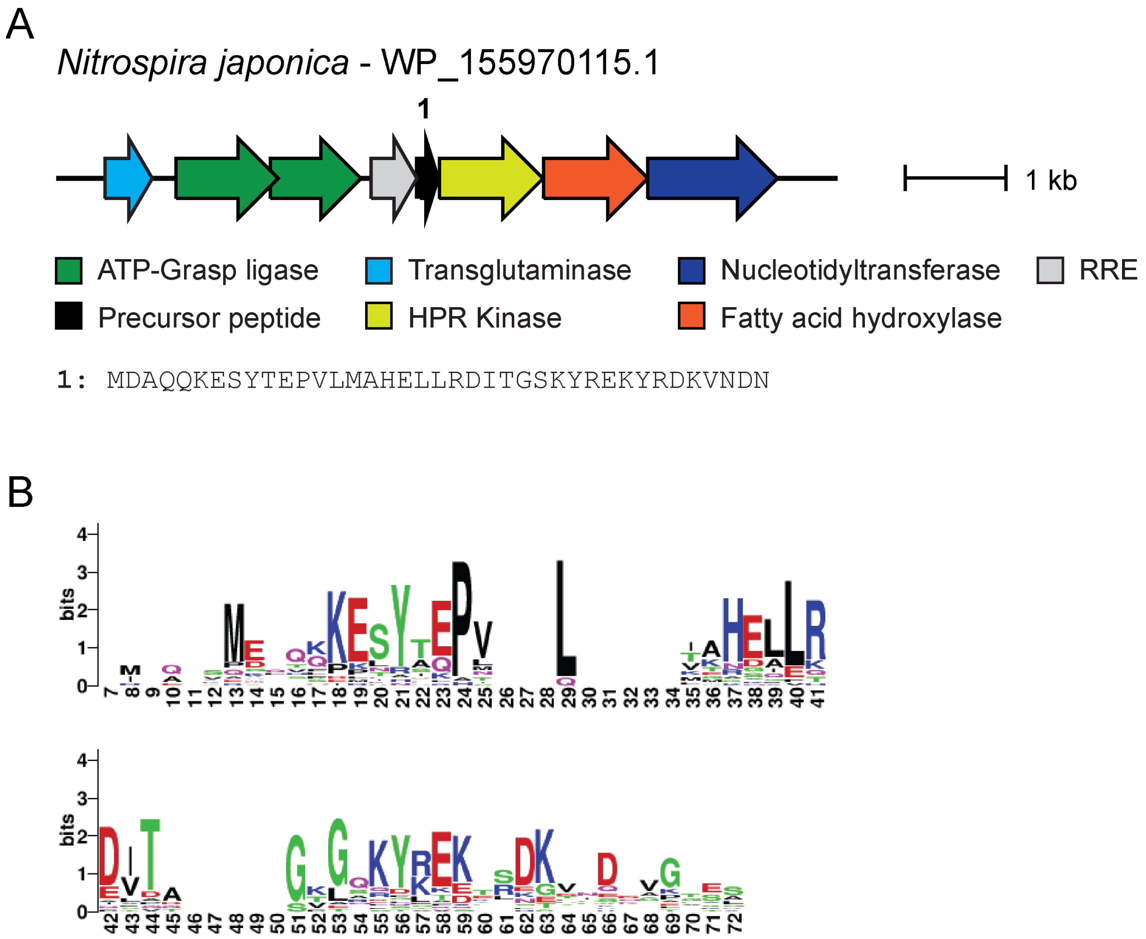

Fig. S16. Graspetide group 39 representative BGC and conservation logo. A) Representative BGC of graspetide group 39 from *Nitrospira japonica* that contains additional biosynthetic proteins associated with lasso peptides. B) Sequence logo ($n = 26$) for predicted precursor peptides for graspetide group 39.

A

Cystobacter ferrugineus - WP_071904411.1

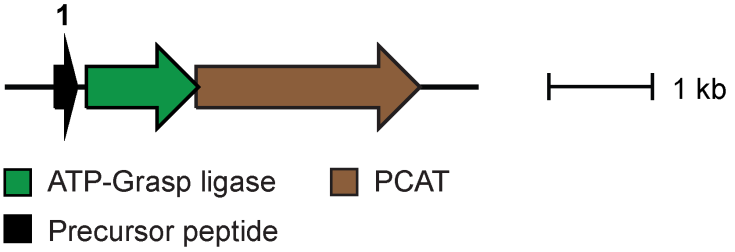

1: MLTETNNSQKSPIFGLQYIEDEEANLLDVVGCTG
LSTGSLEPTTTPTTPAVTIKLCGDGFDVSDMELSV

B

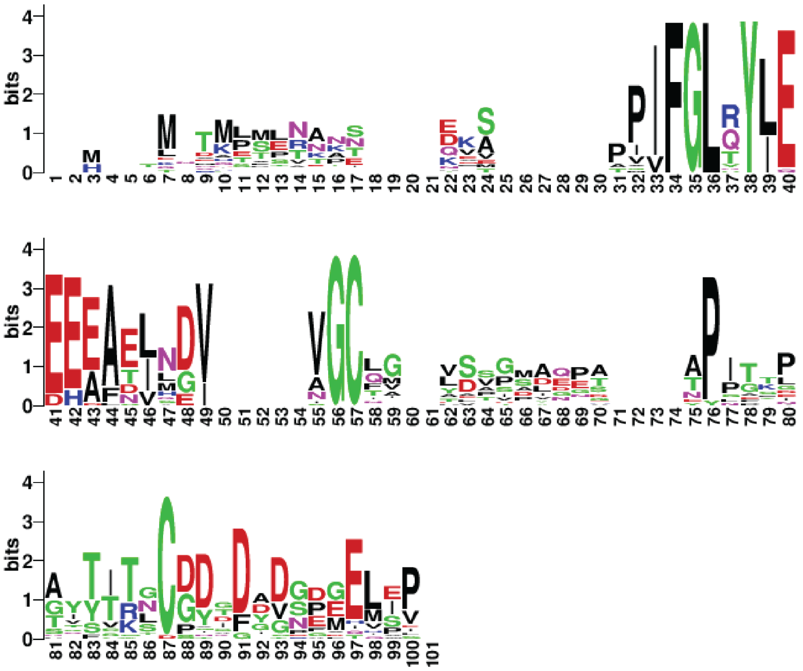

Fig. S17. Graspetide group 40 representative BGC and conservation logo. A) Representative BGC of graspetide group 40 from *Cystobacter ferrugineus* that contains a protease-containing ABC transporter (PCAT). B) Sequence logo ($n = 31$) for predicted precursor peptides for graspetide group 40.

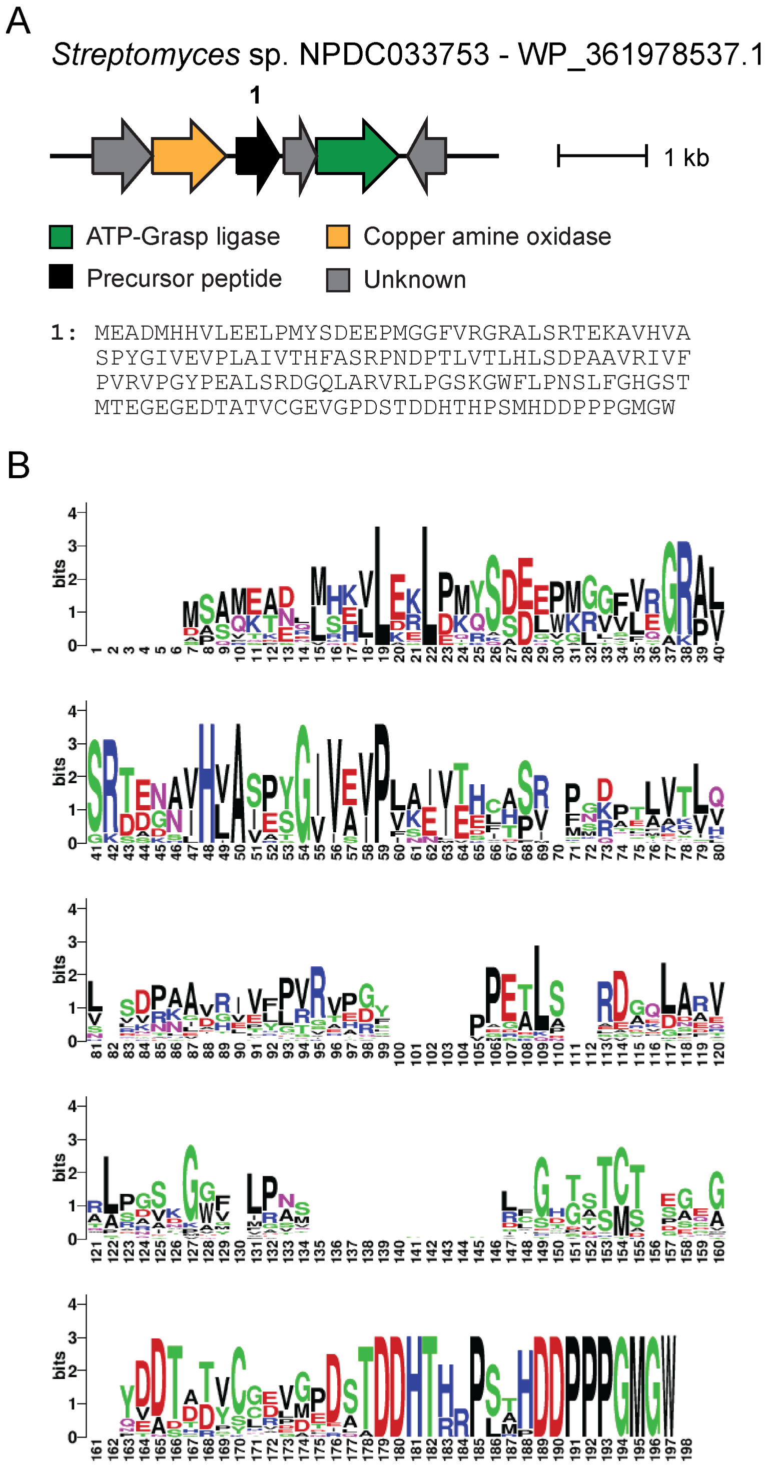

Fig. S18. Graspetide group 41 representative BGC and conservation logo. A) Representative BGC of graspetide group 41 from *Streptomyces* sp. NPDC033753 that contains several unknown proteins and a copper amine oxidase. B) Sequence logo (n = 33) for predicted precursor peptides for graspetide group 41.

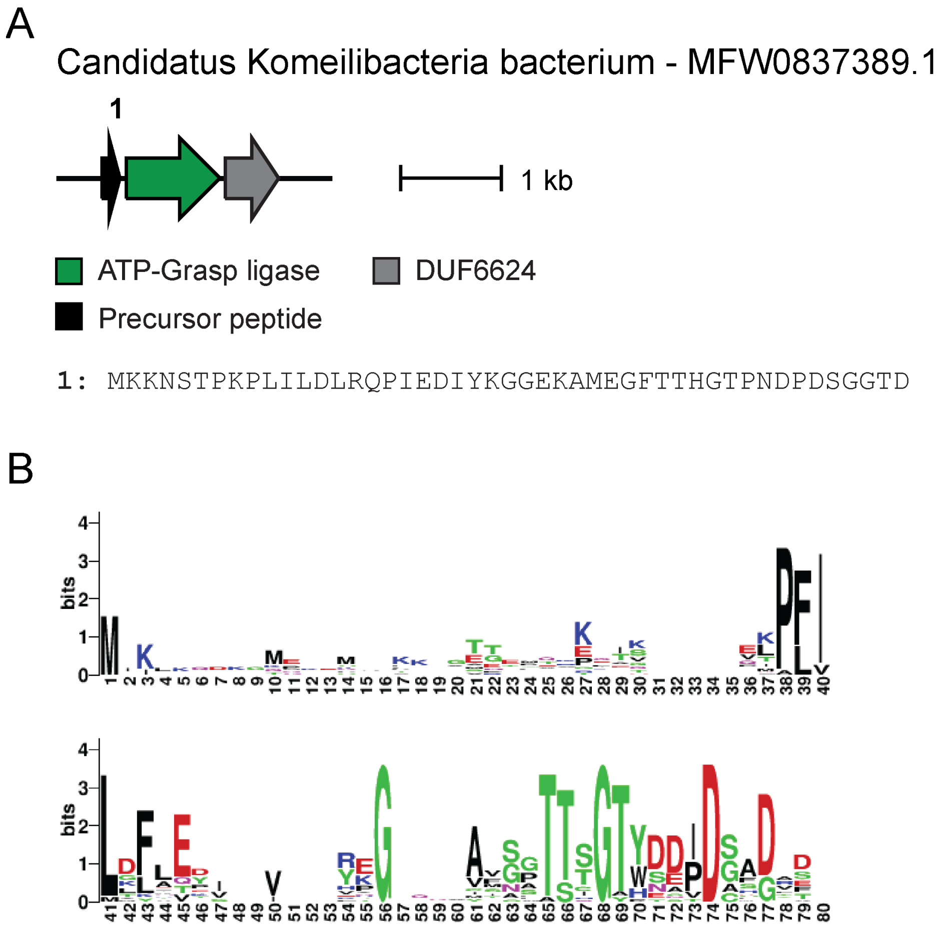

Fig. S19. Graspetide group 42 representative BGC and conservation logo. A) Representative BGC of graspetide group 42 from Candidatus *Komeilibacteria* bacterium that contains a DUF6624 protein. B) Sequence logo (n = 21) for predicted precursor peptides for graspetide group 42.

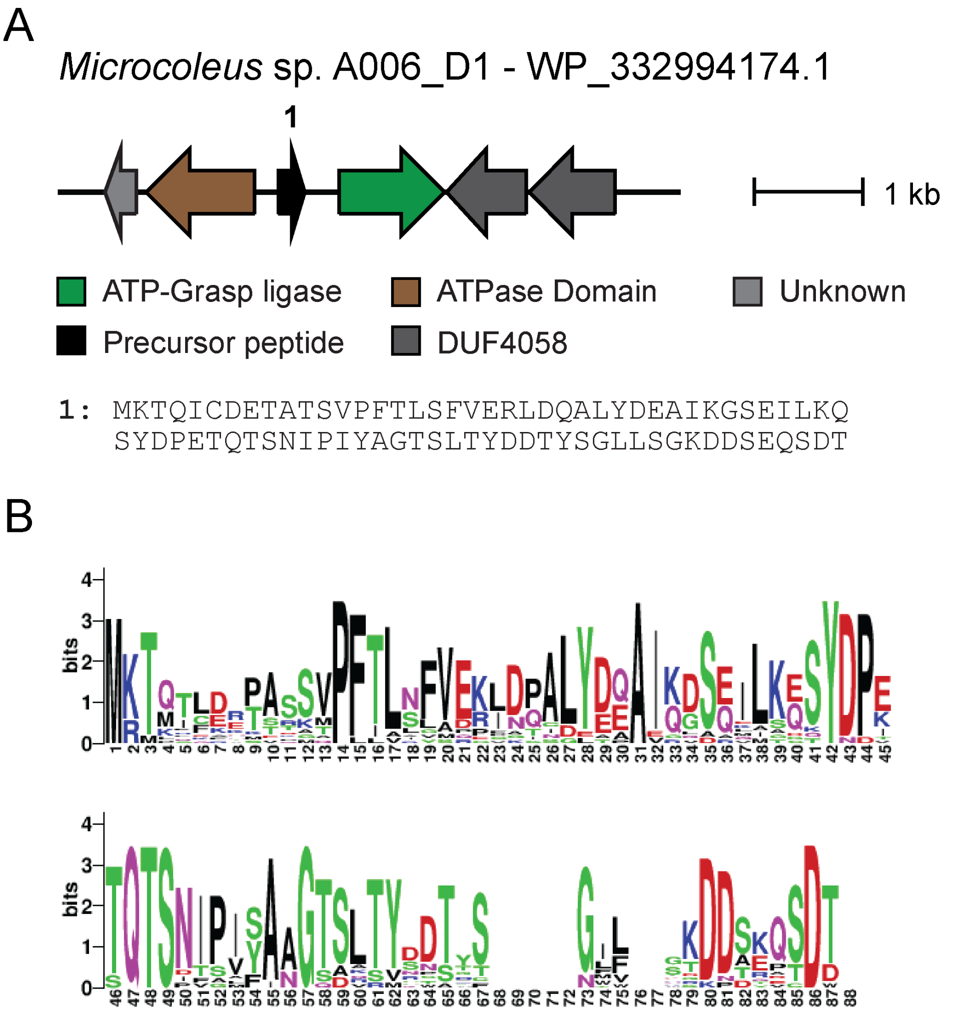

Fig. S20. Graspetide group 43 representative BGC and conservation logo. A) Representative BGC of graspetide group 43 from *Micocoleus* sp. A006_D1 that contains several DUF and unknown proteins. B) Sequence logo (*n* = 17) for predicted precursor peptides for graspetide group 43.

A

Candidatus *Angelobacter* sp. - HEV2964145.1

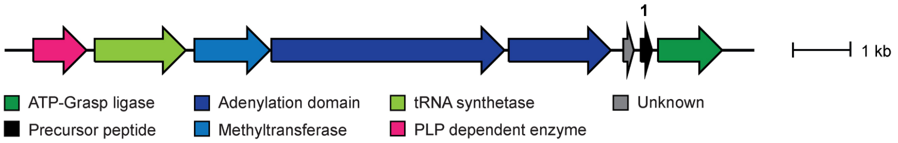

1: MSNRQDNRVLSRQLARDLTLQELETITGGFFTSPHRDVTTTFCTLASDGDDD

B

Fibrella aquatilis - WP_207337219.1

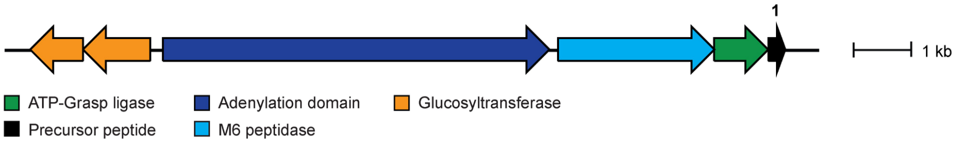

1: MQLIENRTQLTGLLQHIKPDSDIAGFGEVTLIVHHTAPLPGITDLLSAR
SPKTVCIYVPDAELTNPLLKLDETVTLVVRLASRDHFFAQPDTLRLAE

C

Embleya sp. NPDC127516 - WP_331771601.1

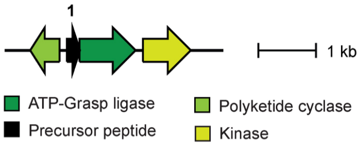

1: MPGPLILGRRAPVPTRGAPALPRHHYDPDLCVNVTPGGTLLIHAVDSGTYTGTQEPRGYKKDD

D

Armatimonadota bacterium - MBV9852473.1

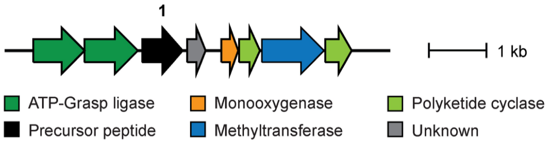

1: MANEPFIDPQSRPDLTVDLNSKVGDLSVRQLSDVLGKSTSANPPVKQVVEKQVVEKQIKDVPDKLPTKEHKEQKDAKD
TKDHKEPKDHKDTKDQKDHKEPKDTKDQKDQKEHKDQKDTPDHKQAKDHKDTKDTKDHKDSKDGIKDTIKDRIKEDIK
EFKEIEKQIKEEVKEVGKEAVKEDLEKLDDRPGDTNPGDQQSIDSLIQRVSGLEQQVAALQQGAGSAPAAASQAGGQG

E

Methanomethylovorans sp. - MBC7085990.1

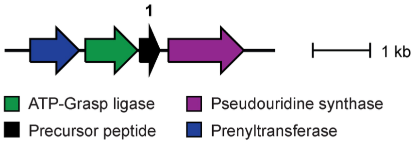

1: MDEYKQCIIVRDDLKLSKGKLAVQVAHAAVSASSWASKSVLENWKESGQKKVVLRVPS
LKDLFELKEKARRDGLPTALIADAGLTEVEPGTITALGIGPGKAQDIDKITKDLKLL

F

Streptomyces sp. NPDC018045 - WP_398352607.1

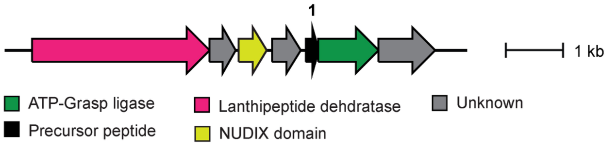

1: MAFQQCTSSRPFALRLFTAPVTSQCRSLPGTYDPQLQMTVTADGTPRIATSYAARSQTCGGLTTQTEIEIMDVC

G

Streptomyces rimosus - WP_003979389.1

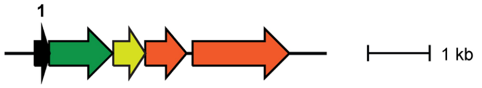

ATP-Grasp ligase; PhyH dioxygenase; Precursor peptide; NUDIX domain

1: MPTALPAPAARPYVLSHLTPLLPGTGGATAPQHRYDPARQ
LVVDDTGAPVVSSPVSTIMATGHLGPHEKADRPDGMDSWW

H

Frankia nepalensis - WP_203004092.1

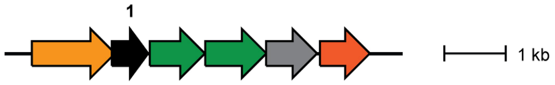

ATP-Grasp ligase; PhyH dioxygenase; Unknown; Precursor peptide; Monooxygenase

1: MSWTIDETRVRPFLSAADRRDLLTLLRRPGGTGQEPLGDAVATPAPGRAGLAGTGLATPRPATADP
VAQAPKWKDLKDGKDFKDHKDNKDNKDWKDLKDWKDRKDSKDFKDTKDSKDFKDFKDGIDGAHPG
AAAGRAPDDRPAAGLAPAGPGDAATAGMVTGDEPTRPGVIDEDDIGAVLRASRVNRYLRAAGVVI

I

Streptomyces cinerochromogenes - WP_392819198.1

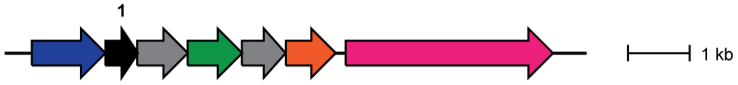

ATP-Grasp ligase; PhyH dioxygenase; Pyruvate decarboxylase; Precursor peptide; F420 Oxidoreductase; Unknown

1: MTWTIDEARVMPFLKAAHRRDLLAALRRPAPAPAPAPAADEGTPGPHAKSFKDFKDGKE
FKDYKDRKDFKDHKDLKDLKDRKDGKDRKDTKDGKDFKDSKDGTDGAWTPTGPAAPLTA
RPAERTTAMPAGTDDGTGIGTDTDDGGDARTDAGSDDVLSVLRASRASRFLRERGLVI

J

Phycicoccus elongatus - WP_395653719.1

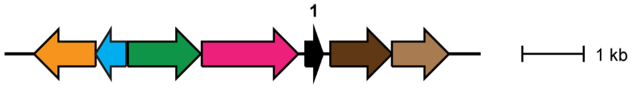

ATP-Grasp ligase; Aspartoacylase; Demethylase/hydroxylase; Ferrodoxin-like; Precursor peptide; Oxidoreductase; ATP-dependent Zn protease

1: MTTTPDAPLEDDDLTTSPSAGAGAGATGADADGLDADGTDGGDSDGT
DGGDADGTDGTDGDGTDGTDGDGTDGDGTDGGDVDGTDGTDGDGTDA

K

Tabrizicola caldifontis - WP_135450868.1

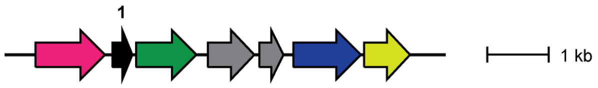

ATP-Grasp ligase; Alcohol dehydrogenase; Nuclease; Precursor peptide; Acetyltransferase; Unknown

1: MSDMSRGQVSALLPAVLVASGLSLAAAGPALAVGSAVVPATGELPAVMQALPQG
FAIPDRLAAYEREQVEETQVAMRFLDWLFGDEPVNRCGTTSGCRSGWDDSDCF

L

Streptomyces sp. DSM 110735 - WP_199582090.1

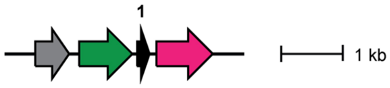

ATP-Grasp ligase; Dehydogenase; Precursor peptide; Unknown

1: MDEQQQPETQKISLFEALADELVPTGPNMSNQSTPSAGDADDQ

Fig. S21 (previous two pages). Graspetide BGCs with additional tailoring enzymes. A-F) Graspetide BGCs in new graspetide groups with additional modifying enzymes found in other natural product groups. G-L) Graspetide BGCs in new graspetide groups with additional oxidative enzymes.

M+H: 2379.07 Da NGAGSSKTIDDIPGGGRPDTDSDF

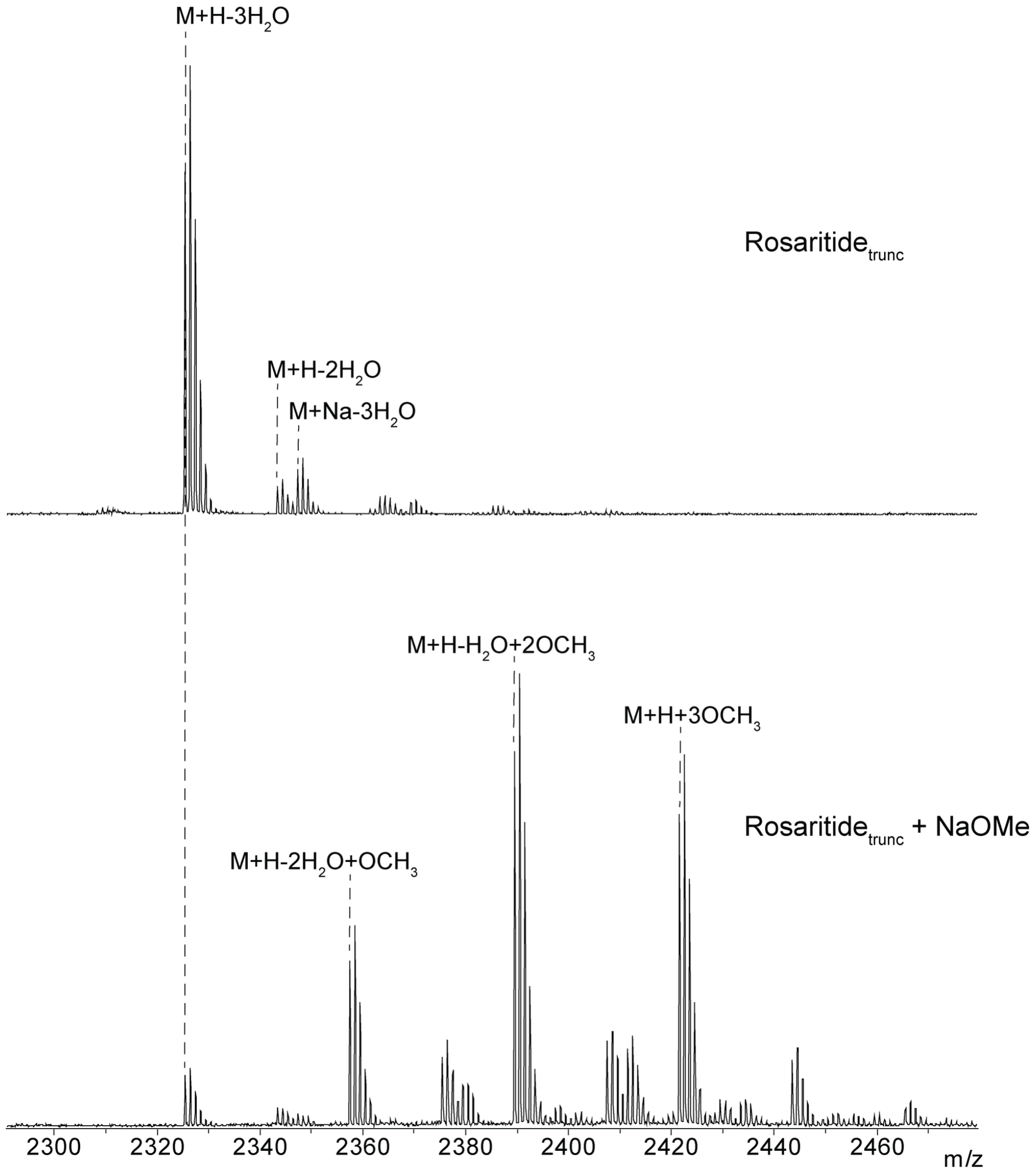

Fig. S22. Alkaline methanolysis of rosaritide$_{\text{trunc}}$. MALDI-TOF mass spectra of a tryptic fragment of rosaritide$_{\text{trunc}}$ containing up to three graspetide linkages (*top*) subjected to alkaline methanolysis (*bottom*).

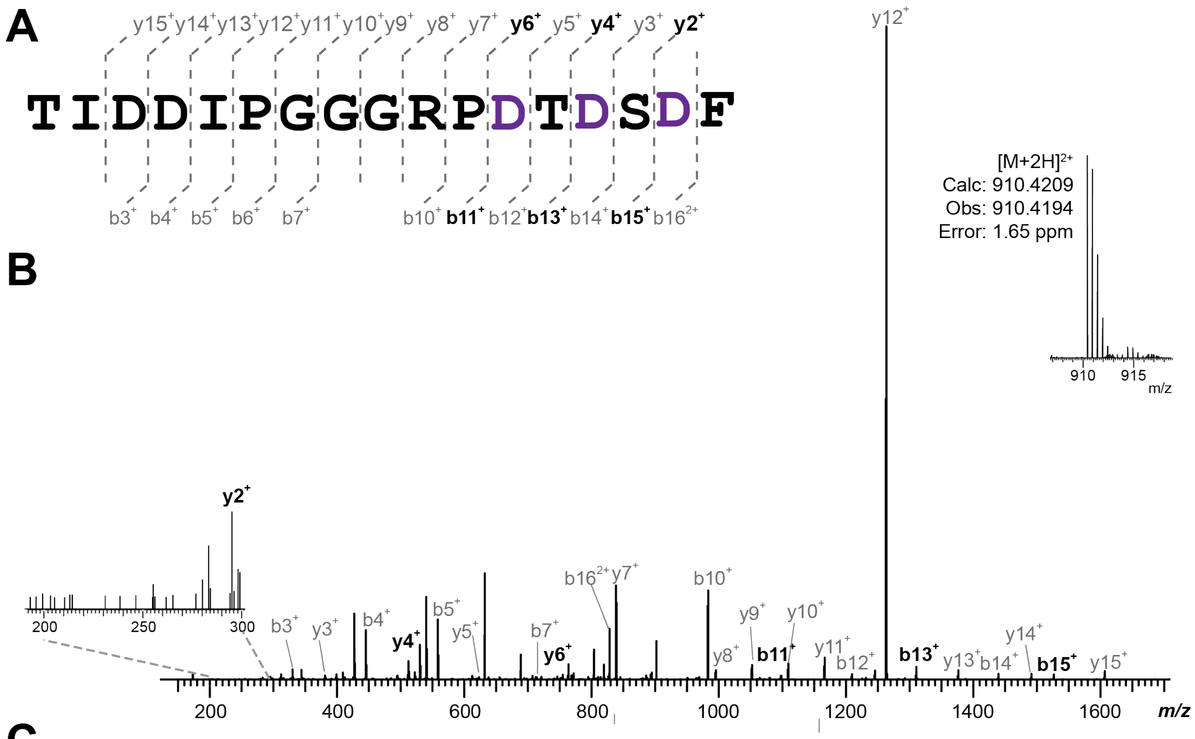

C

Ion	Calc.	Obs.	Error in ppm	Ion	Calc.	Obs.	Error in ppm
$[M+2H]^{2+}$	910.4209	910.4194	1.65	$y15^+$	1605.7028	1605.7003	1.52
				$y14^+$	1490.6758	1490.6736	1.50
$b3^+$	330.1660	330.1655	1.45	$y13^+$	1375.6489	1375.6467	1.56
$b4^+$	445.1930	445.1921	1.95	$y12^+$	1262.5648	1262.5625	1.83
$b5^+$	558.2770	558.2760	1.81	$y11^+$	1165.5121	1165.5098	1.96
$b6^+$	655.3298	655.3284	2.06	$y10^+$	1108.4906	1108.4885	1.86
$b7^+$	712.3512	712.3497	2.12	$y9^+$	1051.4691	1051.4673	1.74
$b10^+$	982.4953	982.4934	1.90	$y8^+$	994.4477	994.4459	1.75
$b11^+$	1079.5480	1079.5441	3.68	$y7^+$	838.3466	838.3450	1.81
$b12^+$	1208.5906	1208.5884	1.86	$y6^+$	741.2938	741.2924	1.92
$b13^+$	1309.6383	1309.6365	1.40	$y5^+$	612.2512	612.2501	1.75
$b14^+$	1438.6809	1438.6790	1.35	$y4^+$	511.2035	511.2026	1.88
$b15^+$	1525.7129	1525.7106	1.55	$y3^+$	382.1609	382.1603	1.75
$b16^{2+}$	827.8814	827.8803	1.34	$y2^+$	295.1289	295.1284	1.80

Fig. S23. HRMS/MS of fully methanolized rosaritide$_{trunc}$. A) Fragmentation of fully methanolized rosaritide$_{trunc}$. Observed b- and y-ions are annotated, with fragments bolded that are crucial to identify each Asp methyl ester; Asp methyl esters are also indicated in purple. B) Annotated CID spectrum of fully methanolized rosaritide$_{trunc}$. C) Table of daughter ion assignments.

M+H: 5994.97 Da

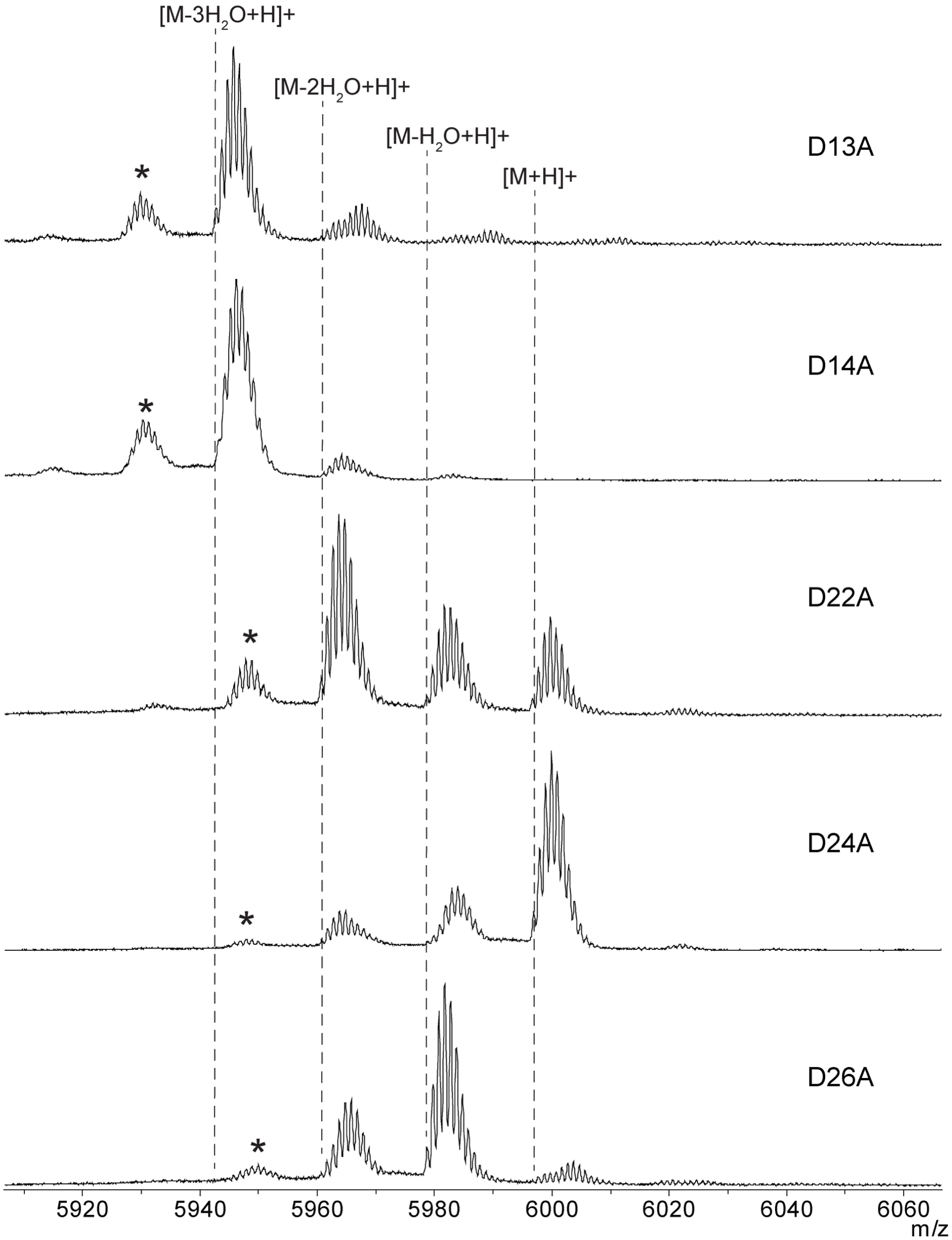

Fig. S24. MALDI-TOF mass spectra of putative acceptor-to-Ala MirA variants. MALDI-TOF mass spectra of Ala-substituted variants of putative macrolactone acceptor residues in MirA following co-expression with MirBC and MBP tag removal.

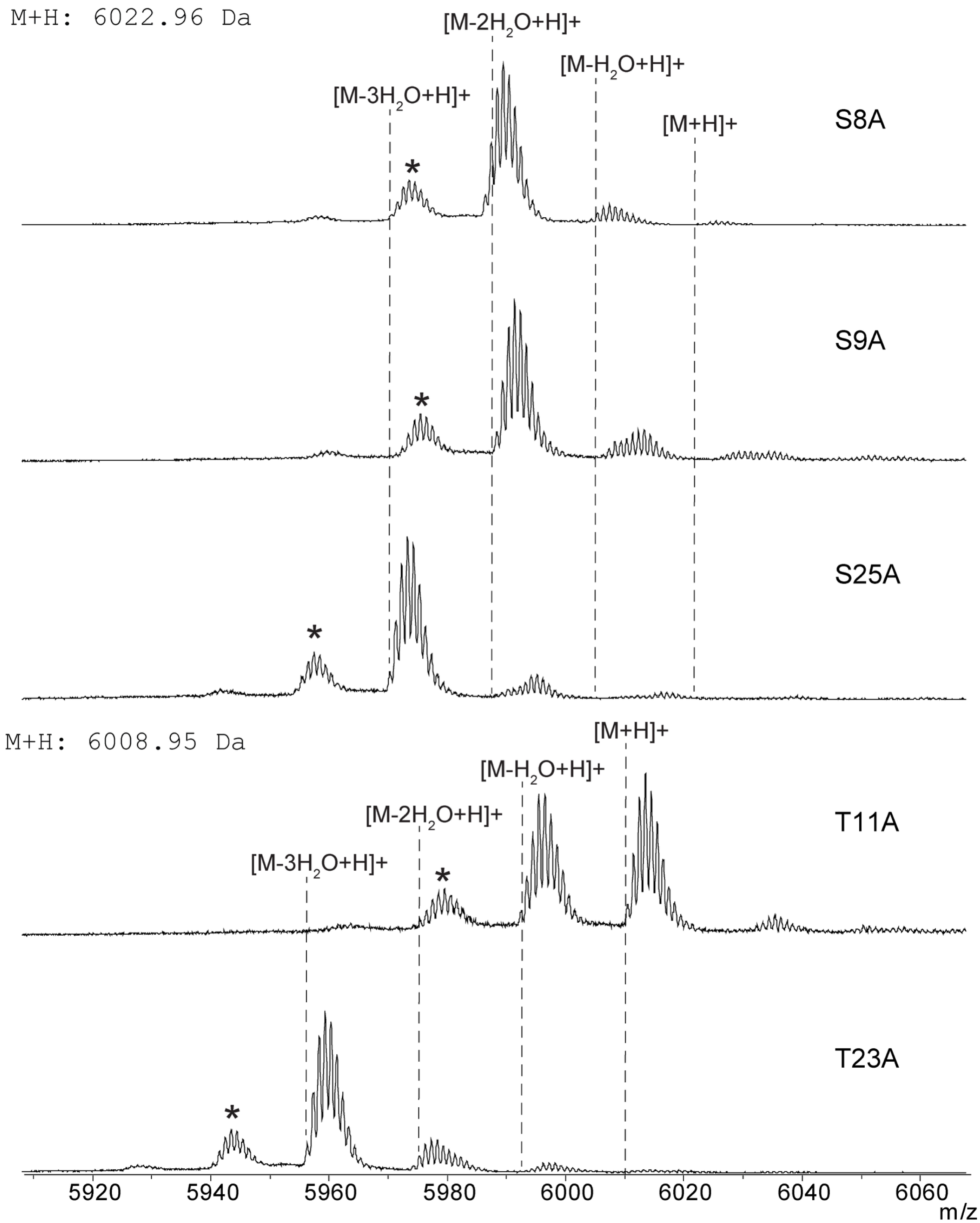

Fig. S25. MALDI-TOF mass spectra of putative donor-to-Ala MirA variants. Ala-substituted variants of putative macrolactone donor residues in MirA following co-expression with MirBC and MBP tag removal.

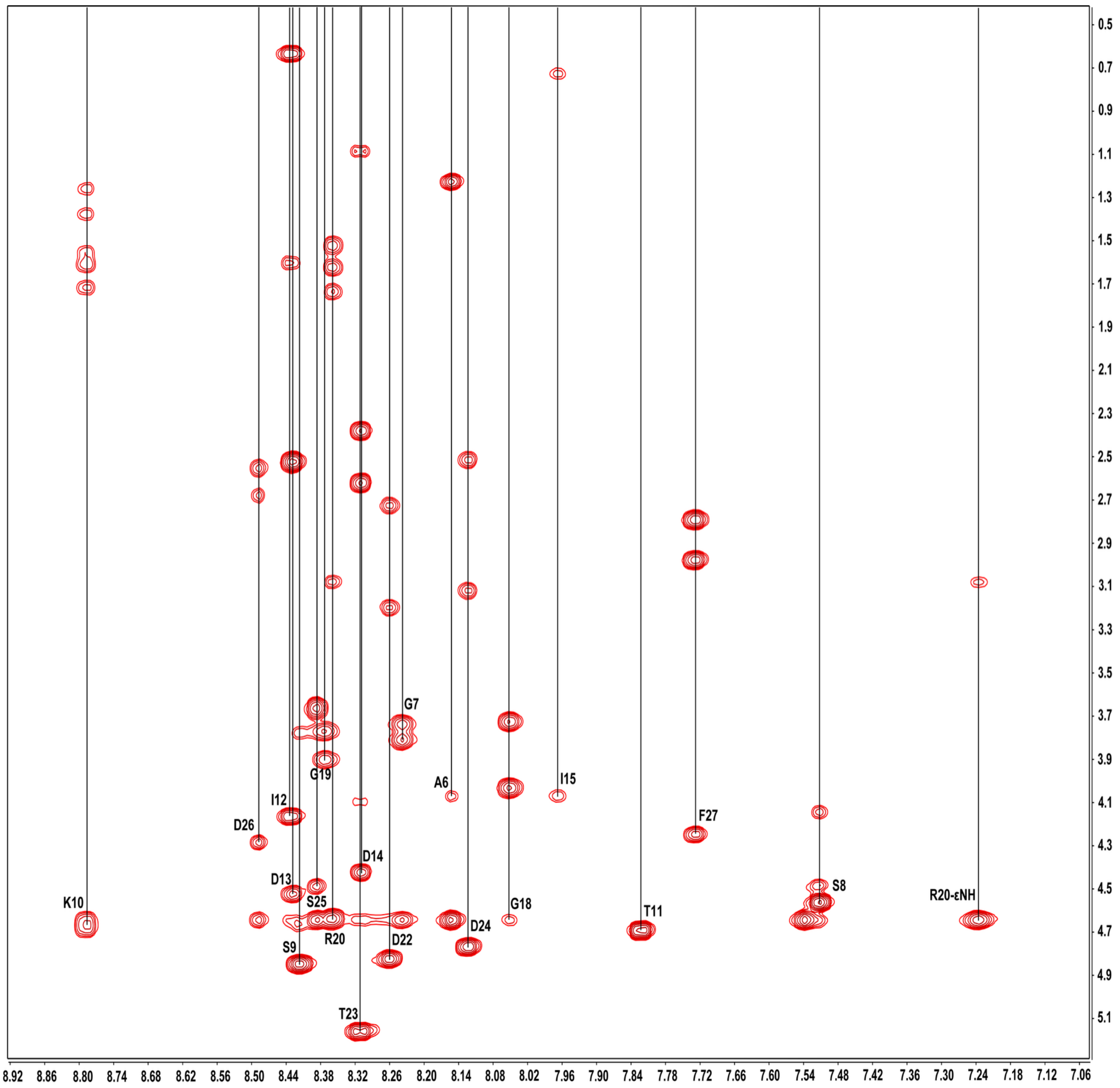

Fig. S26. [1]H-^{1}H TOCSY correlations of rosaritide$_{trunc}$. TOCSY correlations are labeled by each amino acid spin system.

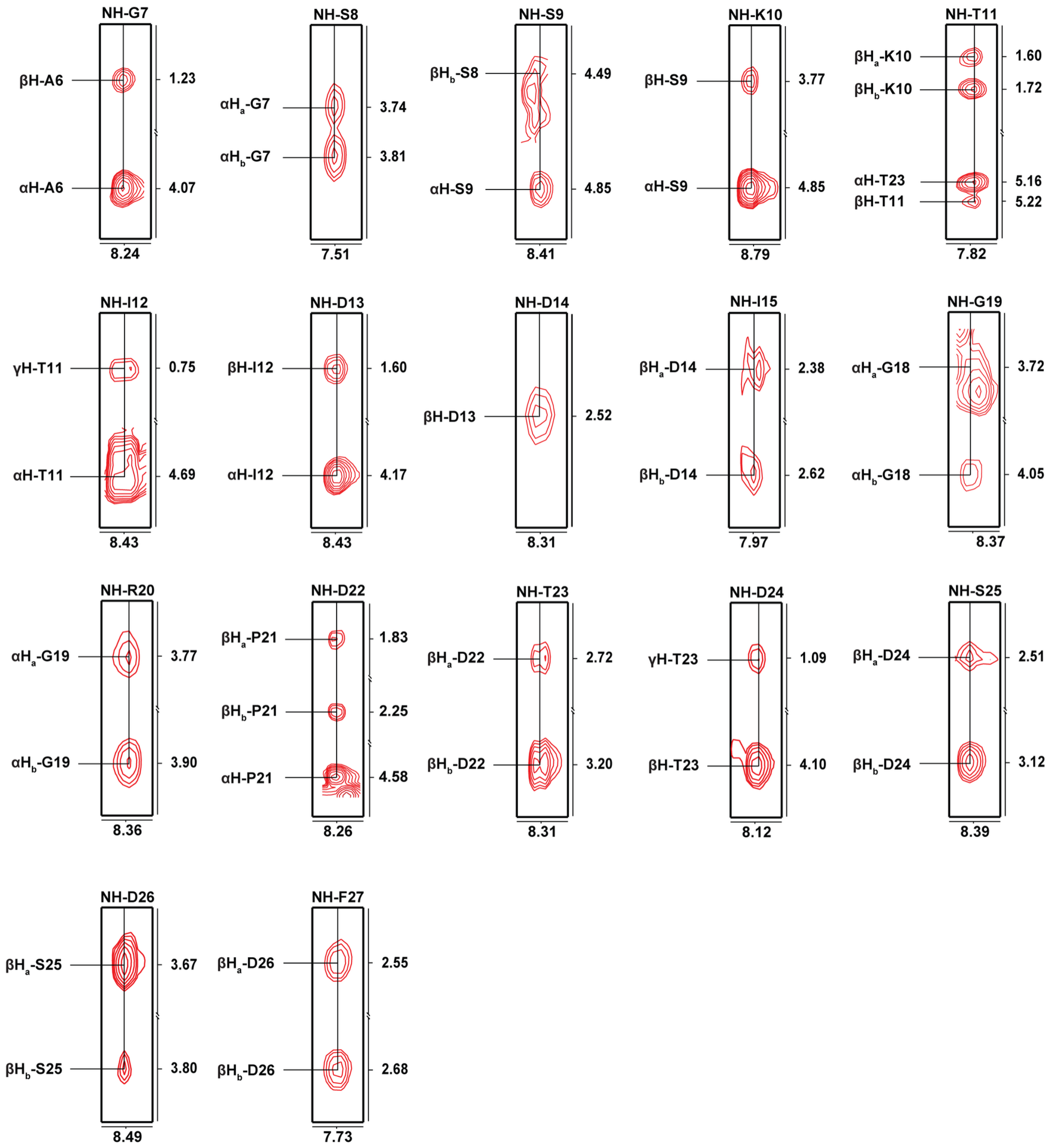

Fig. S27. ^1H-^1H NOESY correlations of rosaritide$_{trunc}$. NOESY correlations used to assign the linear sequence of amino acids in rosaritide$_{trunc}$.

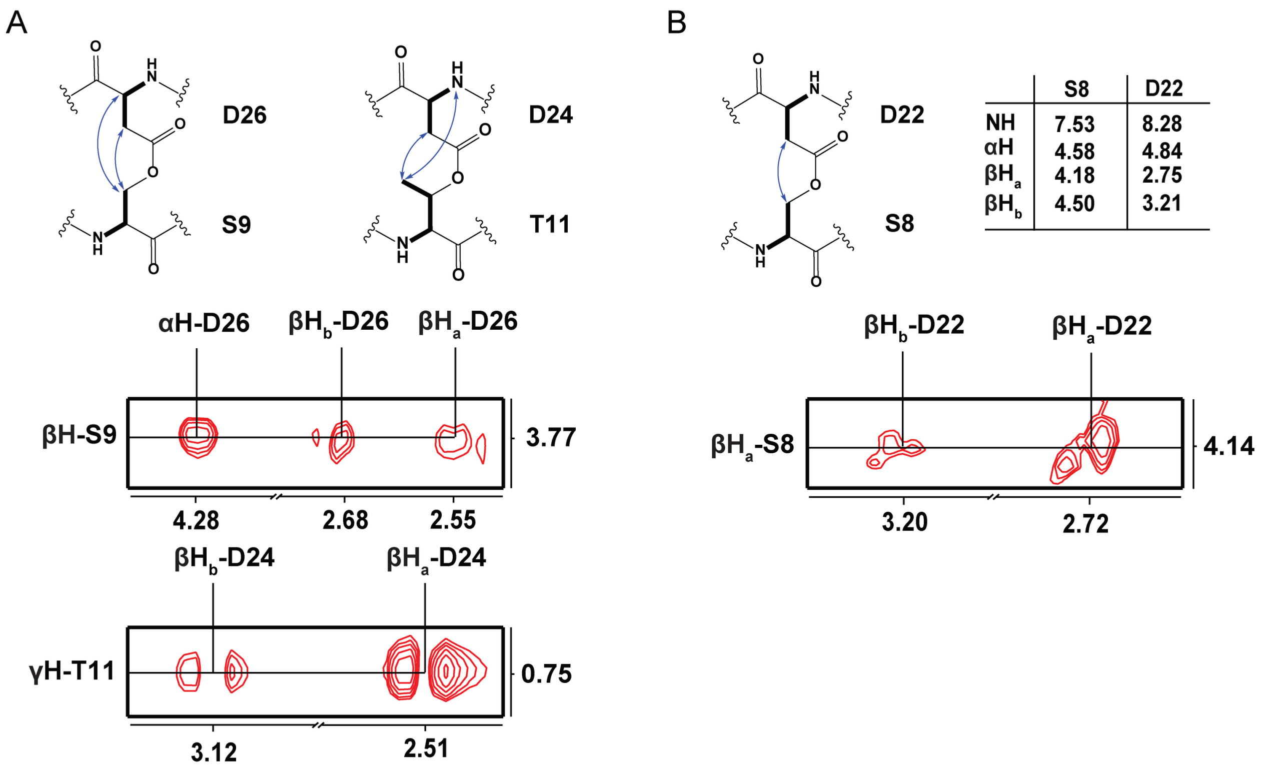

Fig. S28. NMR correlations of rosaritide$_{trunc}$ macrolactone linkages. NOESY correlations used to establish the location of the macrolactone linkages in rosaritide$_{trunc}$. A) NOESY correlations collected using 300 ms mixing time and B) with 500 ms mixing time.

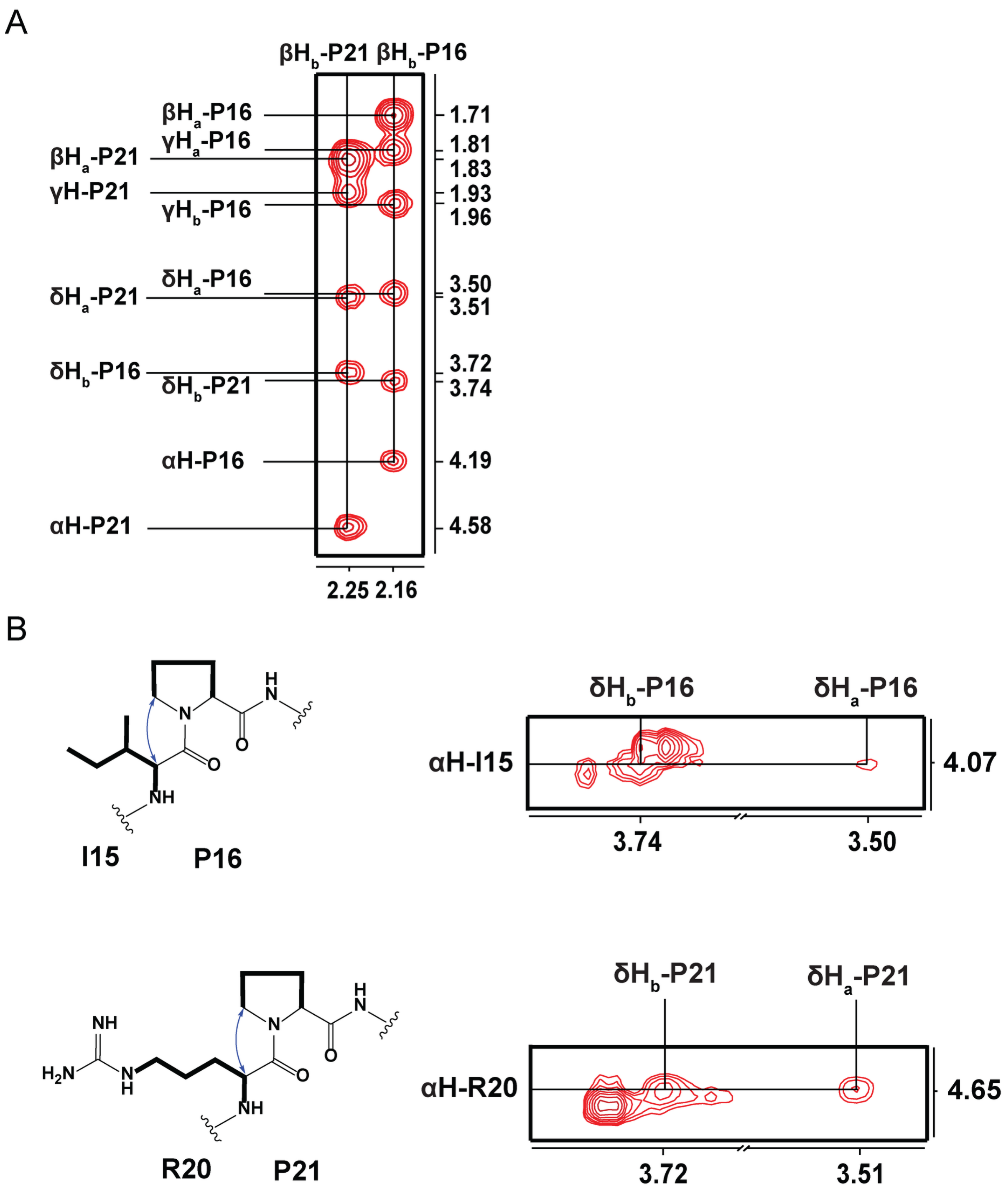

Fig. S29. ^{1}H-^{1}H correlations of Pro residues in rosaritide$_{trunc}$. A) TOCSY correlations of the Pro residues in rosaritide$_{trunc}$. B) NOESY correlations used in assigning each Pro residue to the trans configuration.

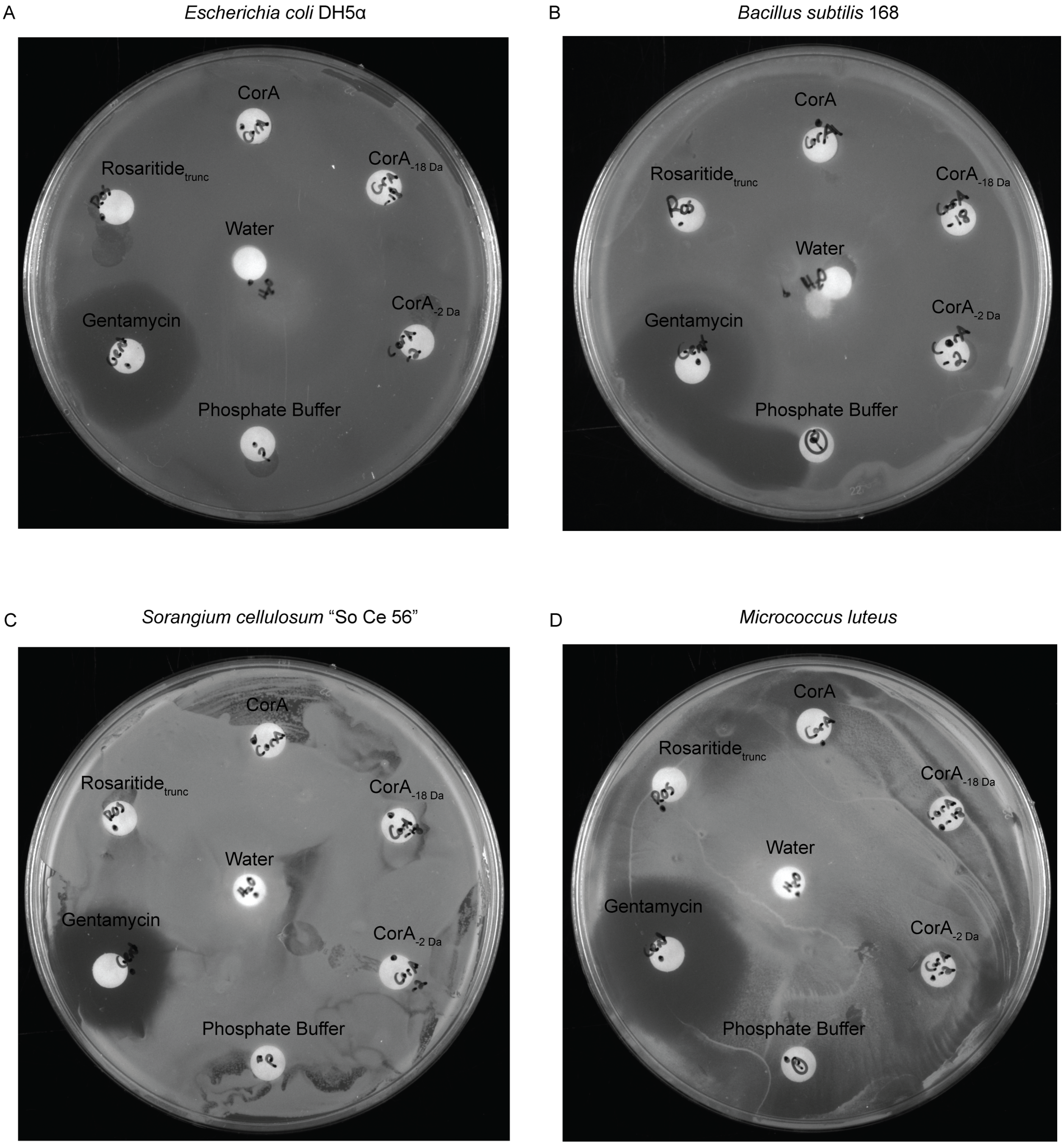

Fig. S30. Rosaritide$_{trunc}$ & corallotide bioactivity assay. An agar disk diffusion assay was performed on Hinton Mueller agar using 100 µg of each of the following rosaritide$_{trunc}$ (Ros), corallotide (CorA -2), unmodified CorA (CorA), and dehydrated CorA (CorA -18). The compounds were tested for bioactivity against A*) E. coli* DH5α, B) *Bacillus subtilis* 168, C) *Sorangium cellulosum* "So Ce 56", and D) *Micrococcus luteus*. Sterile water (H_2O) and 20 mM potassium phosphate pH 7.5 (P) were used as negative controls, and 30 µg of gentamycin (Gent) was used as a positive control. A faint ring of growth inhibition was observed for *M. luteus* with CorA derivatives, but non-uniform growth in the plate complicate interpretation of results.

```
Group-1  M-----TVLIFTFSNDNESIPLVVKAIEAKGEKAFRFDTDRFPT--EVKLDIYSGDFEGG
Group-2  MTNLDTSIVVVGSPDD-LHVQSVTEGLRARGHEPYVFDTQRFPE--EMTVSLGEQGASIF
Group-3  M----KTILIITNTLD-LTVDYIINRYNHTA-KFFRLNTDRFF---DYDINITNSGTSIR
Group-4  MM---PHTLIISNQDD-VSIEYLISKIEQRGISHLRVNSEDIG---SLEFSVNPVGEILC
Group-5  M------ILLVTNKRD-LTTDYIVRELKFRNVPYTRLNTETLN---EFKCSIGYSSID--
Group-6  M------ILIITHKED-YTADYIINKLNNKKISYFRLNCEDIP---KYNFSIDSS-----
Group-7  MS---KFCLVLCSKYD-FSVDLVIQELEESGVDYIRLNKEELS---DYLINIDPVKRVLA
Group-11 M-----TVLVLTCEED-VTADLVVVHLHASGVPVVRIDPADLTGGVALSGEYVHGAFRGH
Group-13 M-----TVLILTNPFD-ITADDVILRLTEHGVPVVRLDPADFPQQVVLHSEIGGNGWTGT
Group-16 MPAHRRPLILSSLAHD-IHASAARWALRASGIDATWLSSLAEA---ELGAMSLHCDPDLG
MirB     M------ILILTSPDD-VHADAAERELRRRGATWVRFDPADVPARASLSVGIRDGRLVRT
         *       ::     *                    ..

Group-1  IV----------------------------------TDGEHKLDLSEVSSVWYRRMRYGA
Group-2  VDG---------------------------------------QQIARPAAVYLRSLYQ--
Group-3  NR-----------------------------------KSNLIINIQEIHSLYYRKITL--
Group-4  T------------------------------------NQGSIFDLRFTKSVIFRRIPV--
Group-5  ---------------------------DWR-------IGGD-IKGNQITSAYFRRPSPTT
Group-6  -------------------------------------LNFNINGIDKFKSVWFRRTKLFD
Group-7  VK-----------------------------------GHGINREILNVTSVWFRQPVFIR
Group-11 LS-----------------------------------AGGRLVSVSGLRSIWVRRPGT--
Group-13 LT-----------------------------------TPHRILDLSTVTGIWYRRPRKFR
Group-16 LR-----------------------------------MSGP-LDDASVGTVWYRRPRNPE
MirB     LVRDRVPADRAPDDRTPADRVPADRPDTHRGDGDPGGAGRETVDLDAVTAVWFRRPGVPV
                                                              *

Group-1  KLPD--SMDQQFREASLKECRLTVRGLIASLP------GFHFDPMINVDRAN------NK
Group-2  -SPG--AYGVDADKAMQDNWRRTLLAFRERSTLMSAVLLRWEEAGTAVYNSPRASANITK
Group-3  --PNLDGYESKYWTLMQREMMSIVEGIAETAG------NFALTRPSVLRKAD------NK
Group-4  --KYNTGTDGENHKYLNHERKHFFEGLYLTLQ-----HAKWINPMFATQIAE------RK
Group-5  VIHP--TLSEAELEYLNMEWLSFLKSIYYRLD------NKWLSSPTNIALAE------DK
Group-6  F--K--NFSLEEQEFLINEYDSFLKNLFSIIS-----TDKWLSNPKNIYSAE------NK
Group-7  NTPG--HALALEEQLSRSQWAAFLRGLIIFEN------AIWMNWPASTYSAE------SK
Group-11 --PA--TRAPQPSAWLTEEASQALYGMLRGSG------ARWMNHPDAARRAR------HK
Group-13 L-PA--QMSQAEYEFAATEARRGFGGIINSLT-------GWINHPSAIGRAE------YK
Group-16 SFP---RAQAGDQPFLRGEWGRFLKNVYALTD--QGADRLWVNRPAAAAMAE------NK
MirB     A-PA--HLTSPYADFVVAESRQFVTDVWETLD-----VPALPAPRPVVLRAQ------HK
                           :    .  .                       :        *

Group-1  QLQLQIARQLGLLTPRTLTSNNPEAVKQFAQDCKEQGIVTKMLSQFAIYGENK-EENVVF
Group-2  PFQLALLRDAGLPVPRSLWTNDPEAVRRFHAEVGD--CIYKPVAGGAR------------
Group-3  IVQMKLAEEIGFILPQSLITNSNQAAASFCNKNNT--SIVKPLSTGRILGKNK--IGIIQ
Group-4  IFQLQTAHQLGLLTPKTIITNKLSIALDFLTKYKE--SIIKPISNGLQVIDEK--VFSIY
Group-5  PRQLVAANALGFQIPQGIITNDVFTVMERTTN--ES-LIAKPLRQALLKGKNE---SVIF
Group-6  LLQLQYAKSIGFNIPKTIISTNPNQIRDFYFSHNEK-IILKPFWNSKLFQNNK--TKLLF
Group-7  PYQLMLASKIGFKVPETMIGNDSNYFEKFDTD-----IIVKSLDTVLLRDGED--CLFTY
Group-11 PWQLRLAQRSGLPVPATLITTFPQAAREFSRRFPD--LVVKPVSGAHPQDPPR----AVP
Group-13 PYQLHHAVQAGLNVPRTLITNDPKQAKGWCARVGD--VVYKPLSAPSWLENGD--TYVVF
Group-16 LVQLRAAHHCGLRFPPTLVSHDPAEIRRFVQRHGR--VVYKPFLTHTWKDAETGRMFSTY
MirB     VRQLQLALRVGFDLPATVAGNDPDAVLDLFSHTGGR-LISKQVGLSSLGG-----DLLRF
           *:      *:  *  :                    :  * .

Group-1  TSPVTDDDLD--NLEGLSFCPMTFQENVPKALELRTIIVGKQVFTAAINSQQL-DGATYD
Group-2  TRKLEAKDLEADRIERLSAAPVCFQELLTGD-DVRVYVIDDQVICALRIVTD-----EID
Group-3  TNLVETHE----NIQGLELSPAYFQDYIPKDTEIRLTIVGNKLFGANIKSTN-----QVD
Group-4  TTKVDYTLFSNLNLSPIFDTPAFLQERVSNAADIRVTIIGEVGYAVKILKEG----DETD
```

```
Group-5   TSQIPSLTPE--YEESIAFAPSIYQQEISKKYDVRVTVVGNKVFPVAIWSQTS-EATKVD
Group-6   TNLVDKKHID--DLDNMEITPIIFQEYIEKKIEYRITVVEDEIFVASVDSQKN-EKTKID
Group-7   TSSITSKEL---NEDNTKIAPLTVQEYVYPKTDIRVTVIGEKVHAVRITSAG--SGIDED
Group-11  TSRVAPDT----DFAAVAFGPTLLQRRVAKRADIRLTVVGDRMLAARTATAPDPDPAEVD
Group-13  TTPITPDQW---GDPAIGRTAHMFQQRLDKEFEVRLTMVDGKAFPAAIHAHS--DAARID
Group-16  ARIVEPAMLE--DDQSLLHCPGIYQVYVDKRHDLRVTAIGDRYFAARLYAQH--DDGFVD
MirB      TEPVTHRDIA--HVDGVRHCPFIAQARVPKQVELRVTVVDGRVFTAAIDSQRS-NHARHD
          :  :                .   *  :    : *   :                    *

Group-1   WRKEGRALHEKWQAYNLPEDIEKKLLQLTAYFGLNYGAIDIIVTPDGRHVFLEINPVGEF
Group-2   FRQAE----ERIEAIEISDEVKDQCVRAAKLVGLRYTGMDIKAGADGNYRVLELNASAMF
Group-3   WRKNDAL--LEYKPANIPDKIAKMCLEMMEKLEINFAAFDFIIR-NGDYIFLELNANGQW
Group-4   WRKPEIV--KKYELTTLPENLMKRLFNLNKHLGLVYSAIDLILTPSGDYVFLEVNPVGEW
Group-5   WRQGGRID-LLHEHIDIPDELKMKCINLVKDLNLRFGAIDLVCDLDGDYWFLEINPNGQW
Group-6   WRRDN----SKFQKDKIPKEIENKCLELVKMLGLKFGAIDLIQDEYDNFYFLEINANGQW
Group-7   WRLLKKEE-IEYTDVKLPPVIVGYCREIVREMGLRFGAIDLIES-NEIYYFIEINPTGEW
Group-11  VRFAVSA--QPWRPTEVPPRIAEAVLTYMRDAELAYGAFDFAEDGDGTWWFLECNQSGQF
Group-13  WRSDYDA--LTYSIPTVPQRVLTGARDLLRRLHLRYAALDFIVSPDGRWHFLEVNPNGQY
Group-16  WRVPQNTQGLRAEVGELSSADQAKLAALMRELGLVFGCIDLAIDANGDAHFLEVNQAGQF
MirB      WRRQDGRN-TPMRPYPLPDDVAARCVALTRRLGLRYGALDLIVTPDGRYVFIEINPTGQY
           *              :.               : :  :*:         .:* *  . :

Group-1   FWLELFAPYFPISQAIAEILLTPTNQ---------------------------------K
Group-2   RGFEGRAN-VDICGPLCDALIAQTKR----------------------------------
Group-3   LWLEDILK-FDISNTIINYLLG-------------------------------------E
Group-4   VWLELELG-ICISEQILKELL---------------------------------------
Group-5   AWIENQTK-LPIASSIVDELLKISEN---------NYES---------------------
Group-6   VWIESDTG-LTISDSIINFLNA--------------------------------------
Group-7   GWLSTEN--RPIEKDIANMLAG--------------------------------------
Group-11  GFVEAETG-QPIARTIAEWLALPAAP-------------------EREGLVNGADSTAG-
Group-13  GWIEEHTG-QPISDAIADALTRKEN-----------------------------------
Group-16  LFIEDLLPSLPLLRAMSAMLAEGRPDYSLATIAKLSYAEYCASEEHREWWAQASETLKGR
MirB      LWVEEETG-LPITAAVVDLLTTAAPA-------------------------------PGD
            ..       :   :   *
```

Fig. S31 Sequence alignment of graspetide synthetases. Multiple sequence alignment of representative graspetide synthetases from characterized groups. The NCBI accession IDs for the graspetide synthetases are as follows: Group-1: WP_042156020.1; Group-2: WP_006971586.1; Group-3: WP_000849148.1; Group-4: OJW02008.1; Group-5: GAJ78971.1; Group-6: WP_059136627.1; Group-7: CUB04732.1; Group-11: WP_019056921.1; Group-13: WP_193587235.1; Group-16: WP_057916645.1; MirB (Group-21): WP_067373627.1. ATP-binding residues are red, and residues posited to be critical for acceptor residue binding and enzyme activity are cyan. Minor trimming of a few residues from the highly divergent C-termini was performed.

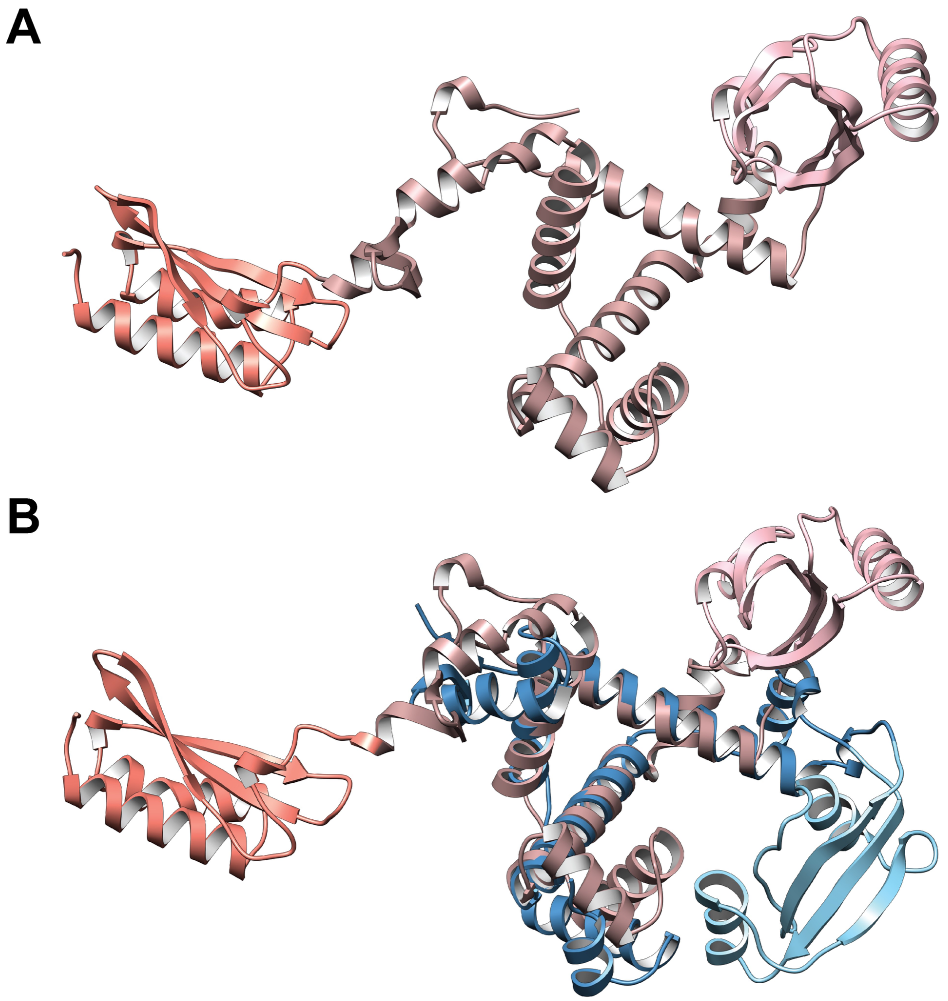

Fig. S32. MirC structural predictions. A. AlphaFold predicted structure for MirC (UniProt ID: A0A136PIX8 (from AlphaFold BD), pLDDT: 86.62, pTM: 0.69). N-terminal domain (residues 1-90) is salmon and C-terminal SurA-like domain is rosy brown (residues 105-214, 288-315) and pink (residues 215-287). B. Structural alignment of MirC AlphaFold structure with Cje1289 (PDB: 3RGC). The chaperone and PPIase domains of Cje1289 are dark and light blue, respectively. MirC shares structural similarity with the chaperone domain (RMSD: 4.87) but not the PPIase domain of Cje1289.

MirA: SGSMKRKPVQIDTIPADGVELADDQLATMLGGLPRNGAGSSKTIDDIPGGGRPDTDSDF
M+H: 6039.97 Da

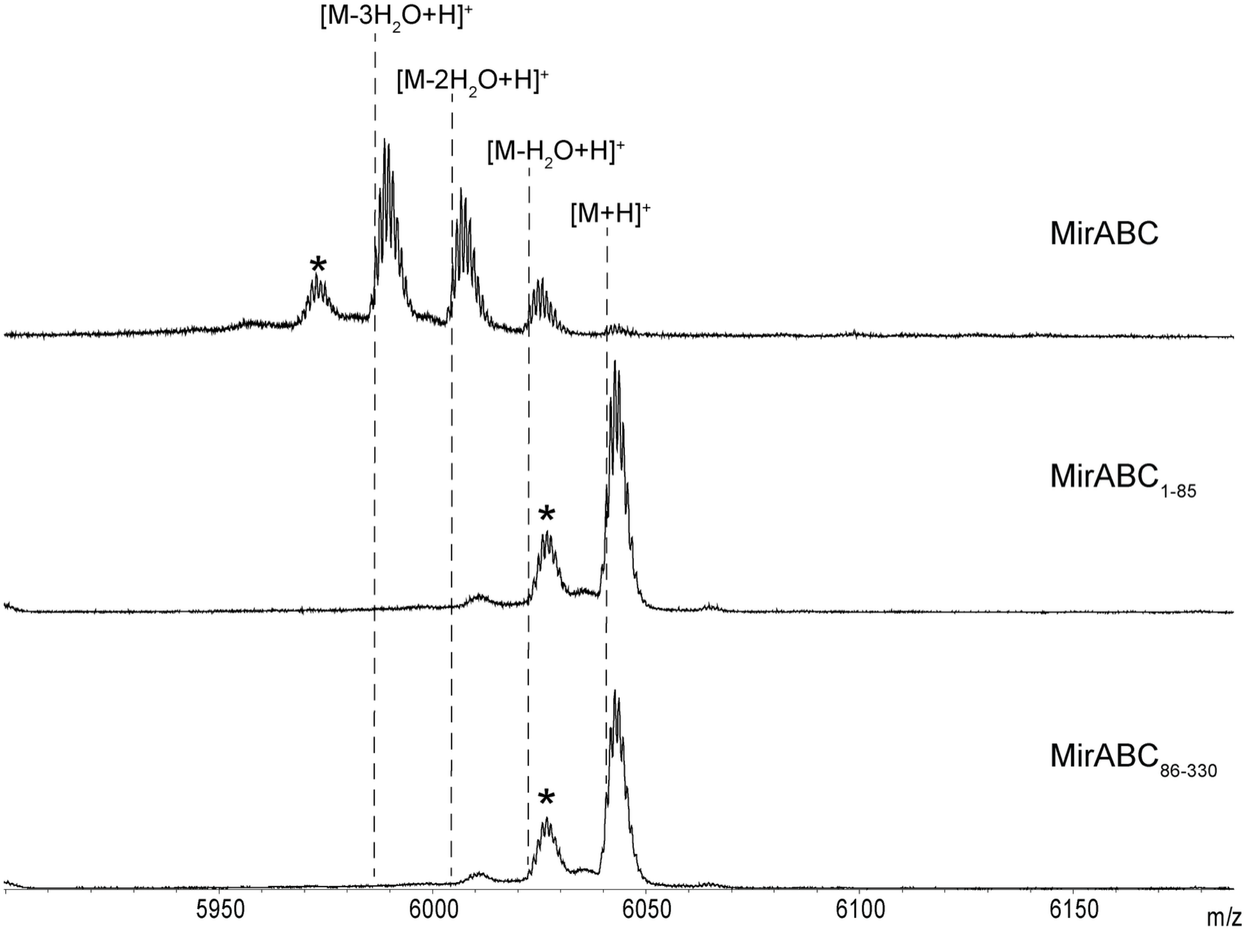

Fig. S33. MALDI-TOF MS of MirA co-expressed with MirB and MirC truncates. MALDI-TOF mass spectra of MirA co-expressed with MirB and full-length MirC (*top*), or MirC N-domain (*middle*) or MirC C-domain (*bottom*). Asterisks denote peaks from laser induced deamination.

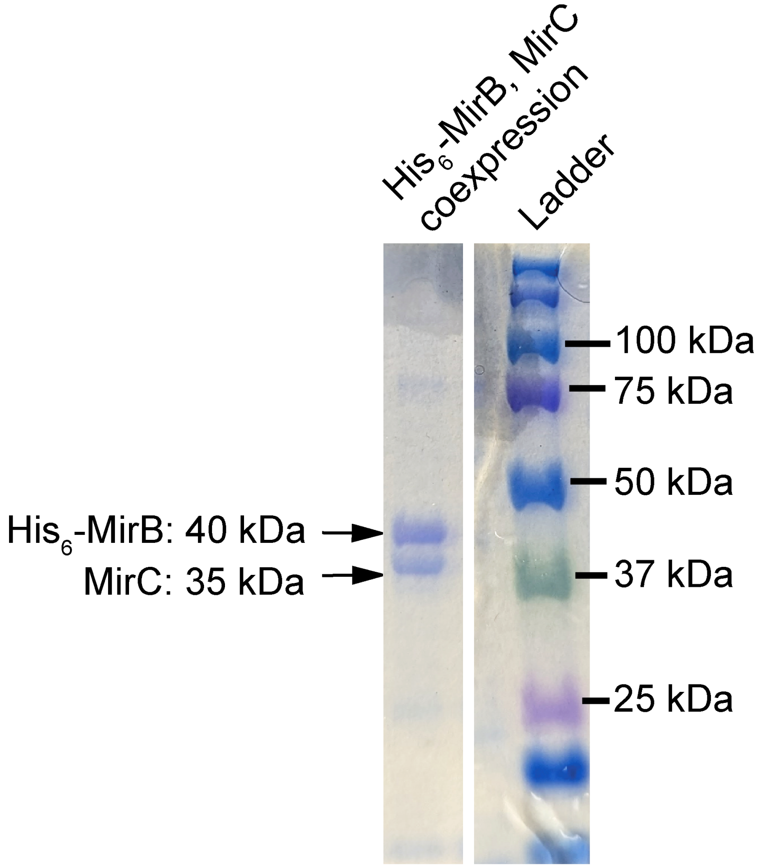

Fig. S34. His_6-MirB and MirC co-purification. A) SDS-PAGE analysis of His_6-tagged MirB co-purified with untagged MirC.

A

MirB : 89.2% trypsin coverage

```
        10         20         30         40         50         60
MGSSHHHHHH SSMILILTSP DDVHADAAER elrrrGATWV RFDPADVPAR SLSVGIRdg
        70         80         90        100        110        120
rlvrtlvrdr VPADRAPDDR TPADRVPADR PDTHRGDGDP GGAGRETVDL AVTAVWFRR
       130        140        150        160        170        180
PGVPVAPAHL TSPYADFVVA ESRQFVTDVW ETLDVPALPA PRPVVLRaqh vrQLQLALR
       190        200        210        220        230        240
VGFDLPATVA GNDPDAVLDL FSHTGGRlis kQVGLSSLGG DLLRFTEPVT RDIAHVDGV
       250        260        270        280        290        300
RHCPFIAQAR vpkQVELRVT VVDGRVFTAA IDSQRSNHAR HDWRrqdgrN PMRPYPLPD
       310        320        330        340        350        360
DVAARCVALT RrlglrYGAL DLIVTPDGRY VFIEINPTGQ YLWVEEETGL ITAAVVDLL
       370
TTAAPAPGDP A
```

B

[M+H]+	Position	peptide sequence	Observed?
4442.2693	330-371	YVFIEINPTGQYLWVEEETGLPITAAVVDLLTTAAPAPGD PA	N/A
3325.5337	1-30	MGSSHHHHHHSSMILILTSPDDVHADAAER	N/A
2741.3528	181-207	VGFDLPATVAGNDPDAVLDLFSHTGGR	Yes
2718.4977	144-167	QFVTDVWETLDVPALPAPRPVVLR	No
2536.3306	120-143	RPGVPVAPAHLTSPYADFVVAESR	Yes
1812.9061	290-305	NTPMRPYPLPDDVAAR	Yes
1621.8220	106-119	ETVDLDAVTAVWFR	Yes
1389.7372	317-329	YGALDLIVTPDGR	Yes
1314.7375	212-224	QVGLSSLGGDLLR	Yes
1163.5916	86-95	VPADRPDTHR	Yes
1107.5793	266-275	VFTAAIDSQR	Yes
1042.5251	242-250	HCPFIAQAR	No
987.4894	42-50	FDPADVPAR	No
986.5054	225-232	FTEPVTHR	Yes
981.5112	233-241	DIAHVDGVR	Yes
858.3700	96-105	GDGDPGGAGR	No
841.5254	174-180	QLQLALR	Yes
802.4781	51-58	ASLSVGIR	Yes
745.4202	259-265	VTVVDGR	N/A
689.3729	36-41	GATWVR	N/A
662.3654	306-311	CVALTR	N/A
644.3726	254-258	QVELR	N/A
613.2841	281-284	HDWR	N/A
584.2899	276-280	SNHAR	N/A
573.2627	76-80	APDDR	N/A
559.2834	81-85	TPADR	N/A
557.3042	71-75	VPADR	N/A

Total: 212/371 unobserved; 159/371 observed; 42.9% sequence coverage

Fig. S35. In-gel trypsin digestion of His$_6$-MirB. SDS-PAGE gel band corresponding to MirB in Fig. S34 was excised and treated with proteomics-grade trypsin. A) sequence of MirB, residues in red are in observable tryptic fragments >500 Da. B) sequence of each tryptic fragment, along with whether that fragment was observed.

A

MirC : 92.1% trypsin coverage

```
        10         20         30         40         50         60
MTILDPALLD AAVALARELS TARatlrDAT EQLAPLRarW PQADPALVRD EGSDGSVSF
        70         80         90        100        110        120
DVLLREPTGT VSVAYSSAPA LPWPLRgalr HSEHHLARvg prTLLVGEAL ALDFLWYDH
       130        140        150        160        170        180
DVLVRLVDTS LIRAELERCP IPVSDADLQA AADAYRrakG LLDAASTARW AERGLSEVD
       190        200        210        220        230        240
FTELVADTVA VARlrekIVG DRverWFEAH RSAFDTLVVA WAADGPLPAD DDALAAVAT
       250        260        270        280        290        300
AVRIGHAAGV LRVVAAEAAP ELRAATGPVP TVVAGAPVSA LPVGREPAVL AATRSLVER
       310        320        330
AIFDDWLAQA RVAVDIEWFW LPRdrTAPLT
```

B

$[M+H]^+$	Position	Peptide sequence	Observed?
3166.6054	212-243	SAFDTLVVAWAADGPLPADPDDALAAVATAVR	Yes
2629.4024	103-125	TLLVGEALAALDFLWYDHDVLVR	Yes
2199.1444	66-86	EPTGTVSVAYSSAPALPWPLR	Yes
1992.0283	175-193	GLSEVDFTELVADTVAVAR	Yes
1987.1334	264-285	AATGPVPTVVAGAPVSALPVGR	Yes
1875.8905	139-156	CPIPVSDADLQAAADAYR	Yes
1753.9880	1-17	MTILDPALLDAAVALAR	No
1724.7973	50-65	DEEGSDGSVSFDVLLR	Yes
1530.8103	312-323	VAVDIEWFWLPR	Yes
1305.6586	301-311	AIFDDWLAQAR	Yes
1152.6160	40-49	WPQADPALVR	Yes
1125.6262	253-263	VVAAEAAPELR	Yes
1113.5898	28-37	DATEQLAPLR	Yes
1041.5687	286-295	EPAVLNAATR	Yes
986.4915	91-98	HSEHHLAR	Yes
974.5265	160-169	GLLDAASTAR	Yes
916.5462	126-133	LVDTSLIR	Yes
893.5315	244-252	IGHAAGVLR	Yes
845.4053	206-211	WFEAHR	Yes
676.3624	18-23	ELSTAR	N/A
674.3620	170-174	WLAER	N/A
617.3253	134-138	AELER	N/A
603.3460	296-300	SLVER	N/A
559.3198	198-202	IVGDR	N/A
502.2871	326-330	TAPLT	N/A

Total: 74/330 unobserved; 256/330 observed; 77.6% sequence coverage

Fig. S36. In-gel trypsin digestion of MirC. MirC was pulled down upon IMAC purification of His_6-MirB. SDS-PAGE gel band corresponding to MirC in Fig. S34 was excised and trypsinized. A) sequence of MirC, residues in red are in observable tryptic fragments >500 Da. B) sequence of each tryptic fragment, along with whether that fragment was observed.

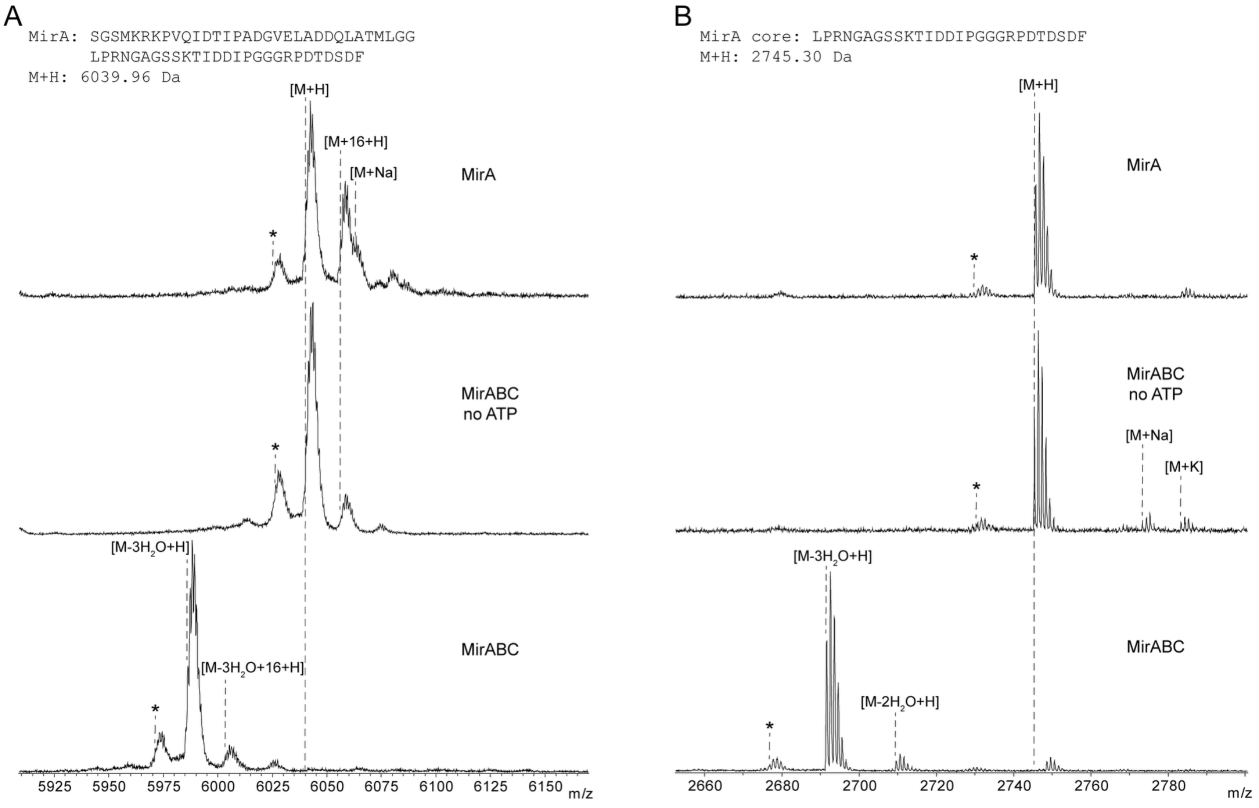

Fig. S37. *In vitro* reconstitution of MirBC activity. A) MALDI-TOF mass spectra following in vitro reconstitution of graspetide synthetase activity using the MirBC complex and MirA. B) Modified MirA core peptide after leader peptide removal using $LahT_{150}$ protease [5].

CorA:MHHHHHHAMTQIKSPTGFISSLKTALKTASTDGKavgapalkkALQGADVGKLTPQQK

M_{2}:MHHHHHHAMTQIK M_{5}:ALQGADVGKLTPQQK

M_{3}:SPTGFISSLK

M_{19}:SPTGFISSLKTALKTASTDGK

CorA cont:NQLRDAFEQVKltpkARELADSFVKGKNDSLKNPDDGVIVKIVADDPLR

M_{6}:NQLRDAFEQVK M_{8}:ARELADSFVK

M_{9}:GKNDSLKNPDDGVIVKIVADDPLR

CorA cont:GGGTTGGTSFKPPADSGAVKVMMDDPTRGSKLPDFTPPDGGR

M_{10}:GGGTTGGTSFKPPADSGAVKVMMDDPTR

M_{11}:GSKLPDFTPPDGGR

CorA cont:VKIRMDDPTGGLKLPDFTPPKDGGLVKVMMDDPTRGSKLPDLKPLDGGIVKVKMDSPV

M_{12}:VKIRMDDPTGGLKLPDFTPPK M_{1}:GSKLPDLKPLDGGIVK

M_{4}:DGGLVKVMMDDPTR

M_{13}:VKIRMDDPTGGLKLPDFTPPKDGGLVK M_{7}:LPDLKPLDGGIVK

M_{14}:MDDPTGGLKLPDFTPPKDGGLVKVMMDDPTR

M_{15}:MDDPTGGLKLPDFTPPKDGGLVK M_{20}:LPDLKPLDGGIVKVKMDSPV

M_{16}:LPDFTPPKDGGLVKVMMDDPTR

M_{17}:VKIRMDDPTGGLKLPDFTPPKDGGLVKVMMDDPTR

M_{18}:IRMDDPTGGLKLPDFTPPK

Fig. S38. CorA tryptic fragments coverage. The sequence for His_6-tagged CorA overlaid with the labeled mass fragments identified in tryptic digests. Sequences not covered by tryptic digest are shown in lowercase letters. The proposed sites of modifications on peptide fragments with observed modifications are underlined. The fragment labels are consistent with mass labels found in figure S39.

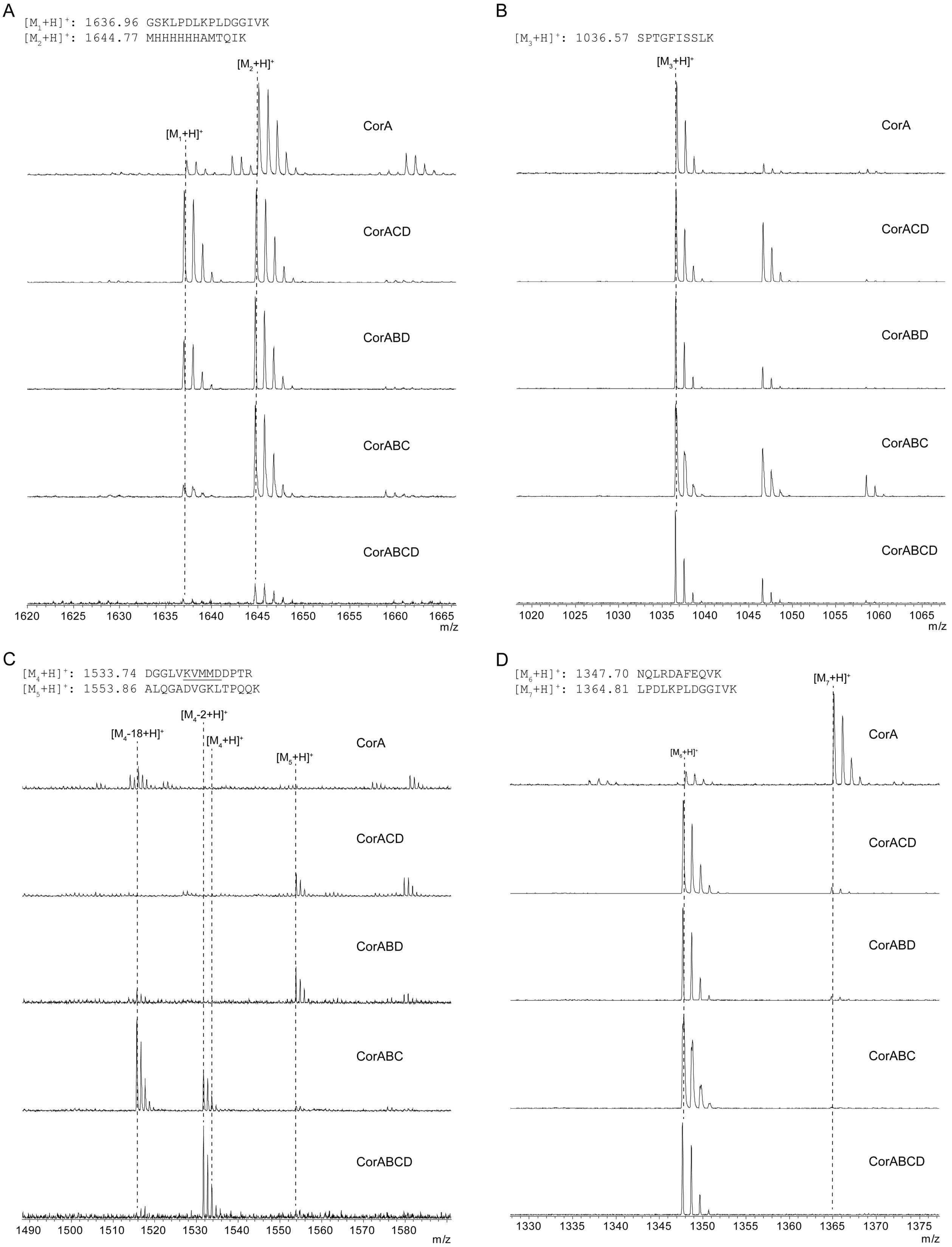
A
[M1+H]+: 1636.96 GSKLPDLKPLDGGIVK
[M2+H]+: 1644.77 MHHHHHHAMTQIK
[M2+H]+
[M1+H]+
CorA
CorACD
CorABD
CorABC
CorABCD
1620 1625 1630 1635 1640 1645 1650 1655 1660 1665
m/z
B
[M3+H]+: 1036.57 SPTGFISSLK
[M3+H]+
CorA
CorACD
CorABD
CorABC
CorABCD
1020 1025 1030 1035 1040 1045 1050 1055 1060 1065
m/z
C
[M4+H]+: 1533.74 DGGLVKVMMDDPTR
[M5+H]+: 1553.86 ALQGADVGKLTPQQK
[M4-2+H]+
[M4-18+H]+
[M4+H]+
[M5+H]+
CorA
CorACD
CorABD
CorABC
CorABCD
1490 1500 1510 1520 1530 1540 1550 1560 1570 1580
m/z
D
[M6+H]+: 1347.70 NQLRDAFEQVK
[M7+H]+: 1364.81 LPDLKPLDGGIVK
[M7+H]+
[M6+H]+
CorA
CorACD
CorABD
CorABC
CorABCD
1330 1335 1340 1345 1350 1355 1360 1365 1370 1375
m/z

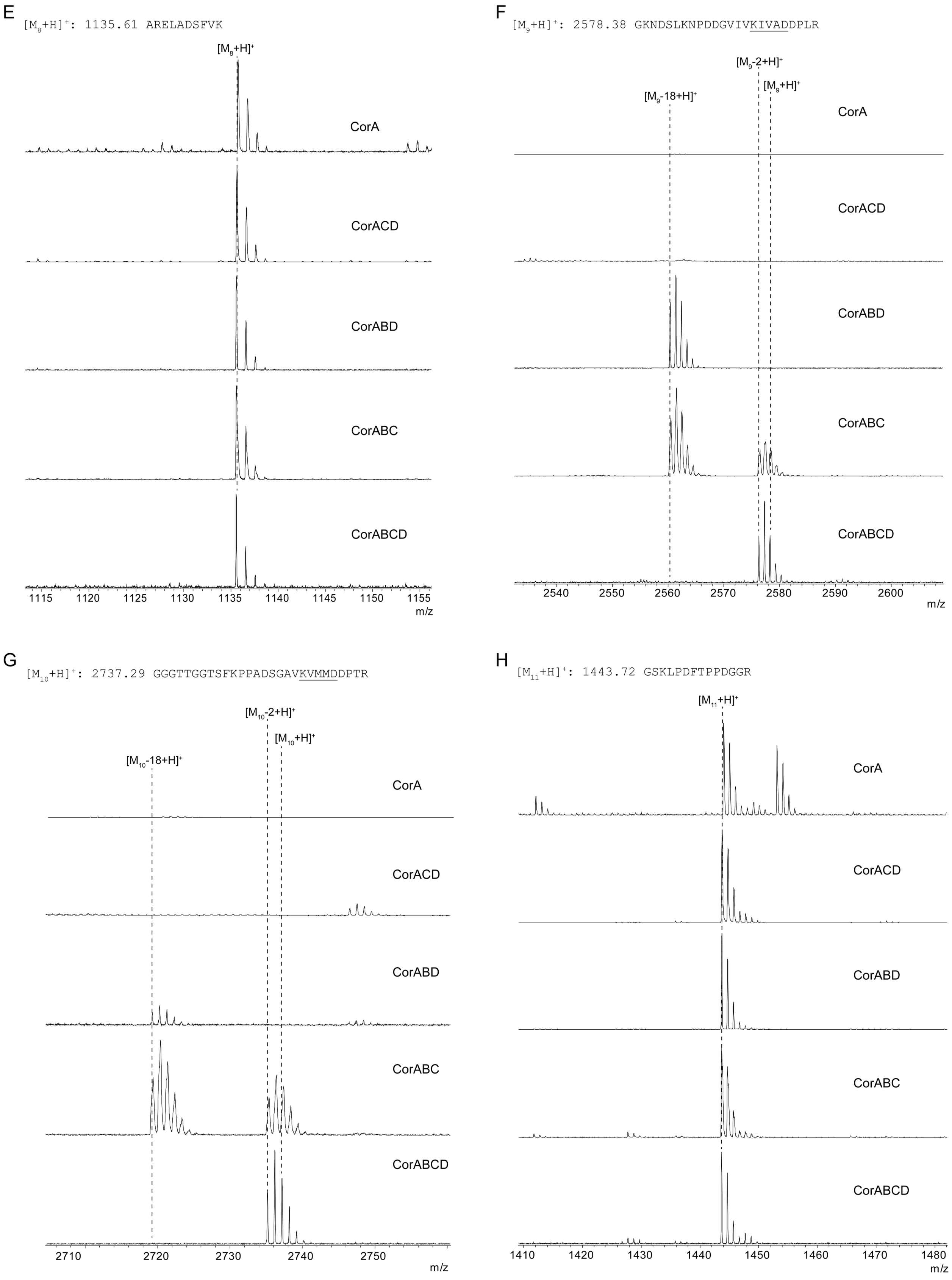
E
[M8+H]+: 1135.61 ARELADSFVK
[M8+H]+
CorA
CorACD
CorABD
CorABC
CorABCD
1115 1120 1125 1130 1135 1140 1145 1150 1155
m/z
F
[M9+H]+: 2578.38 GKNDSLKNPDDGVIVKIVADDPLR
[M9-2+H]+
[M9+H]+
[M9-18+H]+
CorA
CorACD
CorABD
CorABC
CorABCD
2540 2550 2560 2570 2580 2590 2600
m/z
G
[M10+H]+: 2737.29 GGGTTGGTSFKPPADSGAVKVMMDDPTR
[M10-2+H]+
[M10+H]+
[M10-18+H]+
CorA
CorACD
CorABD
CorABC
CorABCD
2710 2720 2730 2740 2750
m/z
H
[M11+H]+: 1443.72 GSKLPDFTPPDGGR
[M11+H]+
CorA
CorACD
CorABD
CorABC
CorABCD
1410 1420 1430 1440 1450 1460 1470 1480
m/z

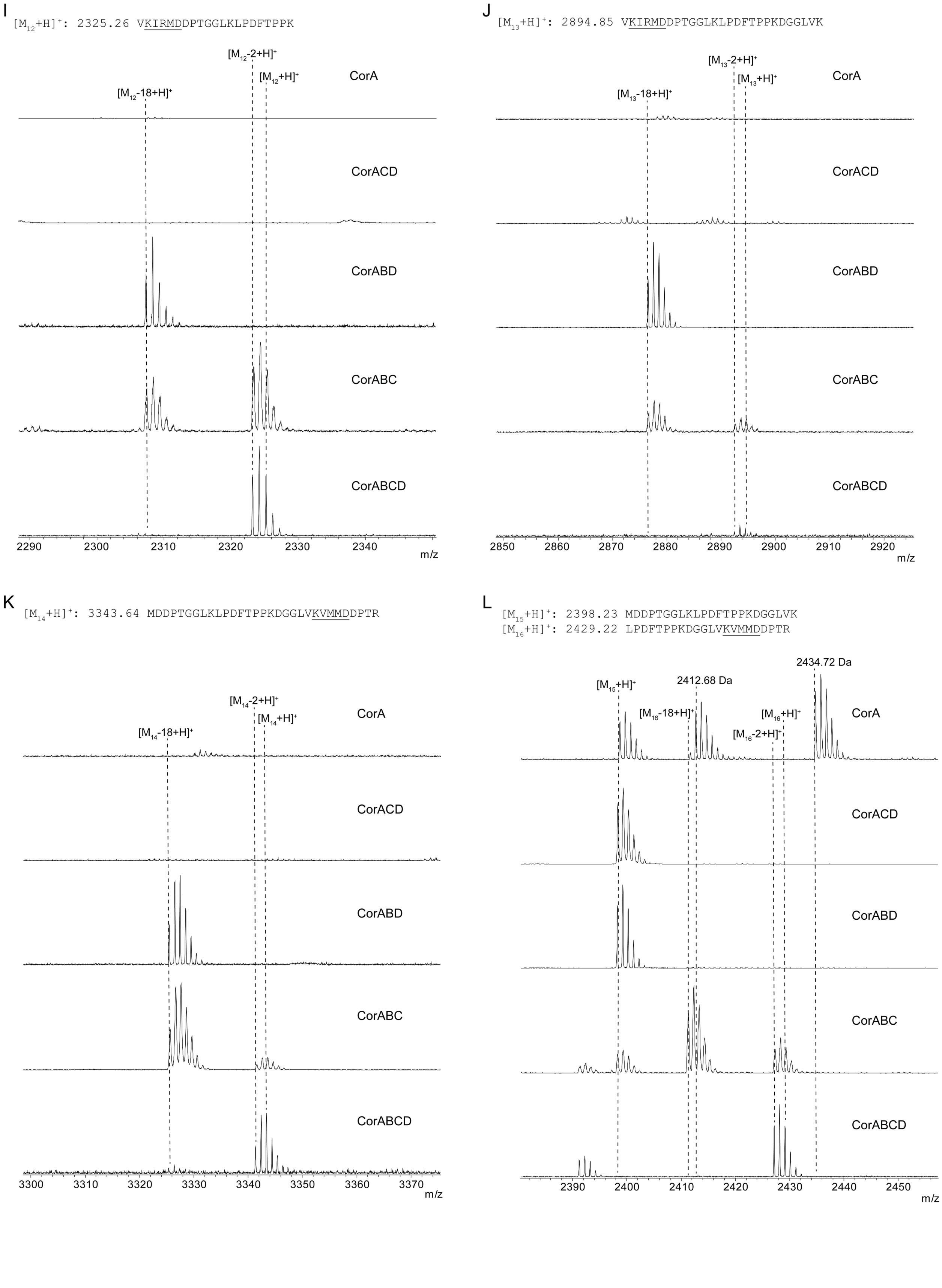
I
[M12+H]+: 2325.26 VKIRMDDPTGGLKLPDFTPPK
[M12-2+H]+
[M12+H]+
[M12-18+H]+
CorA
CorACD
CorABD
CorABC
CorABCD
2290 2300 2310 2320 2330 2340
m/z
J
[M13+H]+: 2894.85 VKIRMDDPTGGLKLPDFTPPKDGGLVK
[M13-2+H]+
[M13+H]+
[M13-18+H]+
CorA
CorACD
CorABD
CorABC
CorABCD
2850 2860 2870 2880 2890 2900 2910 2920
m/z
K
[M14+H]+: 3343.64 MDDPTGGLKLPDFTPPKDGGLVKVMMDDPTR
[M14-2+H]+
[M14+H]+
[M14-18+H]+
CorA
CorACD
CorABD
CorABC
CorABCD
3300 3310 3320 3330 3340 3350 3360 3370
m/z
L
[M15+H]+: 2398.23 MDDPTGGLKLPDFTPPKDGGLVK
[M16+H]+: 2429.22 LPDFTPPKDGGLVKVMMDDPTR
2434.72 Da
2412.68 Da
[M15+H]+
[M16-18+H]+
[M16+H]+
[M16-2+H]+
CorA
CorACD
CorABD
CorABC
CorABCD
2390 2400 2410 2420 2430 2440 2450
m/z

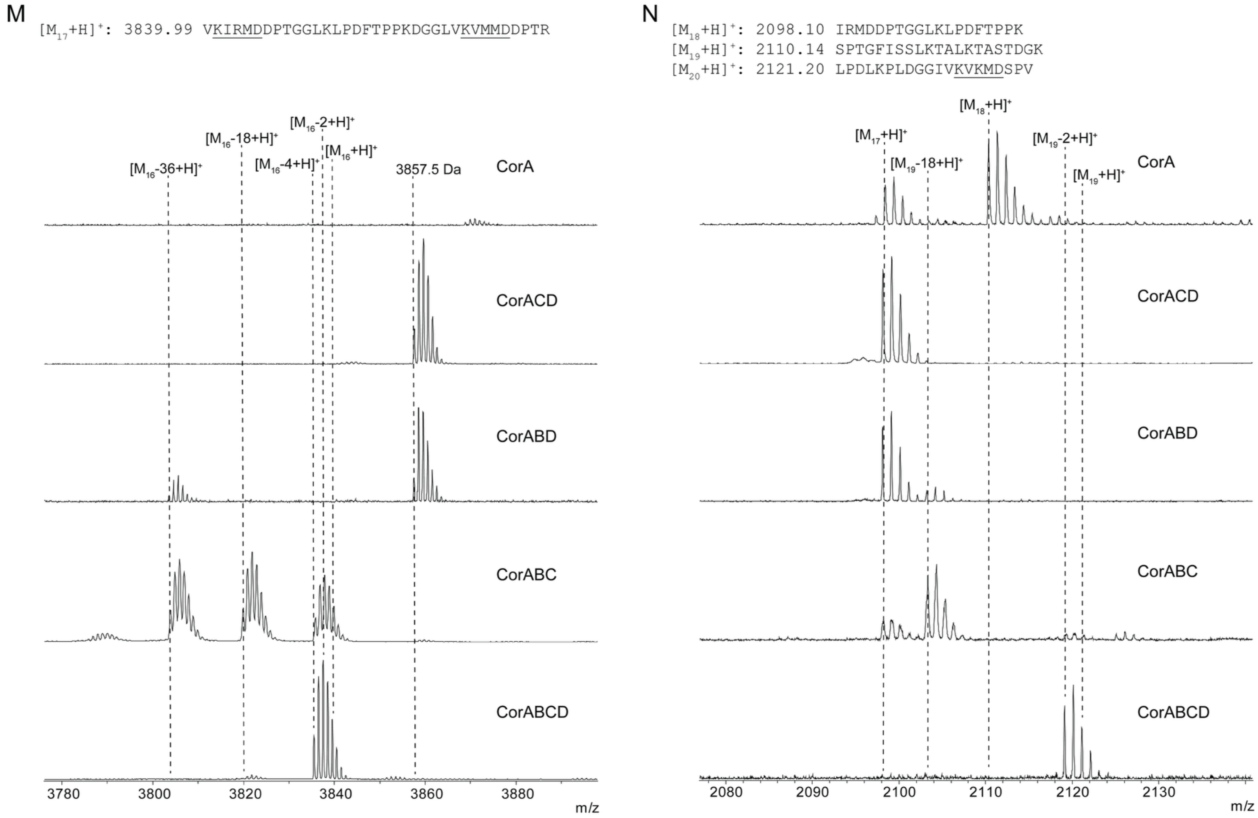

Fig. S39 (previous four pages). CorA tryptic fragments. MALDI-TOF mass spectra of His$_6$-tagged CorA heterologously expressed with various combinations of biosynthetic enzymes after trypsin digestion. Panels C, F, G, I, J, K, L, M, and N contain modified fragments, with the proposed sites of modifications underlined. An overlay of the fragments found in each panel to the full length CorA sequence can be found in Figure S38.

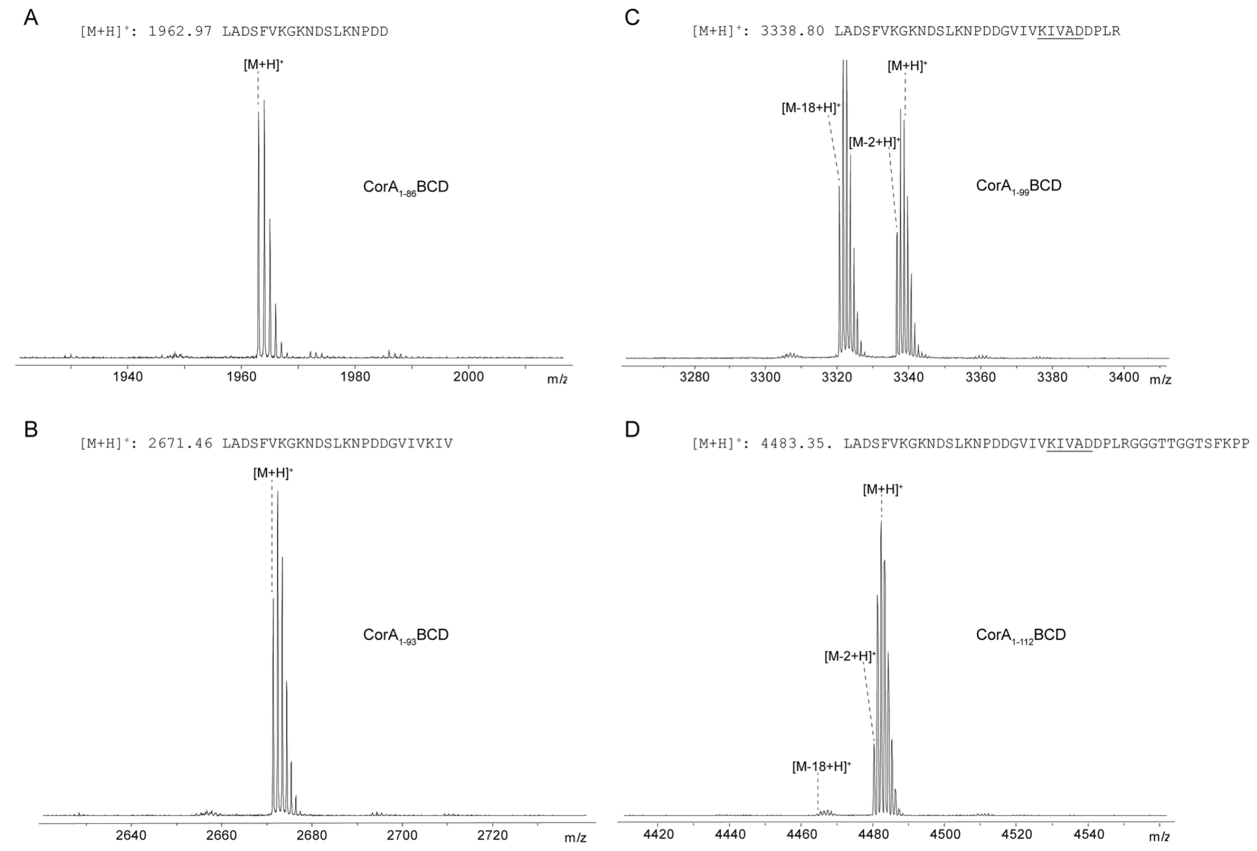

Fig. S40. Heterologous expression of CorA truncate fragments. MALDI-TOF mass spectra of His_6-tagged CorA truncates heterologously expressed with CorBCD and digested with the protease GluC. In addition to the product with a 2 Da loss containing a dehydration and oxygenation, $CorA_{1-99}$ contains a buildup of the dehydrated intermediate. In comparison, $CorA_{1-112}$ does not show buildup of the dehydrated intermediate.

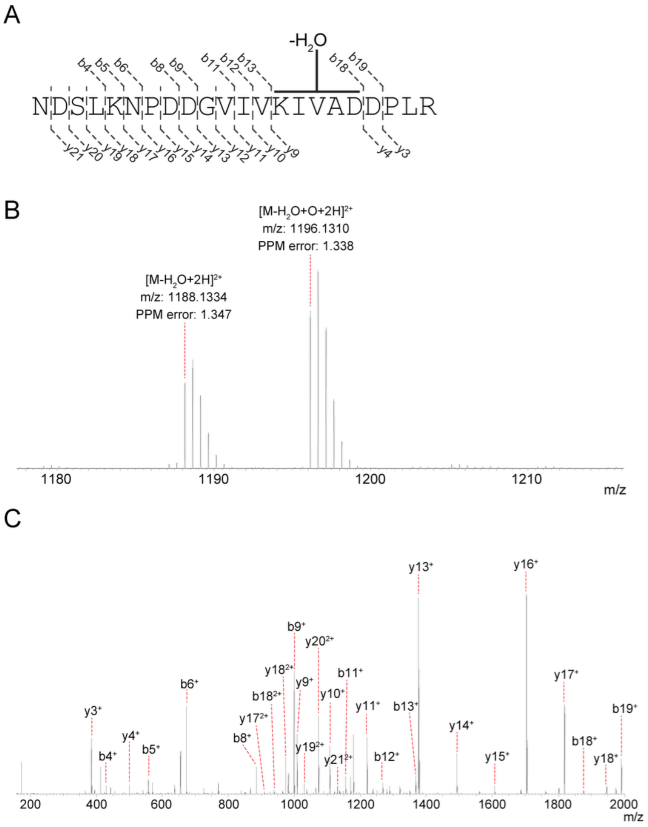

D

Ion	Calc. Mass	Obs. Mass	PPM Error
[M+2H]$^+$	1188.1318	1188.1334	1.347
b4$^+$	430.1932	430.1939	1.627
b5$^+$	558.2882	558.2891	1.612
b6$^+$	672.3311	672.3322	1.636
b8$^+$	884.4108	884.4123	1.696
b9$^+$	999.4378	999.4390	1.201
b11$^+$	1155.5276	1155.5293	1.471
b12$^+$	1268.6117	1268.6141	1.892
b13$^+$	1367.6801	1367.6823	1.609
b18$^+$	1875.9809	1875.9814	0.267
b18^{+2}	938.4941	938.4960	2.025
b19$^+$	1991.0078	1991.0087	0.452

E

Ion	Calc. Mass	Obs. Mass	PPM Error
[M+2H]$^+$	1188.1318	1188.1334	1.347
y3$^+$	385.2558	385.2564	1.557
y4$^+$	500.2827	500.2835	1.599
y9$^+$	1008.5835	1008.5849	1.388
y10$^+$	1107.6519	1107.6533	1.264
y11$^+$	1220.7360	1220.7384	1.966
y12$^+$	1319.8044	1319.8070	1.970
y13$^+$	1376.8259	1376.8281	1.598
y14$^+$	1491.8528	1491.8551	1.542
y15$^+$	1606.8797	1606.8821	1.494
y16$^+$	1703.9325	1703.9341	0.939
y17$^+$	1817.9754	1817.9773	1.045
y18$^+$	1946.0704	1946.0717	0.668
y17^{+2}	909.4914	909.4921	0.770
y18^{+2}	973.5388	973.5401	1.335
y19^{+2}	1030.0809	1030.0820	1.068
y20^{+2}	1073.5969	1073.5980	1.025
y21^{+2}	1131.1103	1131.1128	2.210

Fig. S41. HRMS/MS of the dehydrated CorA$_{1-99}$ product. A) Fragmentation of the dehydrated CorA$_{1-99}$ product. Observed b- and y-ions are annotated, with a dehydration being localized to the five residues including and between Lys91 and Asp95. B) HRMS for the CorA$_{1-99}$ products, giving the exact masses as a loss of water, and a loss of two hydrogens for the respective products. C) Annotated CID spectrum of the dehydrated CorA$_{1-99}$ intermediate. D) Table of daughter b ions. E) Table of daughter y ions.

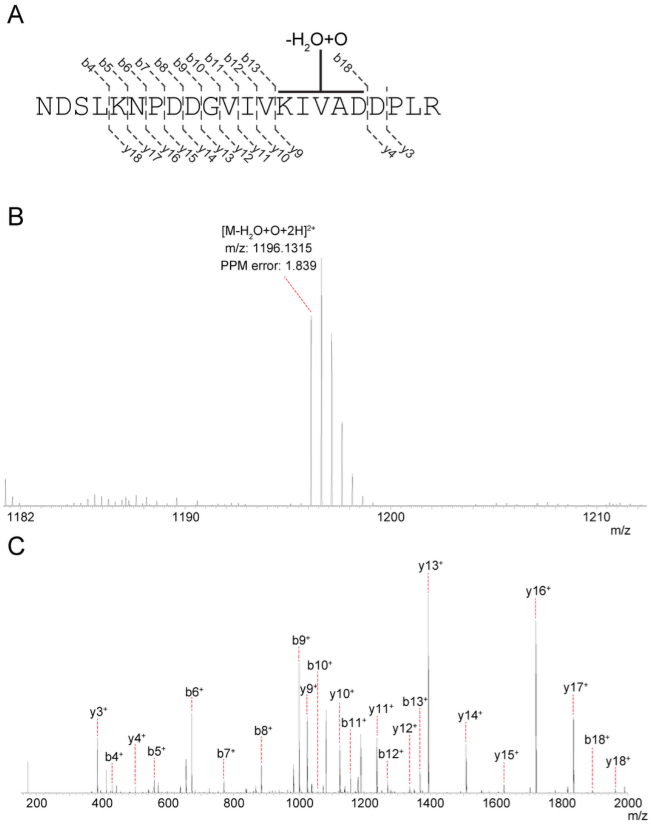

D

Ion	Calc. Mass	Obs. Mass	PPM Error
[M+2H]$^{+}$	1196.1294	1196.1310	1.338
b4^{+}	430.1932	430.1938	1.395
b5^{+}	558.2882	558.2889	1.254
b6^{+}	672.3311	672.3320	1.339
b7^{+}	769.3839	769.3848	1.170
b8^{+}	884.4108	884.4120	1.357
b9^{+}	999.4378	999.4387	0.901
b10^{+}	1056.4592	1056.4598	0.568
b11^{+}	1155.5276	1155.5289	1.125
b12^{+}	1268.6117	1268.6136	1.498
b13^{+}	1367.6801	1367.6821	1.462
b18^{+}	1891.9758	1891.9780	1.163

E

Ion	Calc. Mass	Obs. Mass	PPM Error
[M+2H]$^{+}$	1196.1294	1196.1310	1.338
y3^{+}	385.2558	385.2563	1.298
y4^{+}	500.2827	500.2834	1.399
y9^{+}	1024.5784	1024.5795	1.074
y10^{+}	1123.6468	1123.6479	0.979
y11^{+}	1236.7309	1236.7329	1.617
y12^{+}	1335.7993	1335.8016	1.722
y13^{+}	1392.8208	1392.8226	1.292
y14^{+}	1507.8477	1507.8500	1.525
y15^{+}	1622.8746	1622.8766	1.232
y16^{+}	1719.9274	1719.9287	0.756
y17^{+}	1833.9703	1833.9716	0.709
y18^{+}	1962.0653	1962.0665	0.612

Fig. S42. HRMS/MS of the dehydrated and oxygenated CorA$_{1\text{-}112}$ product. A) Fragmentation of the dehydrated and oxygenated CorA$_{1\text{-}112}$ product. Observed b- and y-ions are annotated, with both modifications being localized to the five residues including and between Lys91 and Asp96. B) HRMS for the CorA$_{1\text{-}112}$ products, giving the exact masses as a loss of two hydrogens. C) Annotated CID spectrum of the dehydrated and oxygenated CorA$_{1\text{-}112}$ product. D) Table of daughter b ions. E) Table of daughter y ions.

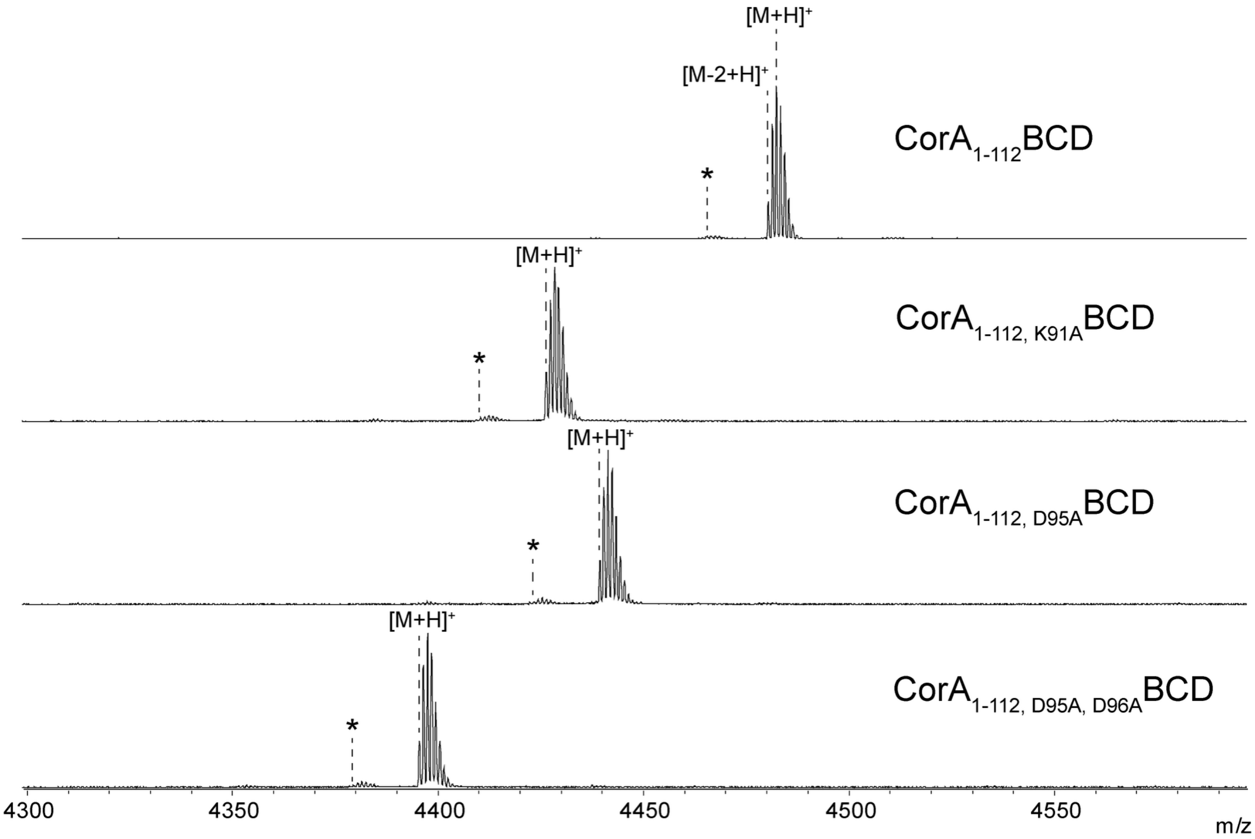

Fig. S43. CorA$_{1\text{-}112}$ acceptor-donor variants. MALDI-TOF mass spectra of CorA$_{1\text{-}112}$ acceptor and donor residue variants after co-expression with CorBCD and digestion with GluC. Alanine variants of either of these residues, or neighboring identical residues, resulted in no observable modifications when expressed with CorBCD.

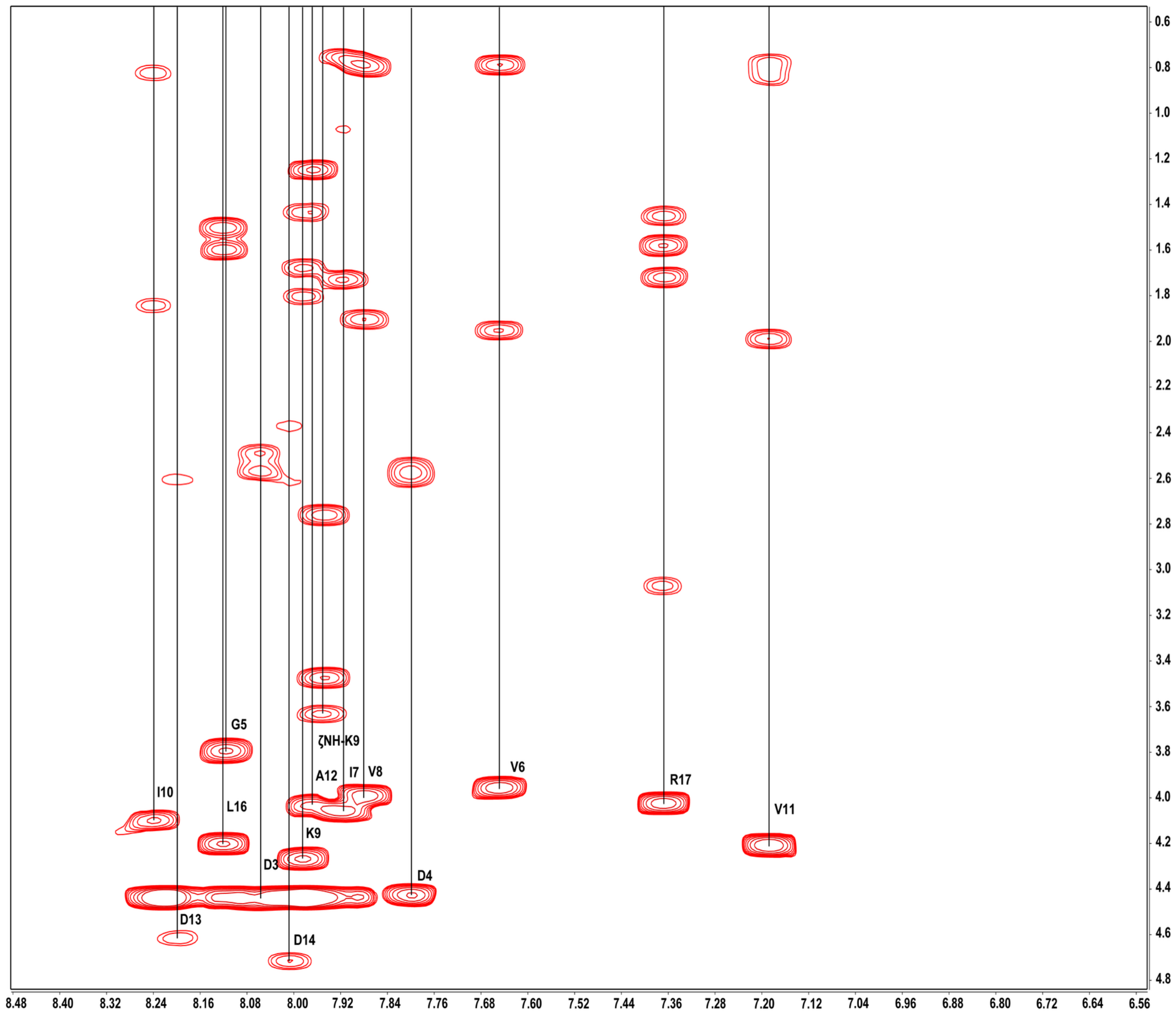

Fig. S44. ^{1}H-^{1}H TOCSY correlations of a corallotide fragment. TOCSY correlations are labeled by each amino acid spin system.

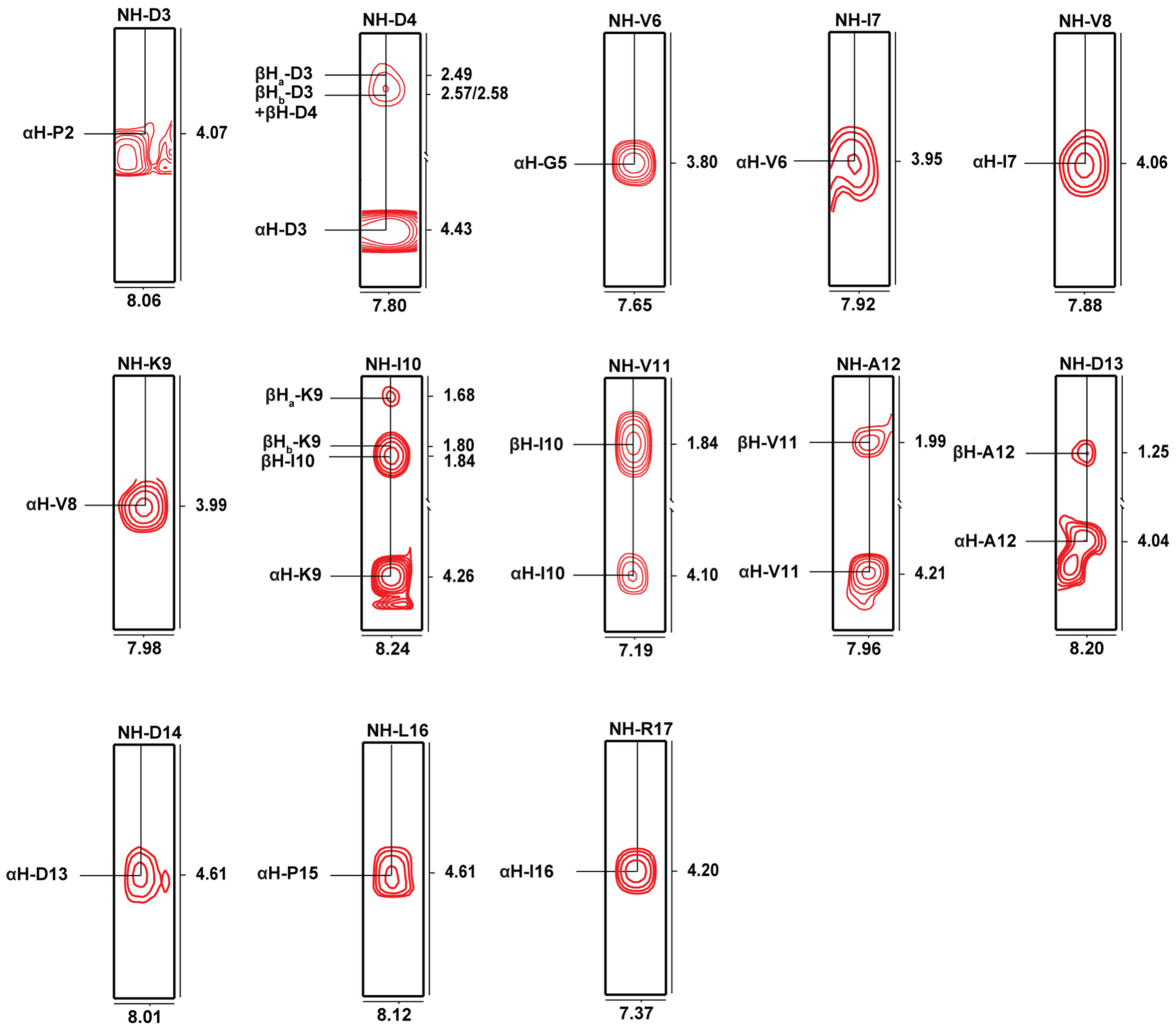

Fig. S45. ^{1}H-^{1}H NOESY correlations of corallotide fragment. NOESY correlations are labeled that were used to assign the linear sequence of amino acids in the modified corallotide fragment.

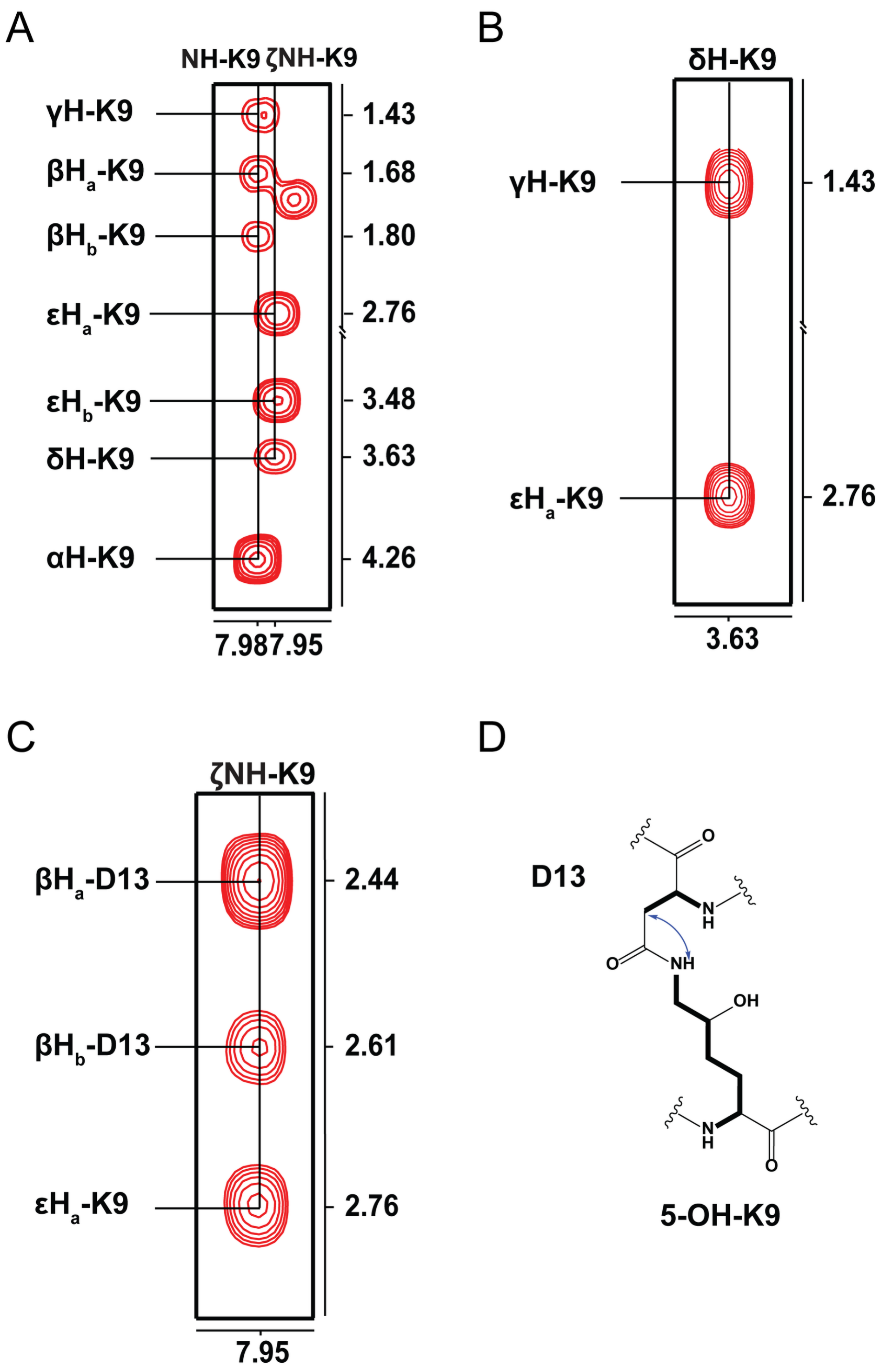

Fig. S46. ^{1}H-^{1}H NMR correlations of corallotide modifications. A) TOCSY correlations of the 5-hydroxyisopeptide bond in the corallotide fragment. B) COSY correlations of the 5-hydroxyisopeptide bond in the corallotide fragment. C) NOESY correlations used to establish the location of the graspetide linkage in corallotide fragment. D) Structure of modifications installed in corallotide.

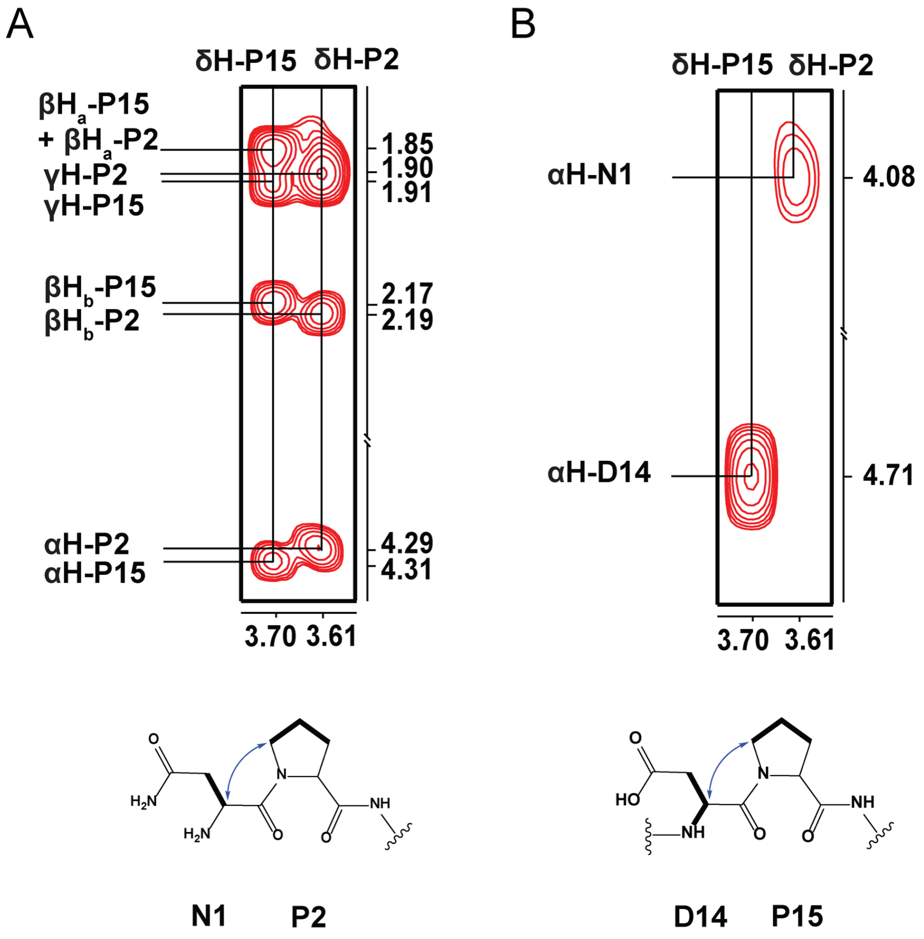

Fig. S47. ^{1}H-^{1}H NOESY correlations of Pro residues in corallotide fragment. A) TOCSY correlations of the proline residues in corallotide fragment. B) NOESY correlations used in assigning each proline residue to the trans configuration.

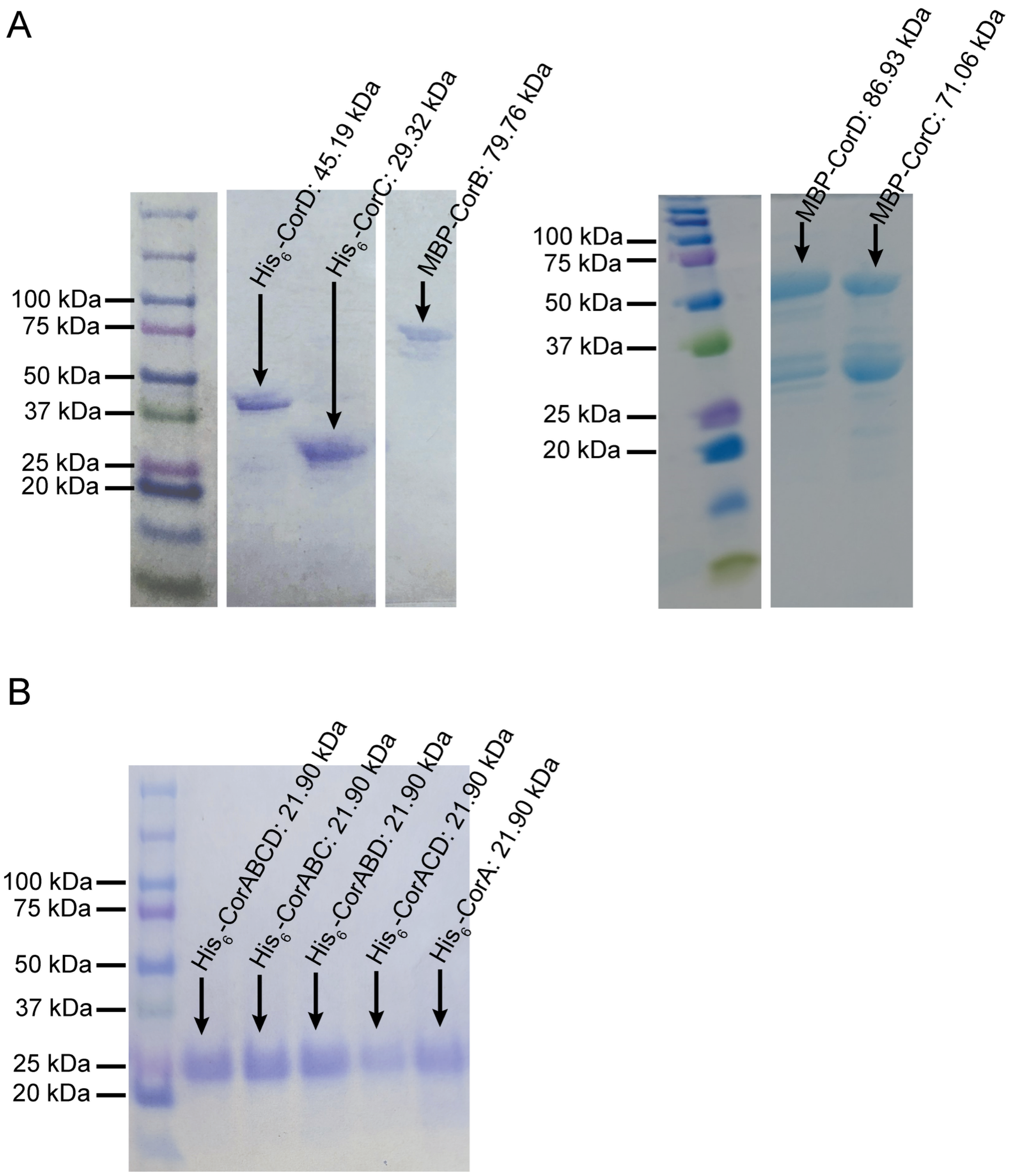

Fig. S48. SDS-PAGE of Cor biosynthetic enzymes and CorA variants. SDS-PAGE of A) corallotide biosynthetic enzymes and B) various CorA intermediates.

A

$[M_1+H]^+$: 1533.74 DGGLVKVMMDDPTR

$[M_2+H]^+$: 2325.26 VKIRMDDPTGGLKLPDFTPPK

$[M_3+H]^+$: 2393.35 GSKLPDLKPLDGGIVKVKMDSPV
2393.27 NDSLKNPDDGVIVKIVADDPLR

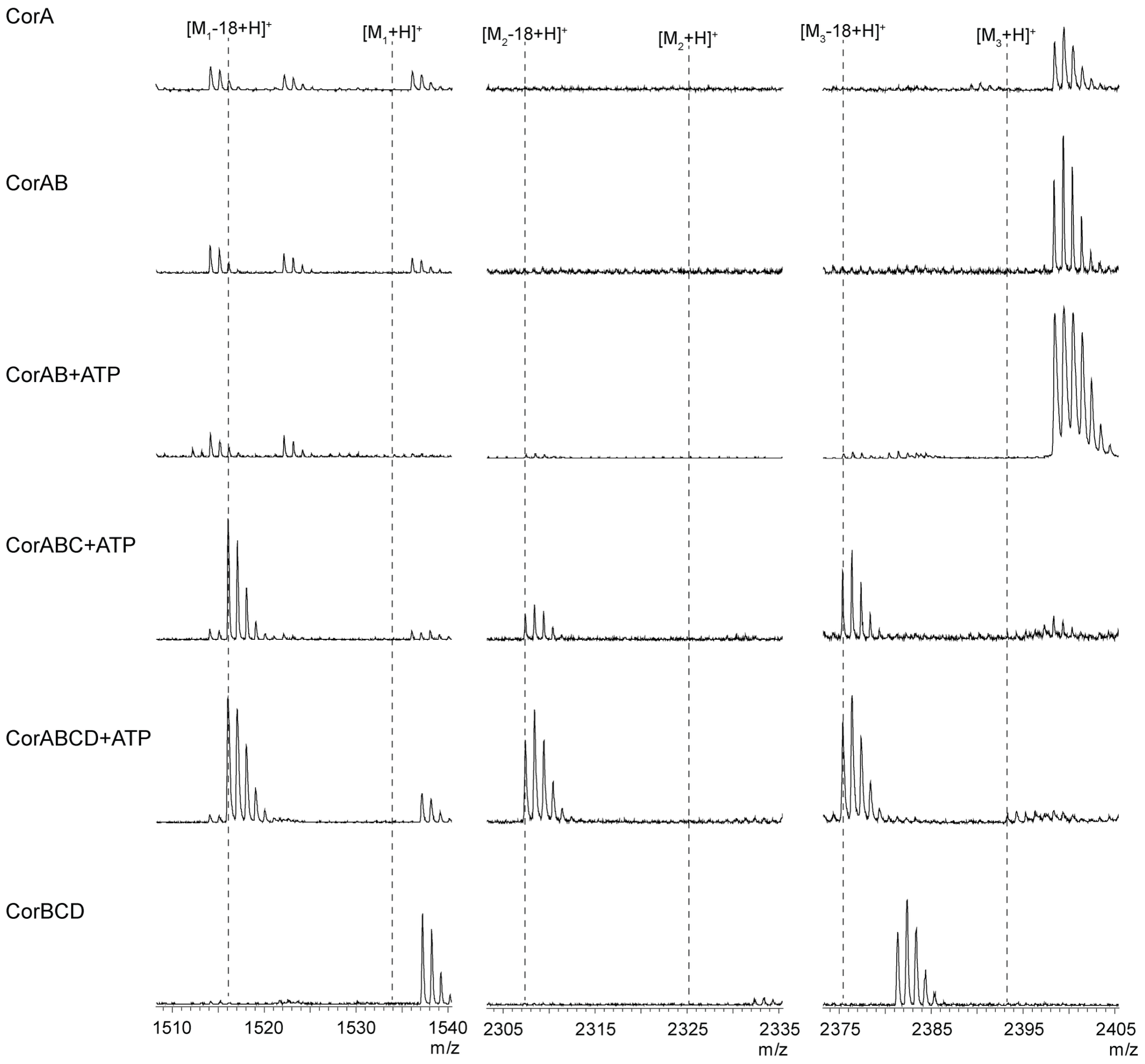

B

$[M_1+H]^+$: 1533.74 DGGLVKVMMDDPTR

$[M_2+H]^+$: 2325.26 VKIRMDDPTGGLKLPDFTPPK

$[M_3+H]^+$: 2393.35 GSKLPDLKPLDGGIVKVKMDSPV
2393.27 NDSLKNPDDGVIVKIVADDPLR

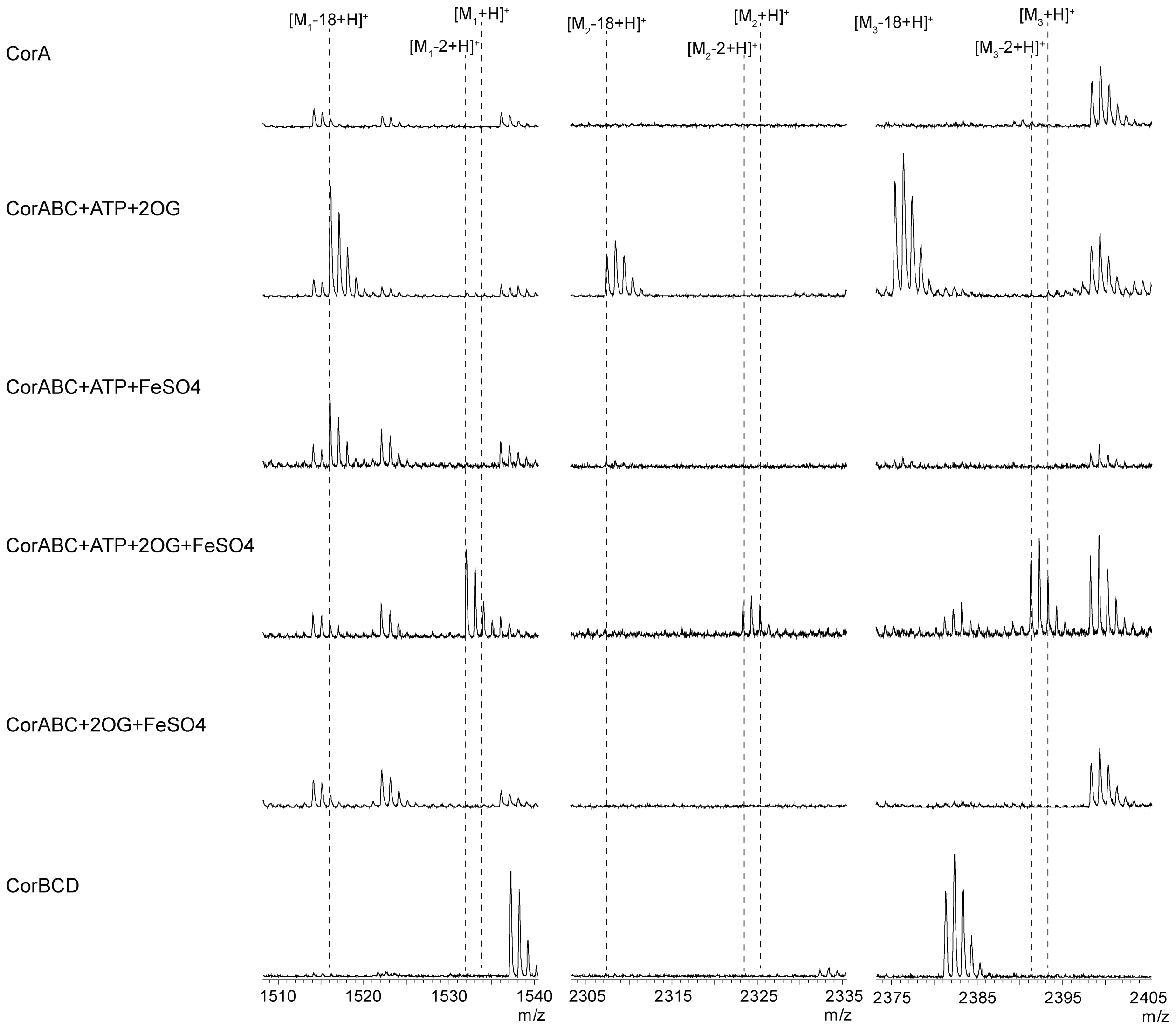

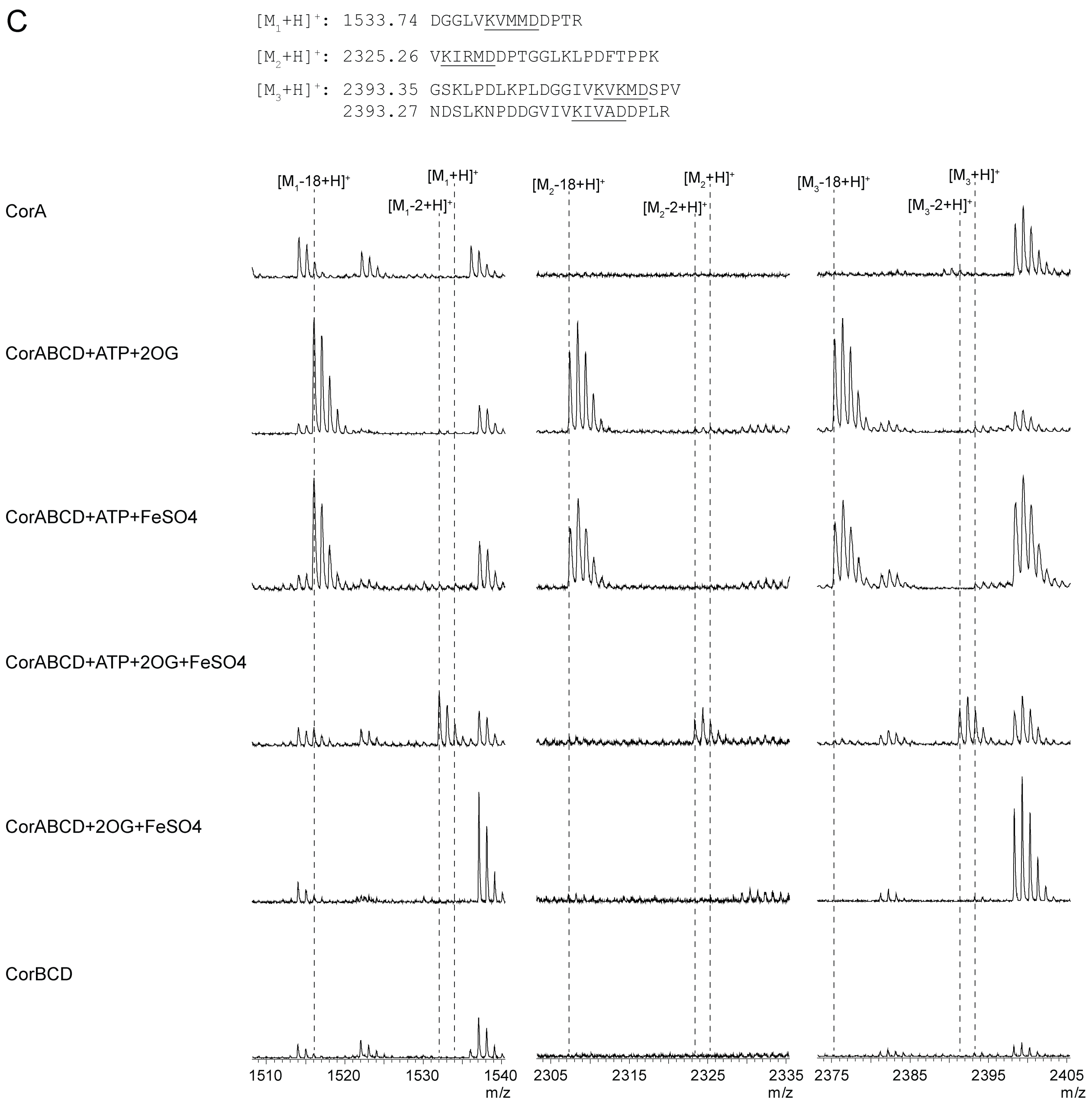
C
[M1+H]+: 1533.74 DGGLVKVMMDDPTR
[M2+H]+: 2325.26 VKIRMDDPTGGLKLPDFTPPK
[M3+H]+: 2393.35 GSKLPDLKPLDGGIVKVKMDSPV
2393.27 NDSLKNPDDGVIVKIVADDPLR
[M1-18+H]+
[M1+H]+
[M1-2+H]+
[M2-18+H]+
[M2+H]+
[M2-2+H]+
[M3-18+H]+
[M3+H]+
[M3-2+H]+
CorA
CorABCD+ATP+2OG
CorABCD+ATP+FeSO4
CorABCD+ATP+2OG+FeSO4
CorABCD+2OG+FeSO4
CorBCD
1510
1520
1530
1540
m/z
2305
2315
2325
2335
m/z
2375
2385
2395
2405
m/z

D

$[M_1+H]^+$: 1533.74 DGGLVKVMMDDPTR

$[M_2+H]^+$: 2325.26 VKIRMDDPTGGLKLPDFTPPK

$[M_3+H]^+$: 2393.35 GSKLPDLKPLDGGIVKVKMDSPV
2393.27 NDSLKNPDDGVIVKIVADDPLR

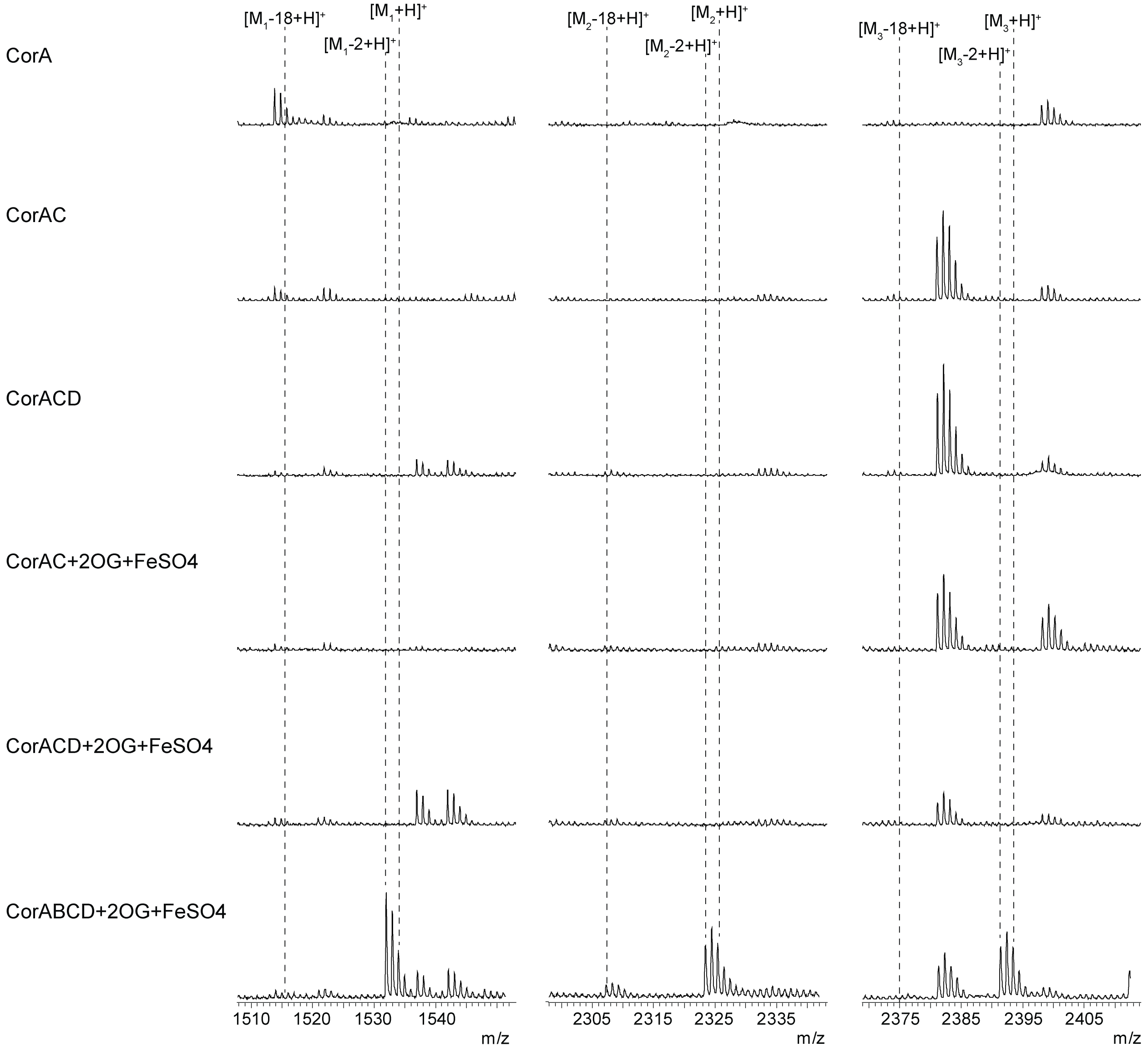

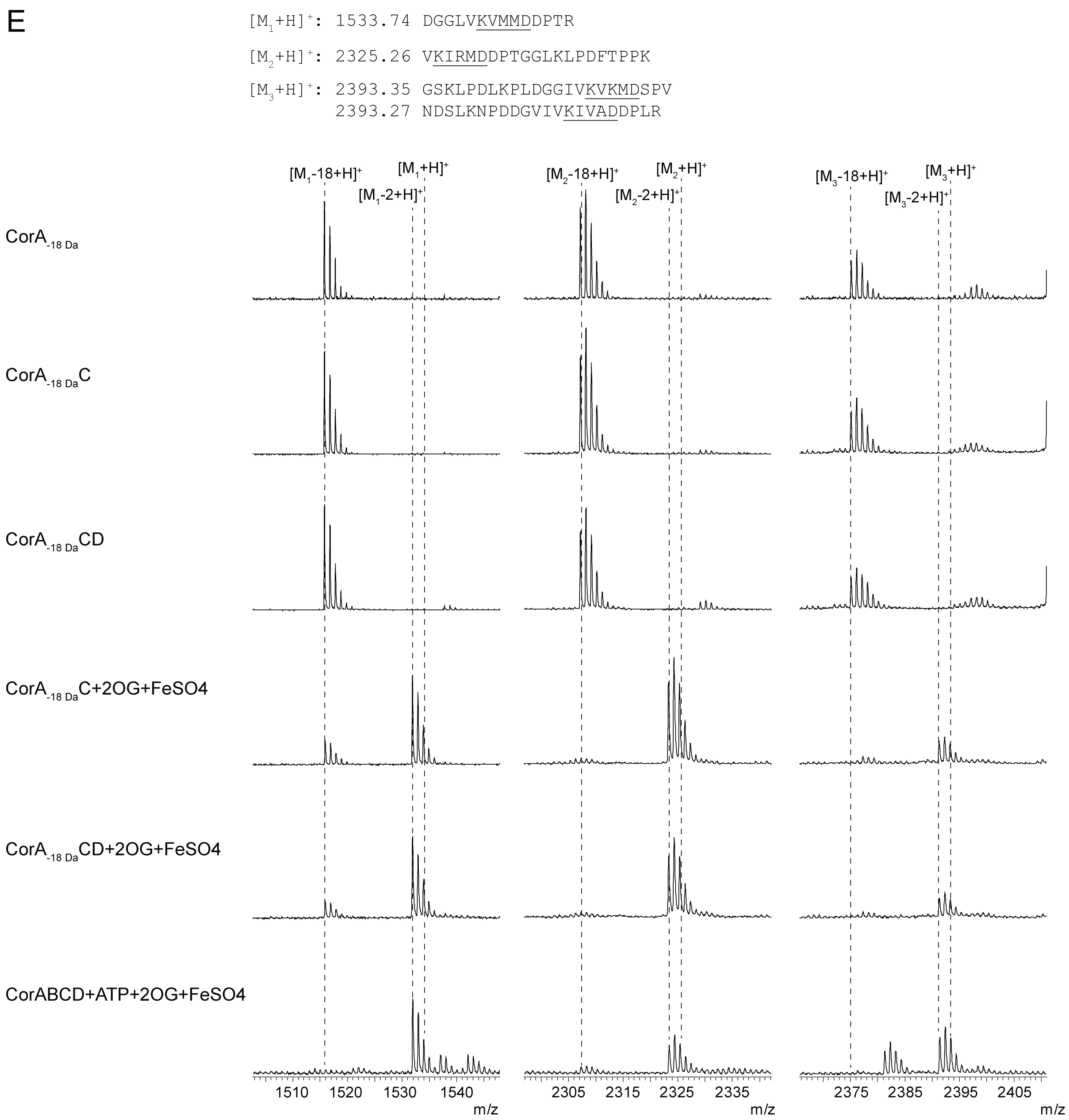

Fig. S49. In vitro reconstitution of Cor biosynthetic enzymes. The activity of the Cor biosynthetic enzymes was reconstituted *in vitro* and investigated using unmodified and cyclized CorA as a substrate. The reactions were digested with trypsin before analysis by MALDI-TOF MS. A) Reconstitution of the graspetide synthetase activity of CorB, which requires the presence of both ATP and CorC. B) Reconstitution of the oxygenase activity of CorC using 2-oxoglutarate (2OG) and Fe(II) ($FeSO_4$ in this study) in the presence of CorB and ATP. C) Reconstitution of the oxygenase activity of CorC in the presence of CorB, CorD, and ATP. D) Unsuccessful reconstitution of CorC on unmodified CorA in the absence of CorB. E) Successful reconstitution of CorC on previously dehydrated CorA in the absence of CorB.

$[M_1+H]^+$: 1429.78 V**KIRMD**DPTGGLK

$[M_2+H]^+$: 2578.38 GKNDSLKNPDDGVIV**KIVAD**DPLR

$[M_3+H]^+$: 2894.58 V**KIRMD**DPTGGLKLPDFTPPKDGGLVK

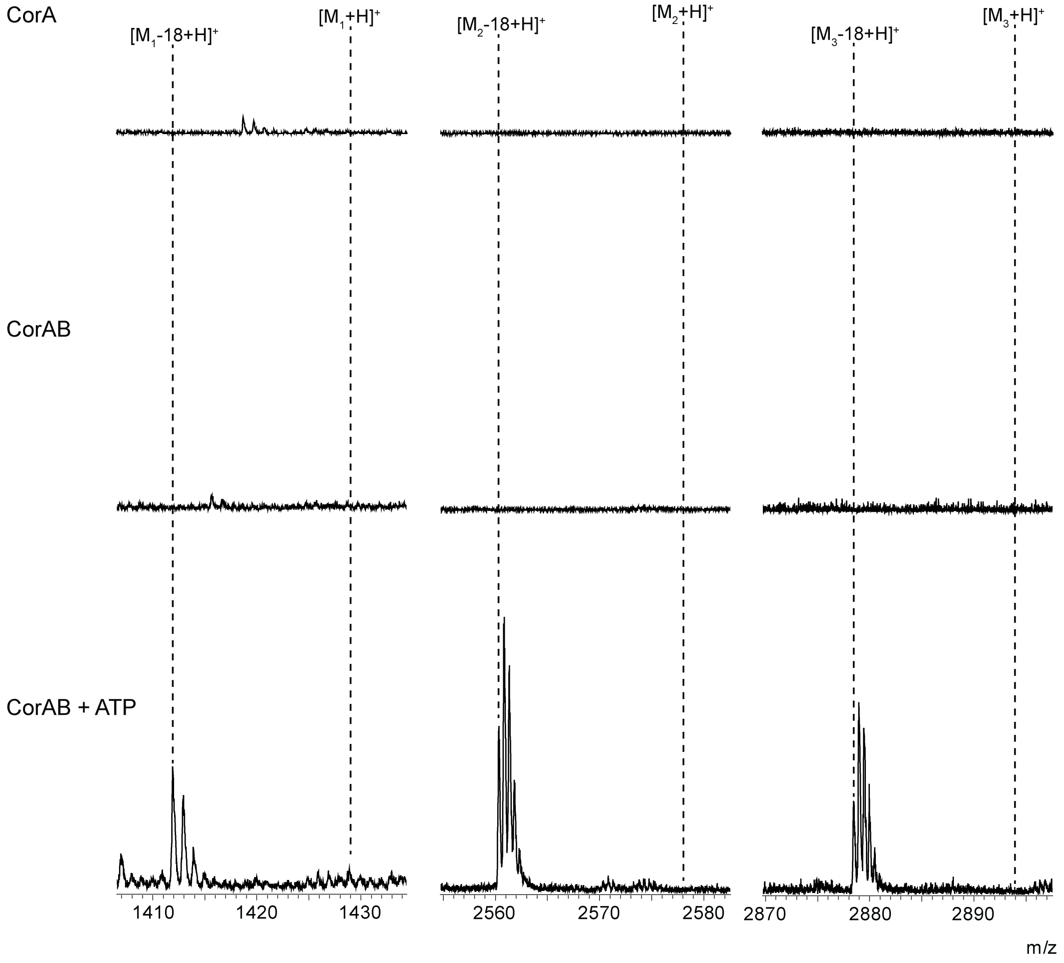

Fig. S50. *In vitro* reactions with 10-fold excess CorB. MALDI-TOF mass spectra for *in vitro* CorAB reactions containing 10-fold excess CorB. The activity of the CorB in the absence of CorC was observed for certain ion fragments.

A

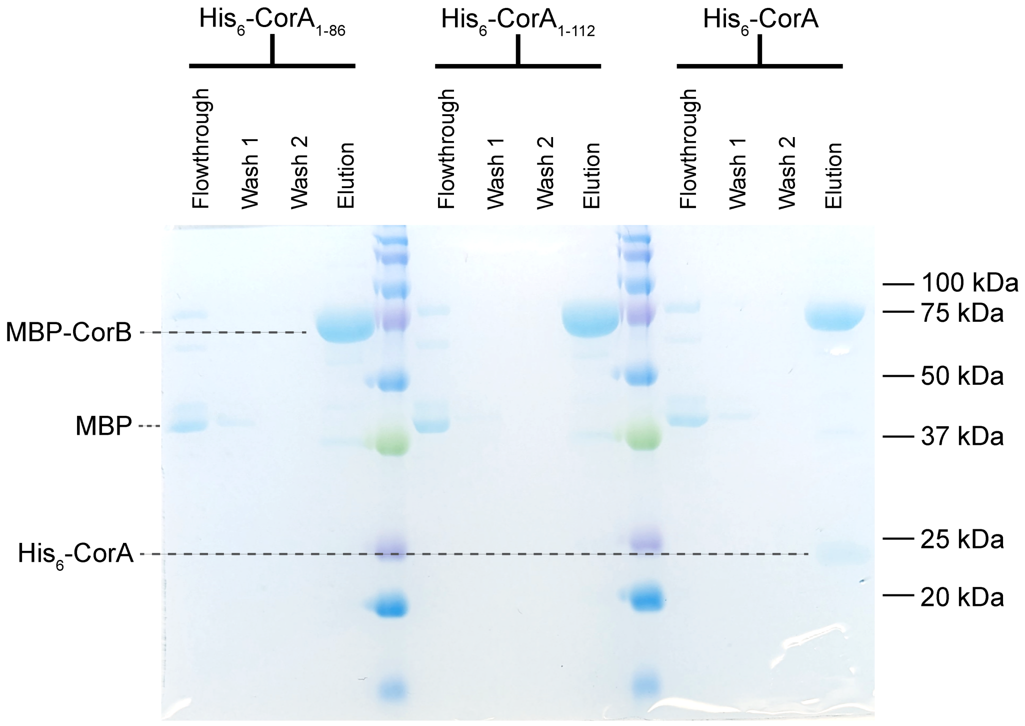
His6-CorA1-86
His6-CorA1-112
His6-CorA
Flowthrough
Wash 1
Wash 2
Elution
MBP-CorB
MBP
His6-CorA
100 kDa
75 kDa
50 kDa
37 kDa
25 kDa
20 kDa

B

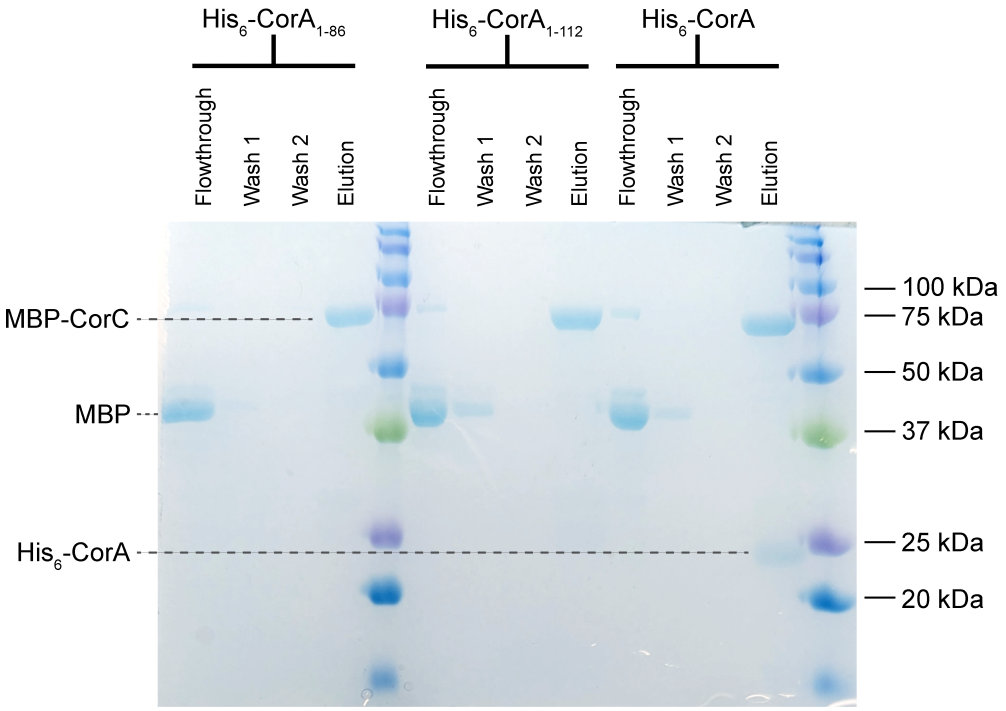
His6-CorA1-86
His6-CorA1-112
His6-CorA
Flowthrough
Wash 1
Wash 2
Elution
MBP-CorC
MBP
His6-CorA
100 kDa
75 kDa
50 kDa
37 kDa
25 kDa
20 kDa

C

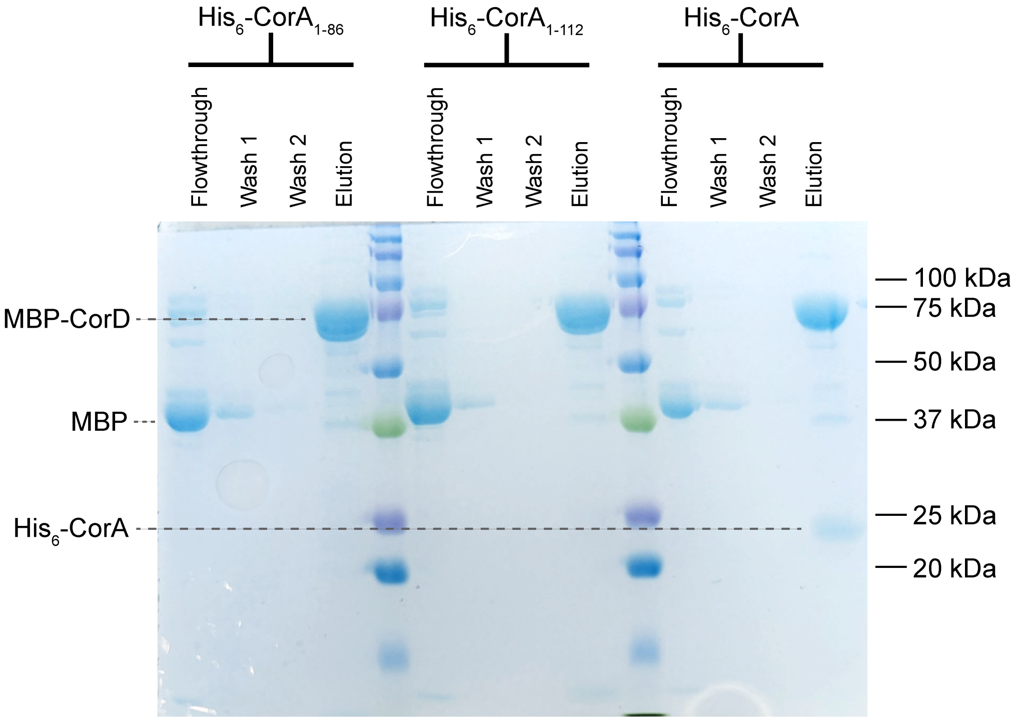

Fig. S51. *In vitro* affinity assay of immobilized His$_6$-CorA variants with Cor proteins. His$_6$-CorA$_{1\text{-}86}$, His$_6$-CorA$_{1\text{-}112}$, and His$_6$-CorA incubated with MBP-tagged A) MBP-CorB, B) MBP-CorC, and C) MBP-CorD, then immobilized on Ni-NTA resin. The immobilized CorA variants were washed, eluted, and analyzed by SDS-PAGE to assess protein-protein interactions.

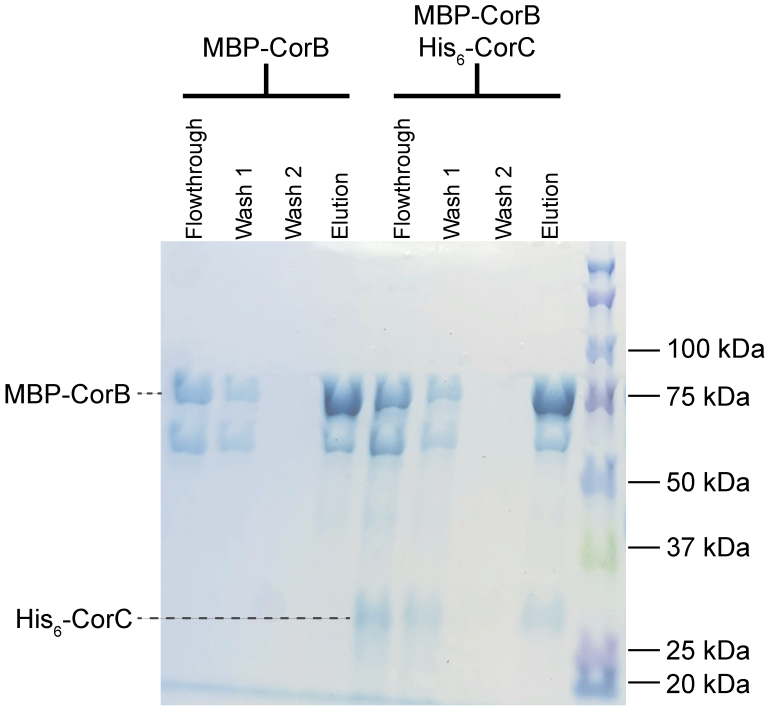

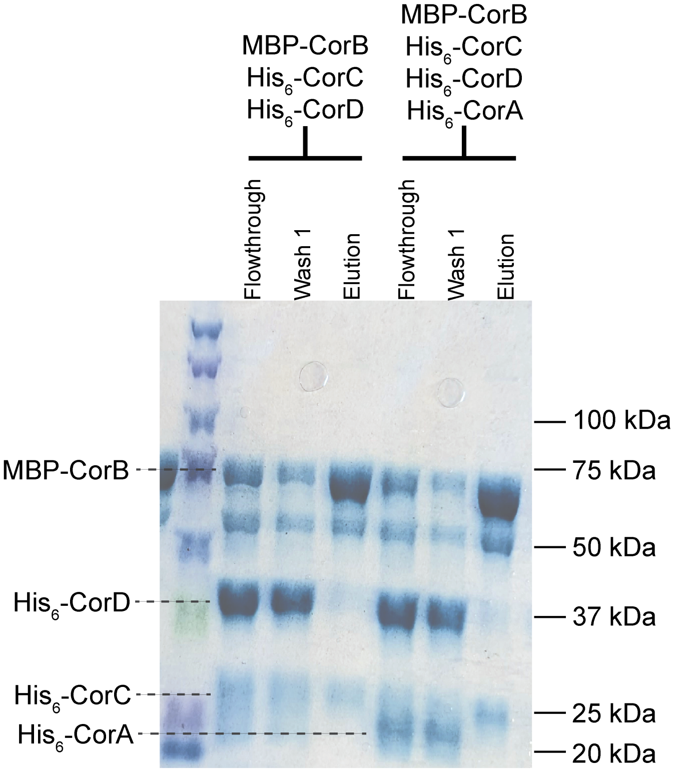

Fig. S52. *In vitro* affinity assay of immobilized MBP-CorB with Cor protein. MBP-CorB was incubated with various Cor proteins and immobilized on amylose resin. The immobilized proteins were washed, eluted, and analyzed by SDS-PAGE to determine if they bound to CorB.

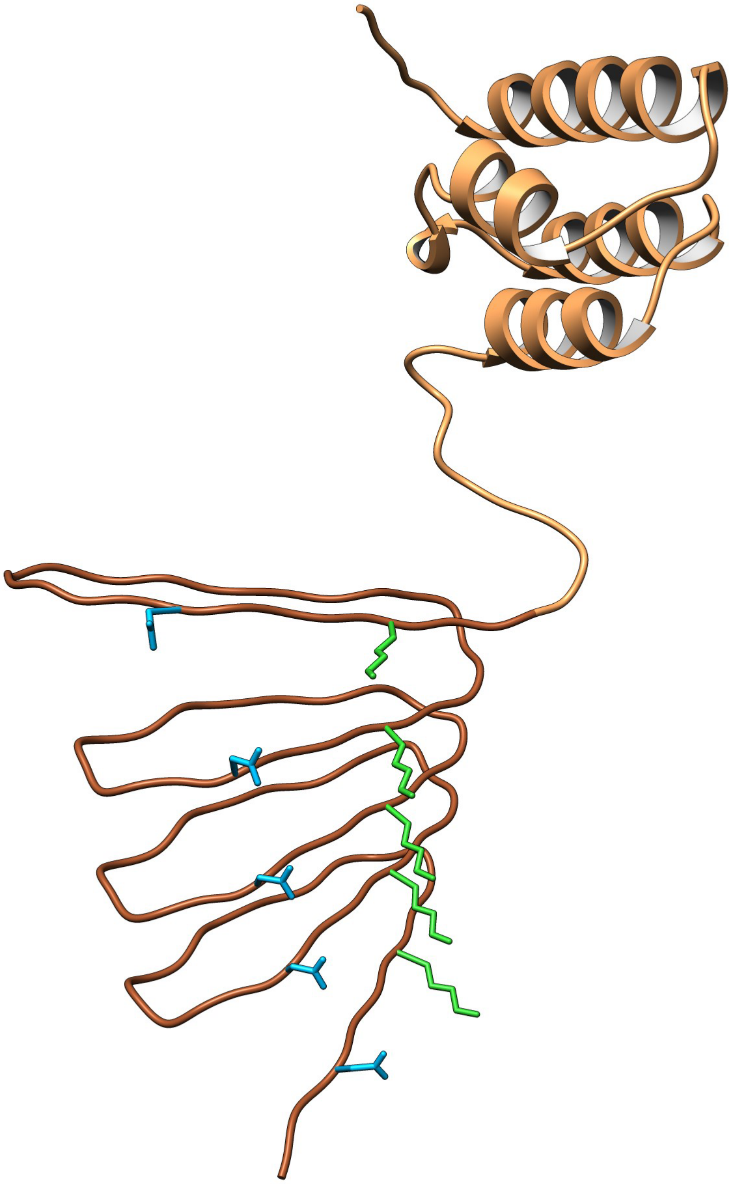

Fig. S53. AlphaFold predicted structure of CorA. The sequence of CorA was input into the AlphaFold3 server to predict its three-dimensional structure (pTM: 0.38). The residues from 1-86 (light brown) are predicted to be a leader sequence and linker, while the remaining residues from 87-199 form an oblong coil (dark brown). The donor (green) and acceptor (blue) residues involved in the graspetide linkage are colored and predicted to be on the same face of coil.

A

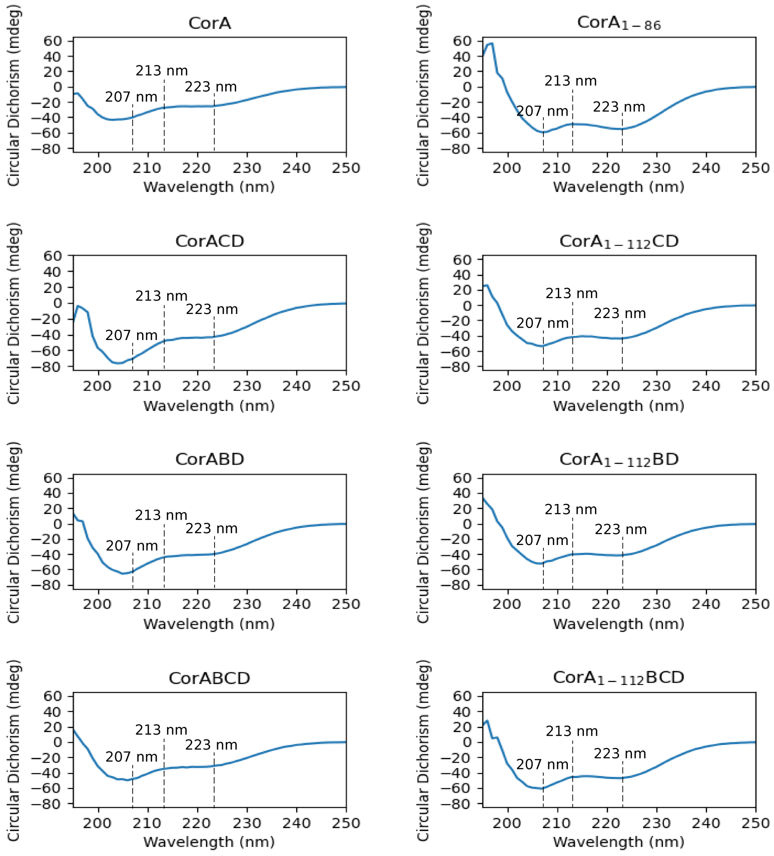

B

	CorA	CorACD	CorABD	CorABCD	CorA$_{1-86}$	CorA$_{1-112}$CD	CorA$_{1-112}$BD	CorA$_{1-112}$BCD
207 nm/ 223 nm	1.57	1.63	1.57	1.52	1.08	1.23	1.26	1.29
207 nm/ 213 nm	1.45	1.44	1.41	1.36	1.22	1.28	1.30	1.33
213 nm/ 223 nm	1.08	1.13	1.11	1.11	0.88	0.96	0.97	0.97

Fig. S54. CorA Circular dichroism spectra. A) CD spectra of various CorA samples. CorA$_{1-86}$ is predicted to be an alpha helical rich leader peptide domain and shows the typical CD spectrum for an alpha helical domain with minima at 207 and 223 nm. The spectra for both CorA and CorA$_{1-112}$ does not appear to change noticeably between samples containing unmodified (CorA, CorACD), dehydrated (CorABD), or dehydrated and oxygenated (CorABCD) products. However, in full length CorA variants the minima around 207 nm appears to shift to 205 nm and deepens more than the leader sequence alone. B) Ratio of the CD signal at certain wavelengths compared between the various samples. The change in the minima around 205-207 nm can be observed measuring the ratio of 207:223 nm.

References

(1) Gerlt, J. A.; Bouvier, J. T.; Davidson, D. B.; Imker, H. J.; Sadkhin, B.; Slater, D. R.; Whalen, K. L. Enzyme Function Initiative-Enzyme Similarity Tool (EFI-EST): A Web Tool for Generating Protein Sequence Similarity Networks. *Biochimica et Biophysica Acta (BBA) - Proteins and Proteomics* **2015**, *1854* (8), 1019–1037. https://doi.org/10.1016/j.bbapap.2015.04.015.

(2) Shannon, P.; Markiel, A.; Ozier, O.; Baliga, N. S.; Wang, J. T.; Ramage, D.; Amin, N.; Schwikowski, B.; Ideker, T. Cytoscape: A Software Environment for Integrated Models of Biomolecular Interaction Networks. *Genome Res.* **2003**, *13* (11), 2498–2504. https://doi.org/10.1101/gr.1239303.

(3) Edgar, R. C. MUSCLE: A Multiple Sequence Alignment Method with Reduced Time and Space Complexity. *BMC Bioinformatics* **2004**, *5* (1), 113. https://doi.org/10.1186/1471-2105-5-113.

(4) Studier, F. W. Protein Production by Auto-Induction in High-Density Shaking Cultures. *Protein Expression and Purification* **2005**, *41* (1), 207–234. https://doi.org/10.1016/j.pep.2005.01.016.

(5) Bobeica, S. C.; Dong, S.-H.; Huo, L.; Mazo, N.; McLaughlin, M. I.; Jiménez-Osés, G.; Nair, S. K.; van der Donk, W. A. Insights into AMS/PCAT Transporters from Biochemical and Structural Characterization of a Double Glycine Motif Protease. *eLife* **2019**, *8*, e42305. https://doi.org/10.7554/eLife.42305.

(6) Shevchenko, A.; Tomas, H.; Havli, J.; Olsen, J. V.; Mann, M. In-Gel Digestion for Mass Spectrometric Characterization of Proteins and Proteomes. *Nat Protoc* **2006**, *1* (6), 2856–2860. https://doi.org/10.1038/nprot.2006.468.
