# Supplementary material for: Discovery of Partner Protein-Dependent Graspetide Biosynthesis
Source: ACS Chem Biol. 2026 Mar 14;21(4):698–709. doi: 10.1021/acschembio.5c00957 (PMC13097080; doi:10.1021/acschembio.5c00957)
Supplement: Supplementary file 2 [file cb5c00957_si_002.zip › Supp_data_3_NMR/Rosaritide_full_spectra_PDF/250721_mirA_10D2O_90H2O_H_750 MHz.pdf]

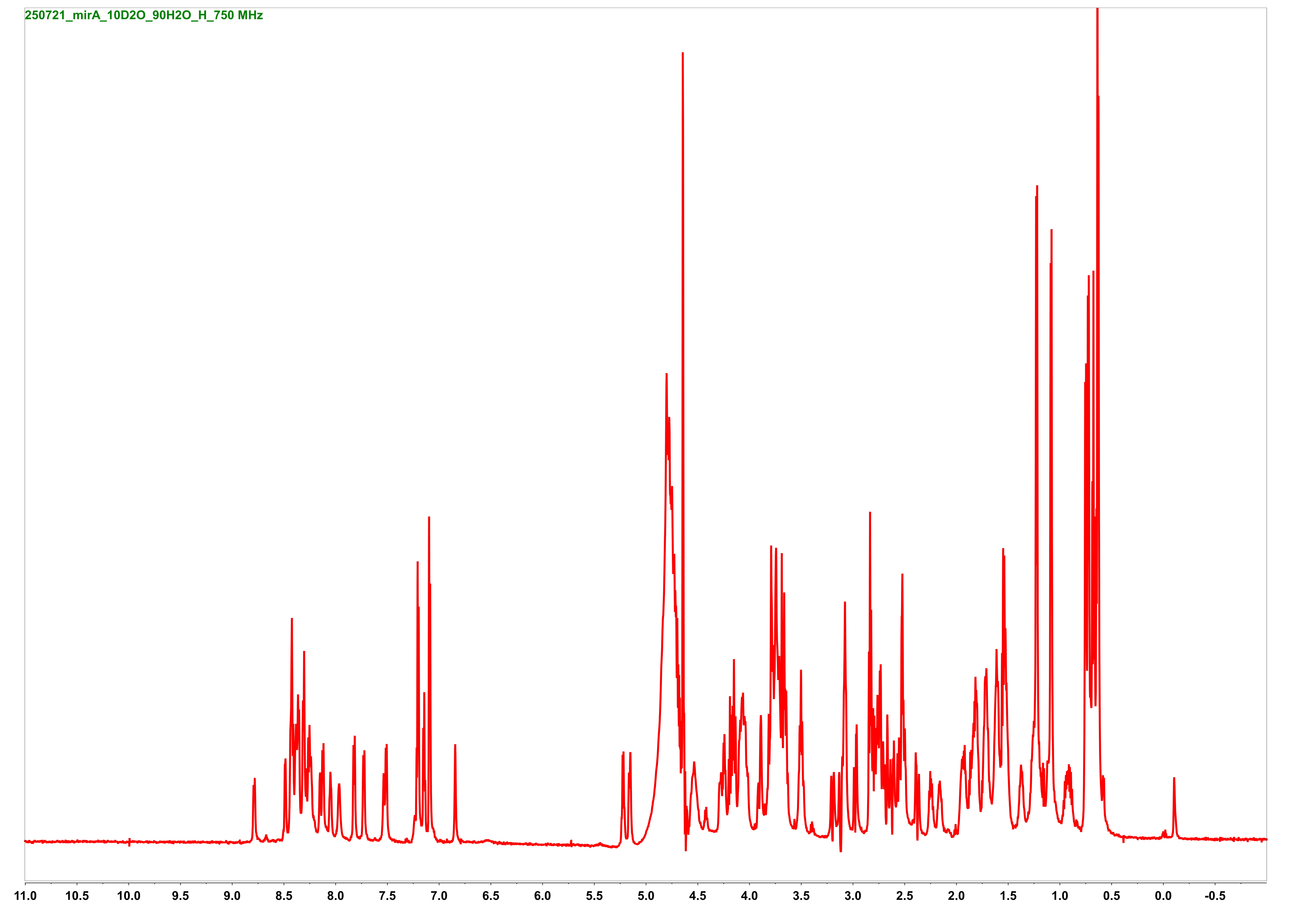
250721_mirA_10D2O_90H2O_H_750 MHz
11.0
10.5
10.0
9.5
9.0
8.5
8.0
7.5
7.0
6.5
6.0
5.5
5.0
4.5
4.0
3.5
3.0
2.5
2.0
1.5
1.0
0.5
0.0
-0.5
