# Supplementary material for: Discovery of Partner Protein-Dependent Graspetide Biosynthesis
Source: ACS Chem Biol. 2026 Mar 14;21(4):698–709. doi: 10.1021/acschembio.5c00957 (PMC13097080; doi:10.1021/acschembio.5c00957)
Supplement: Supplementary file 2 [file cb5c00957_si_002.zip › Supp_data_3_NMR/Rosaritide_full_spectra_PDF/250721_mirA_10D2O_90H2O_HSQC_600 MHz.pdf]

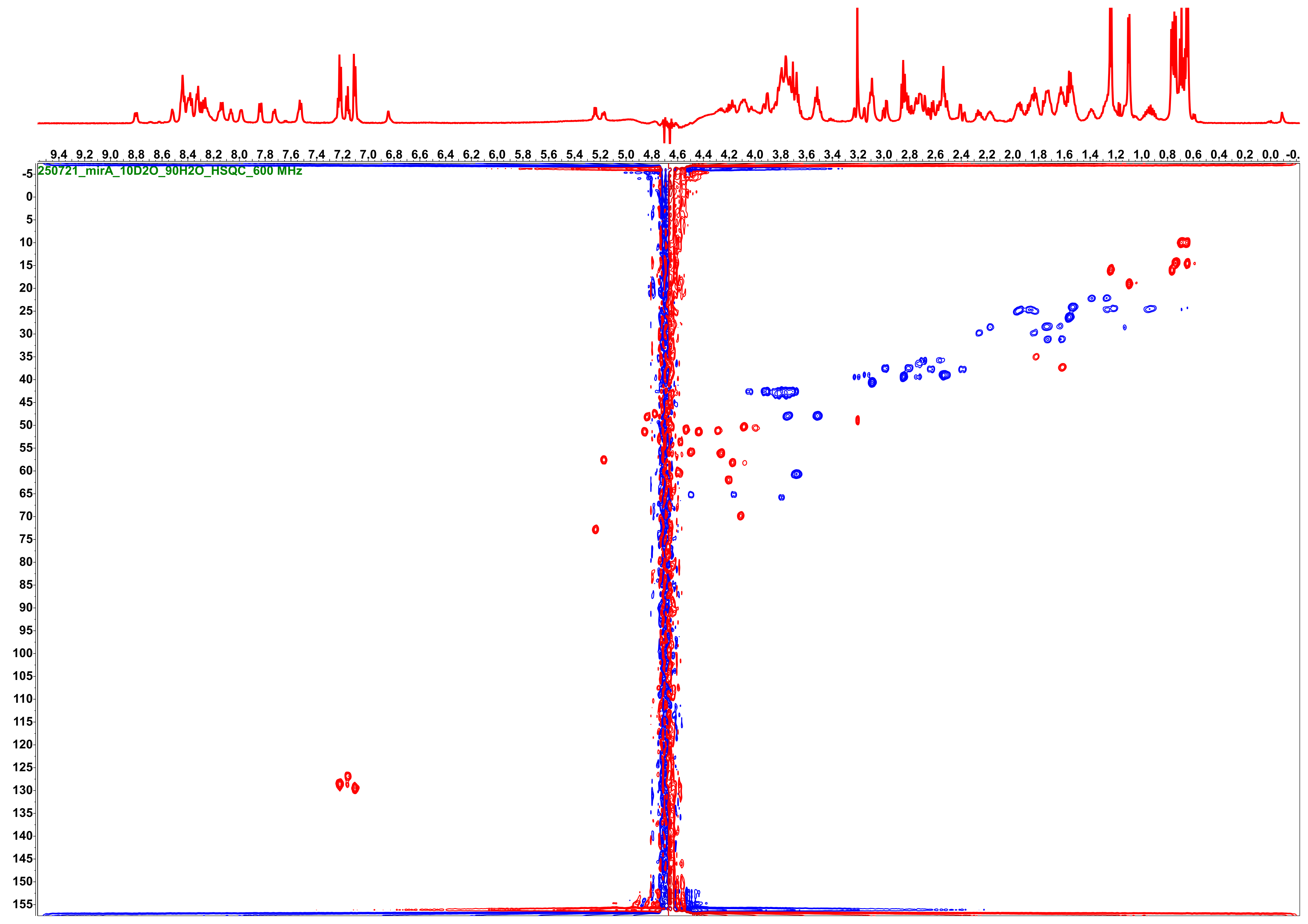

250721_mirA_10D2O_90H2O_HSQC_600 MHz
