# Supplementary material for: Discovery of Partner Protein-Dependent Graspetide Biosynthesis
Source: ACS Chem Biol. 2026 Mar 14;21(4):698–709. doi: 10.1021/acschembio.5c00957 (PMC13097080; doi:10.1021/acschembio.5c00957)
Supplement: Supplementary file 2 [file cb5c00957_si_002.zip › Supp_data_3_NMR/Rosaritide_full_spectra_PDF/250721_mirA_10D2O_90H2O_NOESY_500 msec_750 MHz.pdf]

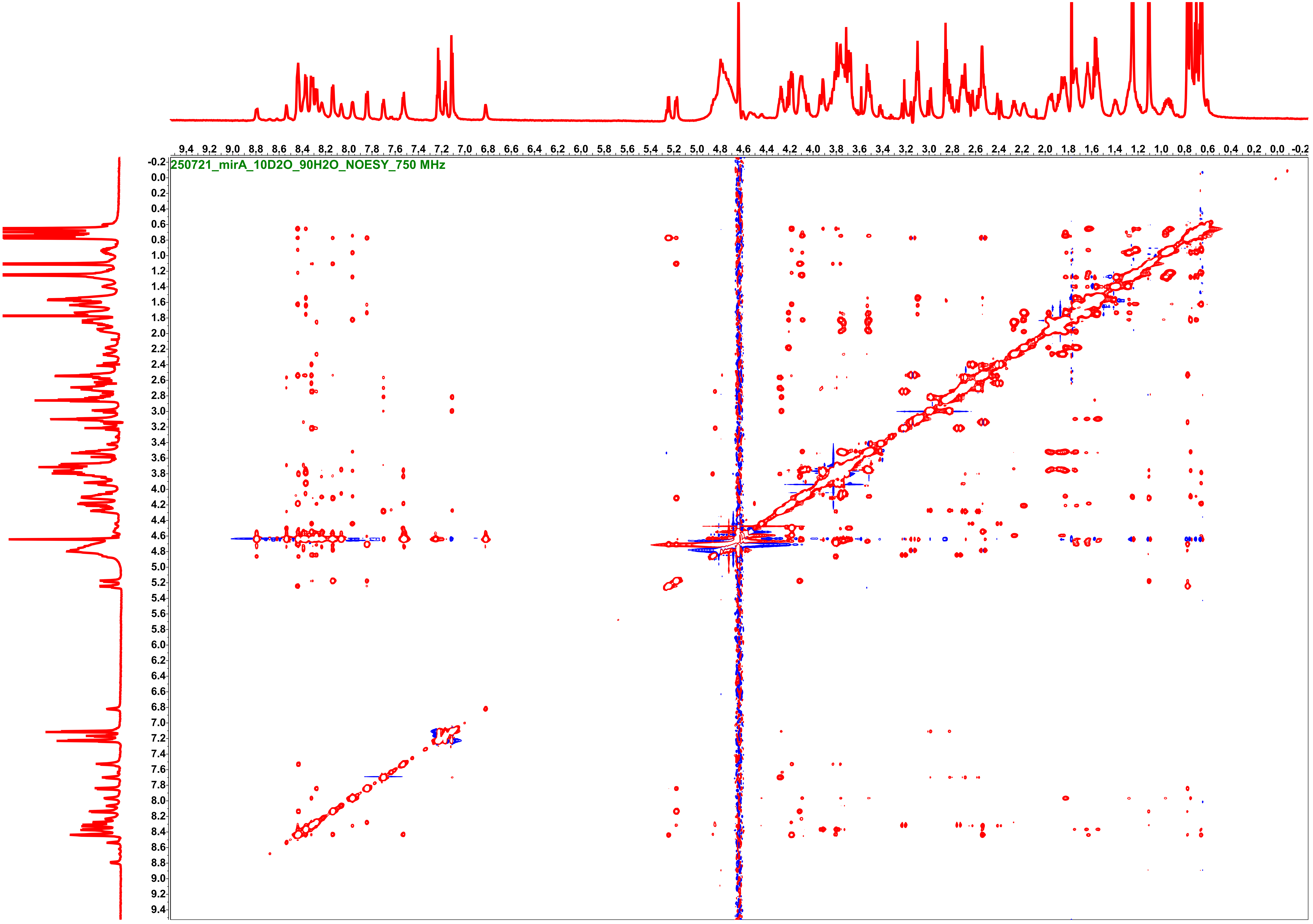
250721_mirA_10D2O_90H2O_NOESY_750 MHz
9.4 9.2 9.0 8.8 8.6 8.4 8.2 8.0 7.8 7.6 7.4 7.2 7.0 6.8 6.6 6.4 6.2 6.0 5.8 5.6 5.4 5.2 5.0 4.8 4.6 4.4 4.2 4.0 3.8 3.6 3.4 3.2 3.0 2.8 2.6 2.4 2.2 2.0 1.8 1.6 1.4 1.2 1.0 0.8 0.6 0.4 0.2 0.0 -0.2
-0.2 0.0 0.2 0.4 0.6 0.8 1.0 1.2 1.4 1.6 1.8 2.0 2.2 2.4 2.6 2.8 3.0 3.2 3.4 3.6 3.8 4.0 4.2 4.4 4.6 4.8 5.0 5.2 5.4 5.6 5.8 6.0 6.2 6.4 6.6 6.8 7.0 7.2 7.4 7.6 7.8 8.0 8.2 8.4 8.6 8.8 9.0 9.2 9.4
